# May Sulfites in Wine Affect Gut Microbiota? An In Vitro Study of Their Digestion and Interplay with Wine Polyphenols

**DOI:** 10.1021/acs.jafc.5c02710

**Published:** 2025-07-23

**Authors:** Edgard Relaño de la Guía, Carolina Cueva, Natalia Molinero, Ana Ruano, M. José Motilva, Begoña Bartolomé, M. Victoria Moreno-Arribas

**Affiliations:** † Institute of Food Science Research (CIAL), CSIC-UAM, c/Nicolás Cabrera 9, Madrid 28049, Spain; ‡ Agri-food Arbitration Laboratory, S. G. Food Quality Control and Agri-Food Laboratories, D.G. of Food, Ministry of Agriculture, Fisheries and Food. Madrid 28014, Spain; § Institute of Grapevine and Wine Sciences (ICVV), CSIC-University of La Rioja-Government of La Rioja, Logroño, La Rioja 26007, Spain

**Keywords:** sulfites, gastrointestinal digestion, gut microbiota dynamics, wine (poly)phenols, simgi

## Abstract

Sulfites are widely used in the wine industry, but their human health effects remain debated. This study is the first to investigate the interaction between wine sulfites and gut microbiota under simulated gastrointestinal conditions. Using the simgi model, red and synthetic wines—with and without SO_2_ (200 mg/L)—underwent gastrointestinal digestion and colonic fermentation with fecal microbiota from three healthy donors (*n* = 3). SO_2_-treated wines slightly modified gut microbiota composition, decreasing beneficial bacteria like *Bacteroides* and *Ruminococcus*, while increasing *Coprococcus* and pro-inflammatory *Escherichia*/*Shigella*, although the overall microbiome of each individual seems to condition its resilience toward SO_2_. These effects were partially mitigated in red wine, suggesting a protective role of wine polyphenols. Additionally, SO_2_ treatment in red wines enhanced phenolic metabolism at the gut level, increasing low-molecular-weight phenolic compounds, such as valerolactones, bioavailable in the small intestine and colon. For instance, 5-(3′,4′-dihydroxyphenyl)-γ-valerolactone concentrations were consistently higher in SO_2_-treated red wine (0.86–1.28 mg/L) than in the untreated wine (<0.78 mg/L) after 6 h of fermentation. This pioneering study reveals a complex interplay between sulfites, wine components, and gut microbiota, with potential health implications, especially for sulfite-sensitive individuals.

## Graphical abstract

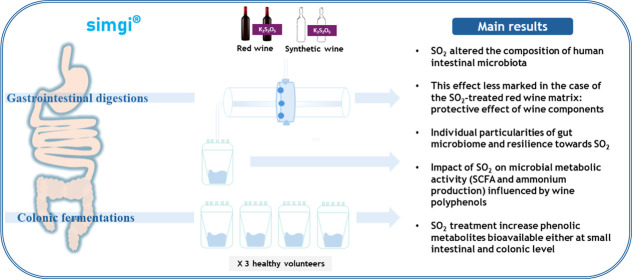

## 1 Introduction

Food additives are essential in food production and processing to ensure safety and prevent the degradation of quality caused by physicochemical, microbial, or enzymatic reactions.^1,2^ Among these, sulfites are widely used in the wine industry for their antimicrobial, antioxidant, and antioxidasic properties, with molecular sulfur dioxide (SO_2_), bisulfite (HSO_3_^–^) and sulfite (SO_3_^2–^) being the most commonly employed forms.^3^ Despite their technological benefits, the use of sulfites has recently been called into question due to their potential effects on human health. Specifically, regulatory agencies, such as the European Food Safety Authority (EFSA), are highlighting the need for new scientific knowledge on the health effects of SO_2_ sulfites (E220-228). In the same line, EFSA has promoted a reevaluation of the risks of exposure to sulfites from food, including wine consumption.^4,5^ Approximately 1% of the population is highly sensitive to sulfites, presenting symptoms such as dermatitis, urticaria, angioedema, diarrhea, abdominal pain, and, in severe cases, bronchoconstriction and anaphylaxis.^6,7^ Additionally, in young adults, the presence of SO_2_ in wine has been linked to headaches in sensitive individuals.^8^ Because of these concerns, there is a growing trend toward reducing sulfite levels and exploring alternative winemaking approaches.^9,10^ Current European legislation limits total SO_2_ content to 150 mg/L for red wines and 200 mg/L for white and rosé wines.^3,11^

Recently, the panel experts of EFSA^5^ reported that although the majority of ingested SO_2_ is metabolized in the liver to sulfate by sulfite oxidases, up to 25% of the ingested dose can bypass first-pass metabolism and circulate systemically. Moreover, the extent to which sulfites reaches the colon depends on various factors, including the ingested dose, the individual’s metabolic state, and the food matrix in which sulfites are consumed, such as in wine.^5^ While healthy individuals generally metabolize sulfites efficiently, those with impaired sulfite oxidase activity, gastrointestinal disorders, or high sulfite intake (e.g., through wine and food consumption) may be more susceptible to alter sulfur metabolism, increasing the likelihood of microbiota-related effects. Therefore, a deeper understanding of the potential effects of sulfites on the host microbiota is needed.

The human gut microbiota, a complex and dynamic microbial ecosystem in the gastrointestinal tract, is increasingly recognized as a critical determinant of health. It contributes to metabolic processes, immune modulation, and in maintaining intestinal homeostasis.^12,13^ Disruptions in gut microbiota composition, or dysbiosis, have been linked to various noncommunicable diseases, including metabolic disorders, inflammatory conditions, and even food intolerances.^14–17^ Dietary components are a major driver of gut microbiota composition and function.^13,18^ Wine polyphenols, in particular, have shown significant potential to modulate gut microbiota favorably, with potential health benefits in preventing digestive and metabolic diseases.^19–22^ However, the effects of other components of the wine matrix, such as additives like sulfites, on gut microbiota during the digestion process remain poorly understood. Preliminary evidence suggests that sulfites may impact microbial communities. For example, at the oral level, SO_2_ concentrations considered safe by regulatory agencies have been shown to alter the composition and richness of the saliva microbiota.^23^ These findings highlight the need to evaluate the effects of sulfites on gut microbiota, particularly within the context of complex food matrices like wine.

Dynamic gastrointestinal simulators, such as simgi, have emerged as valuable tools for studying interactions between food components and gut microbiota. These models offer precision and reproducibility while addressing the ethical and economic constraints of in vivo studies.^24–26^ The simgi system mimics the stomach, small intestine, and three regions of the colon (ascending, transverse, and descending colon), enabling detailed investigations of food digestion and fermentation processes.^27,28^ Recently, simgi has been successfully applied to investigate the gut microbiota-modulating effects of the codigestion of red wine (as a polyphenol-rich food model) with different nutrients, including carbohydrates and proteins,^29^ as well as lipids.^30^

Against this background, the aim of this study was to explore the effects of wine sulfites on human gut microbiota using the simgi system. Synthetic wine (SW), synthetic wine treated with SO_2_ (SW + SO_2_), young red wine (RW), and young red wine treated with SO_2_ (RW + SO_2_) were subjected to simulated gastrointestinal digestion and colonic fermentation. Unlike synthetic wine, young red wine comprises the full wine matrix, particularly its phenolic composition. Changes in gut microbiota composition (microbial counts and 16S rRNA sequencing) and functionality (microbial production of short- and medium-chain fatty acids and ammonium) were assessed. Additionally, the effect of sulfites on the intestinal metabolism of polyphenols, as key bioactive components of red wine, was also evaluated in the presence and absence of SO_2_. As a whole, this study aims to provide new insights into the interplay among sulfites, wine components, and gut microbiota, thereby addressing a critical knowledge gap in food science and health research.

## 2 Materials and Methods

### 2.1 Chemicals

Potassium metabisulfite/disulfite (K_2_S_2_O_5_), ethanol absolute (C_2_H_6_O), dl-tartaric acid (C_4_H_6_O_6_), and acetaldehyde (ethanal, CH_3_CHO) were provided by Sigma-Aldrich (Germany). Pepsin and porcine pancreatin were purchased from Sigma-Aldrich, MERK, USA.

### 2.2 Wine Samples

A synthetic wine (SW), consisting of ethanol (15%) and tartaric acid (0.4%) in water, was prepared in the laboratory and adjusted to pH 3.74. This simplified wine matrix, containing only ethanol and tartaric acid, was used instead of a commercial white wine to isolate the effects of SO_2_ from those of other wine components. By eliminating the complexity of naturally occurring wine constituents, this model allowed for a more direct assessment of sulfite-specific effects on gut microbiota activity.

A young red wine (RW) (var. Tempranillo) was elaborated in the experimental winery and kindly provided by the Institute of Grapevine and Wine Science (ICVV, CSIC, Logroño, Spain). The main parameters in this red wine were: pH = 3.74, alcohol = 15%, total acidity = 7.17 g/L, volatile acidity = 1.96 g/L, free SO_2_ = 2.8 mg/L, and total SO_2_ = 10 mg/L. Additionally, versions of these wines treated with potassium metabisulfite/disulfite (K_2_S_2_O_5_) (0.4 g/L), and named SW + SO_2_ and RW + SO_2_, respectively, for the synthetic wine and red wine.

### 2.3 Simulations of Gastrointestinal Digestion and Colonic Fermentation in Simgi

Gastrointestinal digestions of the four wines were carried out separately using the dynamic gastrointestinal model simgi.^28^ The gastrointestinal digestions in the simgi were performed by using just the stomach and small intestine compartments operating continuously. Simulated gastric fluid (SGF) and simulated intestinal fluid (SIF) were prepared as described by Brodkorb et al.^31^ in sterile conditions. The simulated gastric juice solution consisted of SGF and pepsin (2000 U/mL). This solution was prepared daily and kept at 4 °C until gastric digestion. Simulated pancreatic juice was prepared with 12.5 g/L NaHCO_3_, 6 g/L bile salts (Difco BD, USA), and 0.9 g/L porcine pancreatin. All compounds were dissolved in SIF and the solution was filtered by using a 0.45 mm-pore poly­(ether sulfone) (PES) membrane.^29^

Simgi digestion parameters were selected according to those previously reported by Tamargo et al.^29^ with some modifications. Briefly, the system was preconditioned before each gastrointestinal digestion. The stomach compartment was prefilled with 120 mL of SGF (pH 2.0) and the small intestine with 150 mL of SIF (pH 7.0). Once the system was conditioned, the intake of each wine (100 mL) flowed to the stomach, where it was mixed with the simulated gastric juice (15 mL) by peristaltic movements. During gastric digestion, HCl was added gradually until the pH reached 1.8. To simulate physiological gastric emptying, the Elashoff function^32^ was used to program the gastric emptying flow to the small intestine compartment. Then, the arriving chemo (115 mL) was gradually mixed with 40 mL of pancreatic juice and the previous resident volume. Anaerobic conditions—150 rpm, 37 °C and pH 7.0 ± 0.2—were maintained during the 120 min of intestinal digestion. A total volume of intestinal digest (approximately 305 mL) was recovered in all cases. Aliquots of 80 mL were separated and stored at −20 °C for further colonic fermentation. Aliquots of 2 mL were centrifuged (10,000 rpm, 10 min, 4 °C) and supernatants were filtered through a 0.22 μm filter (Symta, Madrid, Spain) and stored at −20 °C for further analysis of phenolic compounds.

Three independent experiments of colonic fermentation with human fecal microbiota were carried out. For that, three healthy donors were recruited to represent an age range typical of wine and sulfite-containing food consumers: #1 (female, 41 years old), #2 (male, 26 years old), and #3 (female, 46 years old). The inclusion of microbiota from three independent healthy donors was intended to capture interindividual variability to a reasonable extent within a manageable experimental framework, while ensuring the reproducibility and interpretability of the microbial and metabolic responses, as carried out in previous studies.^33,34,65^ The donors had no dietary restrictions and were free from gastrointestinal diseases or antibiotic treatments within the previous six months. As a biological triplicate, each experiment consisted of parallel fermentations of the four gastrointestinal digests (SW, SW + SO2, RW, and RW + SO2) with a freshly prepared fecal slurry^33^ from each donor (#1, 2, or 3), using the four simgi intestinal compartments. Each compartment was filled with 15 mL of fecal slurry, 75 mL of gastrointestinal digest (SW, SW + SO2, RW, or RW + SO2), and 60 mL of colon nutrient medium.^34^ All fermentations were carried out at 37 °C, pH 6.8, and 120 rpm for 48 h under anaerobic conditions. Colon samples were collected at different time points (0, 24, and 48 h) to analyze microbiota composition and functionality (production of fatty acids and ammonium), and phenolic compounds (an extra sample time point at 6 h of incubation was included in this case). An immediately collected sample aliquot (1 mL) was used for microbial counts. The remaining aliquots (3 × 2 mL) were centrifuged (10,000 rpm, 10 min, 4 °C). Pellets were preserved at −80 °C for further microbiota analysis, and supernatants were filtered through a 0.22 μm filter (Symta, Madrid, Spain) and stored at −20 °C for further analysis of phenolic compounds, SCFAs, and ammonium. The experimental protocol with the human fecal samples was approved by the Ethics Committee of the Spanish National Research Council (CSIC), assessed under the internal registration code PID2019-108851RB-C21, and was compliant with the Declaration of Helsinki.

### 2.4 SO_2_ Analysis

The analysis of SO_2_ in wines was carried out according to the official Paul Rankine method (OIV-MA-AS323-04A) with some modifications. Briefly, a sequential process of sample acidification, application of a nitrogen flow, and mixing with hydrogen peroxide solution plus an indicator was used. After the color change, a titration with 0.005 M NaOH solution was employed. Additionally, an analysis of the weakest (combined A) and strongly (combined B) combined SO_2_ was carried out by heating the samples to 100 °C and adding 0.3% H_2_O_2_ solution, respectively. Samples were analyzed in duplicate.

### 2.5 Phenolic Compound Analysis

Wines and thawed samples from gastrointestinal digestions and colonic fermentations were filtered through 0.22 μm PVDF filters (Symta, Spain). An analysis of phenolic compounds (wine polyphenols and phenolic metabolites) was carried out by UPLC-ESI-MS/MS following a previously reported method.^35,36^ The liquid chromatographic system used was a Waters Aquity UPLC (Milford, MA, USA) composed of a binary pump, an autosampler thermostat, a heated column compartment, a photodiode array detector, and an Acquity TQD tandem quadrupole mass spectrometer equipped with a Z-spray electrospray ionization (ESI) source. Quantification was based on internal calibration curves, using 4-hydroxybenzoic acid-2,3,5,6-*d*_4_ as internal standard. Analyses were carried out in duplicate.

### 2.6 Determination of Acetaldehyde

Acetaldehyde (ethanal, CH_3_CHO) in wines and digested samples was determined according to the European Commission method by gas chromatography coupled with flame ionization detection (GC-FID), following the protocol established by Regulation (EC) No 2870/2000.^37^ First, the wine or digested samples were adequately diluting them with a high-purity solvent, such as ethanol or water. Acetaldehyde quantification was based on internal calibration curves, using pentan-3-ol as internal standard. Analyses were carried out in duplicate.

### 2.7 Microbial Community Analyses

#### 2.7.1 Microbial Plate Counting

Immediately after sampling, 10-fold serial dilutions of each colonic sample were plated on different types of media as described in Jiménez-Arroyo et al.^38^ Plate counting was done in triplicate and data are expressed as log (CFU/mL).

#### 2.7.2 DNA Extraction, 16S rDNA Gene Sequencing and Data Processing

Pellets of 2 mL colonic samples (in triplicate) were utilized for total DNA extraction, using a QIAmp Fast DNA Stool MiniKit (Qiagen, Hilden, Germany) following the manufacturer’s instructions with slight modifications.^39^ The total DNA concentration obtained in each sample was determined using a NanoDrop ND-1000 spectrophotometer (NanoDrop Technologies, Thermo Fisher Scientific, USA). Subsequently, the V3–V4 region of the 16S rRNA gene was amplified using the following primers: forward 5′-CCTACGGGNBGCASCAG-3′ and reverse 5′-GACTACNVGGGTATCTAATCC-3′. The Illumina two-step PCR protocol was followed for library preparation, and sequencing was conducted on an Illumina MiSeq instrument (Illumina, USA) performing 2 × 300 bp paired-end reads. RStudio v.1.3.1093 software was employed to process the files with raw reads from the Illumina instrument. A total of 1992 amplicon sequence variants (ASVs) were found. Taxonomic assignment was performed using the Silva v.138 database.^40^ Biodiversity, expressed in terms of α-diversity, was estimated using the ASVs by calculating the Observed, Shannon, and Simpson indices through the “Phyloseq” package. The β-diversity was assessed by employing a Bray–Curtis dissimilarity matrix represented by nonmetric multidimensional scaling (NMDS). Relative abundances of each taxon were calculated for each sample at the phylum, family, genus, and species level.

### 2.8 Microbial Functionality Analyses

#### 2.8.1 Short- and Medium-Chain Fatty Acid Production

As an approximation to evaluate the functionality of colonic microbial communities, short- and medium-chain fatty acids (SCFAs and MCFAs, respectively) produced during in vitro fermentation of each gastrointestinal-digested wine were evaluated according to the methodology described in Silva et al.^41^ Briefly, samples were analyzed using a gas chromatograph (GC) Agilent 6890A (Agilent, Santa Clara, CA, USA) coupled to a flame ionization detector (FID). Agilent MSD Chemstation E.02.00.493 software was used. The chromatographic separation was performed in a DB-WAXetr capillary column (100% polyethylene glycol, 60 m, 0.325 mm i.d. × 0.25 μm film thickness) (Agilent J&W). Helium was utilized as the carrier gas at a flow rate of 1.5 mL/min. The oven temperature was initially held at 50 °C for 2 min, then increased at 15 °C/min to 150 °C, at 5 °C/min to 200 °C, and at 15 °C/min to 240 °C 20 min, and held for 20 min. The total time was 41.3 min. FID temperature was 260 °C. Injection conditions were: 1 μL splitless, 250 °C. Quantitative data were obtained by calculating the peak area of each compound in relation to that of the internal standard (2-methylvaleric acid). Analyses were performed in triplicate.

#### 2.8.2 Microbial Ammonium Production

To determine the proteolytic activity of the microbiota, the ammonium concentrations in colonic fermentation samples were determined using the photometric Spectroquant ammonium reagent test (Merck, Germany). The results, measured at 690 nm, were obtained from a linear regression plot constructed using ammonium standard solution of between 2 and 75 mg NH_4_^+^/L. Simgi colonic samples were diluted with deionized water to adjust their concentration to the kit’s measurement range. All analyses were performed in triplicate.

### 2.9 Statistical Analyses

Statistical analyses were performed using the XLSTAT Statistic software for Microsoft Excel, 2022.3.1 statistical package (Addinsoft-SARL., USA). Analysis of variance (two-way ANOVA) and the Games-Howell post hoc test were used to assess differences in microbial composition and metabolic features (microbial counts, metataxonomic results, SCFA and MCFA production, and phenolic metabolites) at different colonic fermentation times (0, 24, and 48 h) and with different wines (SW, SW + SO2, RW, RW + SO2). A two-way ANOVA test with Tukey post hoc correction was used to evaluate differences in ammonium production. The Kruskal–Wallis test was employed to check significant differences in phenolic compounds between RW and RW + SO2, before and after gastrointestinal digestion, when normality assumptions were not met. Significant differences were calculated considering *p* < 0.05 for all the analyses. With regard to microbial plate counting, from a microbiological point of view, significant differences between values were considered significant when they were statistically significant (*p* < 0.05) and greater than Δlog (CFU/mL) ≥ 1 due to microbiological plate counting limitations.

## 3 Results

Levels of SO_2_ in the SO_2_-treated wines prepared after K_2_S_2_O_5_ doping (0.4 g/L) were checked just before feeding the simgi. In the synthetic wine, all the SO_2_ was found in its free form (199.6 mg/L), while in the case of the SO_2_-treated red wine, it was found mostly in the free fraction (147.2 mg/L), although some (52.8 mg/L) was found in the combined fraction. Accordingly, it was also found that the acetaldehyde concentration in SO_2_-treated red wine was slightly lower (9 mg/L) than in the untreated wine (12 mg/L), whereas acetaldehyde was absent from the synthetic wine (data not shown). Likewise, these first findings highlighted the suitability of synthetic wine for use as a control in the gastrointestinal simulation assays.

Residual SO_2_ content was determined at the end of the simulated gastrointestinal digestion phase. In the SO_2_-treated synthetic wine, 27.5% of the initially added sulfites remained in free form. In the case of the SO_2_-treated red wine, % of residual SO_2_ was quite similar (24.5%), but being in free (13.1%) or combined (11.3%) forms (results not shown). Therefore, a measurable fraction of sulfites reached the colonic stage and was available for interaction with the gut microbiota.

### 3.1 Effect of SO_2_ on the Composition of Intestinal Microbiota

Fecal fermentations in the presence of the gastrointestinal-digested wines were monitored by microbial counting of total aerobes, *Enterobacteriaceae*, total anaerobes, *Enterococcus* spp., lactic acid bacteria, *Clostridium* spp., *Staphylococcus* spp., and *Bifidobacterium* spp. populations (Table 1). In general, the counts indicated a growth of the microbial populations in the first hours of incubation (24 h), with a slight decrease in the number of colony-forming units (CFUs) being noted after 48 h, a decrease that was associated with the depletion of the nutrient medium intrinsic to static incubations.^42^ In comparison to synthetic wine, red wine tended to promote the growth of certain groups (for example, see data for lactic acid bacteria). In relation to SO_2_ addition, results revealed certain differences in the viability of colonic microbial populations, reflecting also the variability of the intestinal microbiota among volunteers (#1, #2, and #3). In the case of Enterobacteriaceae members, significantly (*p* < 0.05) lower levels were found for the SO_2_-treated red wine (RW + SO2) than for the untreated red wine (RW) for volunteer #3 (at 24 h) and volunteer #2 (at 48 h). The same effect was observed for the synthetic wine for volunteer #1 (at 24 h), although for volunteers #2 (at 24 and 48 h) and #3 (at 24 h) the opposite effect was observed. *Clostridium* spp. populations showed lower levels when both SW and RW were treated with SO_2_ at 24 h (volunteer #1). At 48 h, the same trend was observed for red wine (volunteers #1 and #3), but not for synthetic wine (volunteer #1), which showed the opposite behavior. Fewer differential cases between SO_2_-treated wines and their sulfite-free counterparts were observed for total aerobes, total anaerobes, and *Bifidobacterium* spp. populations. Finally, no significant differences between SO_2_-treated and untreated wines were found for the populations of *Enterococcus* spp. and *Staphylococcus* spp. Therefore, microbial viability measurements indicated a slight impact of SO_2_ addition in some gut microbial groups, highlighting the different behavior depending on the wine matrix (red wine vs synthetic wine).

**Table 1:**
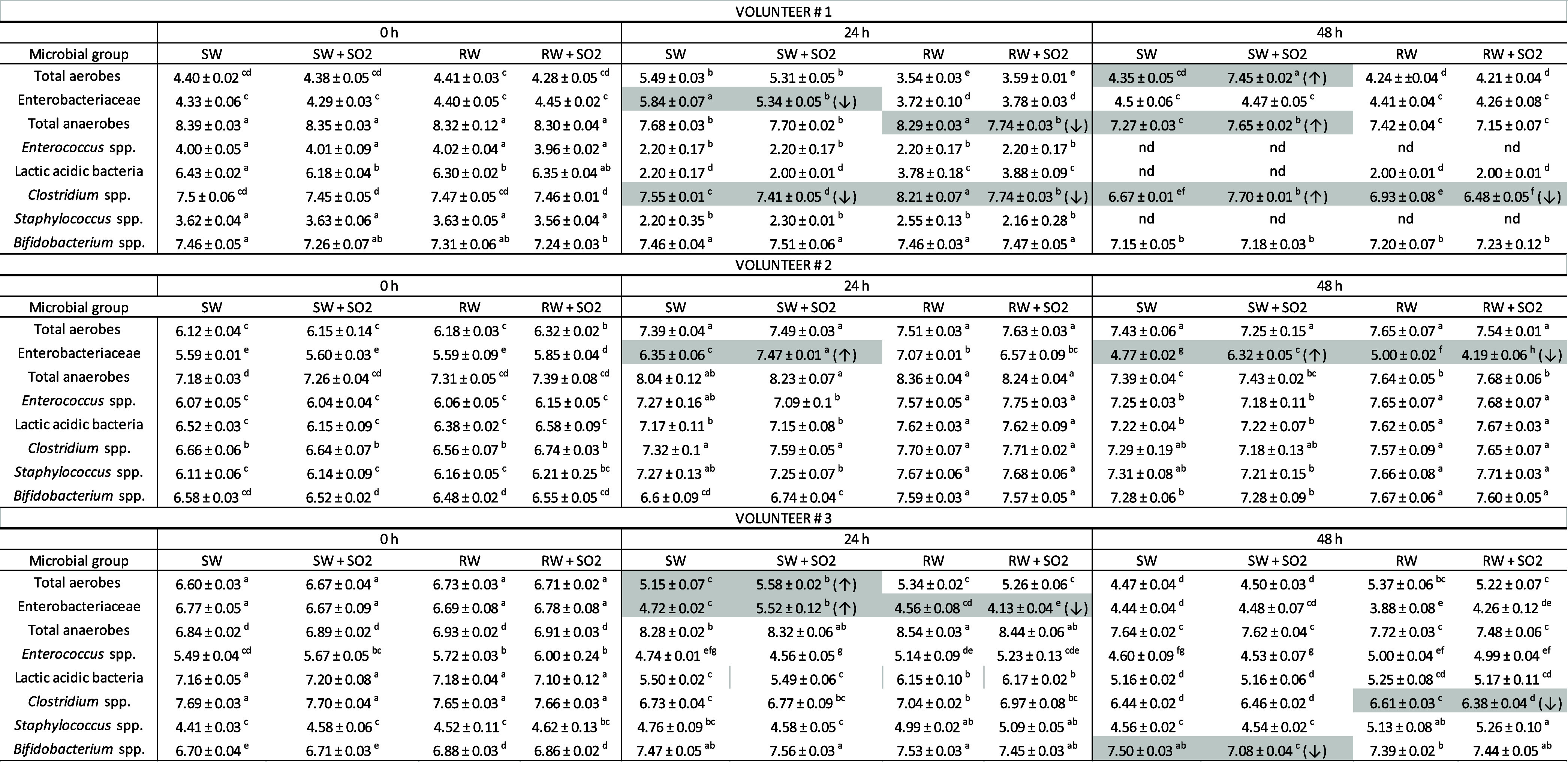
Main Microbial Groups Evaluated by Plate Counting During Fermentations of Gastrointestinal-Digested Wines with Fecal Microbiota from Three Different Volunteers (#1, #2, and #3)^a^

a Data are expressed as mean values of log colony forming units (CFU)/mL. Statistically significant differences, assessed by two-way ANOVA test and Games-Howell correction (*p* < 0.05), are marked by lowercase letters. Shaded cells indicate significant differences between SO_2_-treated wine and its untreated counterpart, and arrows (↑/↓) indicate the direction of change. nd: not detected, SW: synthetic wine, SW + SO2: synthetic wine treated with SO_2_, RW: red wine, RW + SO2: red wine treated with SO_2_.

Complementarily to microbial counts, 16S rRNA gene sequence analysis and a subsequent study of microbial diversity and composition was performed. For the four cases considered (SW, SW + SO2, RW, and RW + SO2) and the three different fecal microbiota (#1, #2, and #3), the indexes of bacterial alpha-diversity (Shannon and Simpson) significantly decreased as fermentation progressed (from 0 to 48 h) (Table S1). However, no significant differences were found in these indexes between fermentations in the presence of SW and RW at equal fermentation times for any of the volunteers. Similarly, the addition of SO_2_ seemed not to lead to differences for either synthetic or red wines. However, in terms of Observed ASVs (Table S1), for SO_2_-treated wines, although not statistically significant, a decreasing trend during fermentation (from 0 to 48 h) in volunteers #1 and #2 was observed for SW, although this effect was not observed in the case of RW, which kept these levels relatively stable during colonic fermentation. In volunteer #3, this effect was not observed; however, an increment in Observed ASVs was noticed at 48 h, which was more obvious in the case of the SO_2_-treated red wine (RW + SO_2_) than in the synthetic one, which also resulted in significant differences between untreated and SO_2_-treated red wines. With regard to beta-diversity, results showed a modulation of the colonic microbial populations during fermentation to a similar profile for both untreated and SO_2_-treated red wines in the three volunteers for all times analyzed, while synthetic wine revealed different microbial profiles depending on its treatment with SO_2_, except for volunteer #3, for which the effect of SO_2_ addition resulted in almost no differentiation (Figure S1). Therefore, changes in both alpha- and beta-diversity derived from SO_2_ treatment in wine seemed to be quite strongly influenced by individual gut microbiota.

Metataxonomic analysis revealed differences in the relative proportions of different taxa for each time period analyzed and depending on the volunteer. At phylum level, an increase in Actinobacteria levels and a decrease in Firmicutes and Bacteroidota were observed during colonic fermentation (from 0 to 48 h) (Figure 1 and Table S2). In addition, there was a gradual and expected increase in the levels of the phyla Proteobacteria and Desulfobacterota, with this being less marked in the case of the red wine matrices (RW and RW + SO2). Specifically, at 48 h, and for volunteers #1 and #3, a statistically significant increase in Proteobacteria level was observed in the presence of SO_2_-treated synthetic wine (SW + SO2) in comparison to its sulfite-free counterpart (SW), which was not observed for the SO_2_-treated red wine (Table S2).

**Figure 1:**
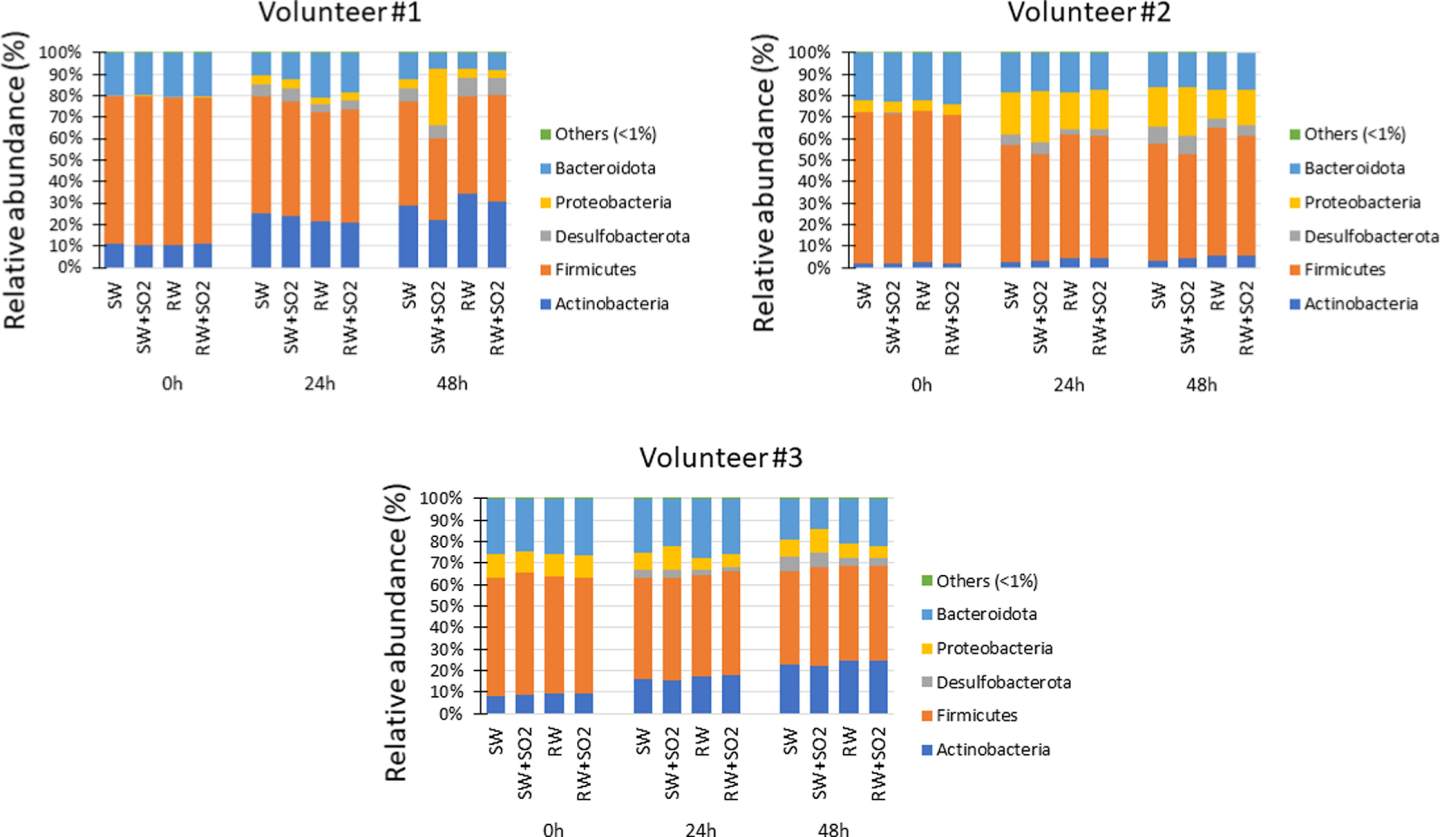
Evolution of relative abundance of taxa at phylum level during colonic fermentation at different times (0, 24, and 48 h) for the four studied wines (SW, SW + SO2, RW, and RW + SO2) and for each volunteer (#1, #2, and #3). Data are expressed as mean values of relative abundance in %. Graphs show the taxa with a relative abundance of >0.1%.

As expected, results at genus (Table S3) and species (Table S4) level reflected interindividual variability to a greater extent. In relation to differences in gender taxa between SO_2_-treated and untreated wines (Table 2), and for synthetic wine, a significant decrease in *Blautia*, *Ruminococcus*, and *Butyricicoccus* levels at 48 h of fermentation with the treated wine (SW + SO2) in volunteer #1 was found, an effect not observed for red wines. Moreover, *Coprococcus* levels increased at 24 and 48 h for SW + SO2, with this increase much less marked in the case of RW + SO2. For volunteer #2, quite similar results were found for all of the studied wines, except for an increase in *Escherichia*/*Shigella* members at 24 and 48 h for SW + SO2 versus SW (15.93% versus 8.94% at 24 h, and 11.82% versus 5.40% at 48 h), a finding partially reduced in the case of RW + SO2 versus RW (11.06% versus 10.34% at 24 h, and 6.71% versus 4.50% at 48 h) (Table S3). For volunteer #3, results also showed an increase in *Escherichia*/*Shigella* members at 24 and 48 h for SW + SO2 versus SW (2.09% versus 1.45% at 24 h, and 0.38% versus 0.16% at 48 h), with RW + SO2 showing the opposite trend at 24 h (0.25% versus 0.63%) (Table S3). Also, an increase was observed in *Parasutterella* levels along with a drop in *Bacteroides* at 48 h for SW + SO2 compared to SW, while SO_2_ presence in the red wine matrix did not exert this effect. In this case, the drop in *Acidaminococcus* levels at 24 and 48 h in the presence of SO_2_ within red wine should be highlighted, as well as the slight decrease in other pathogen-related bacteria such as *Sutterella*.

**Table 2:**
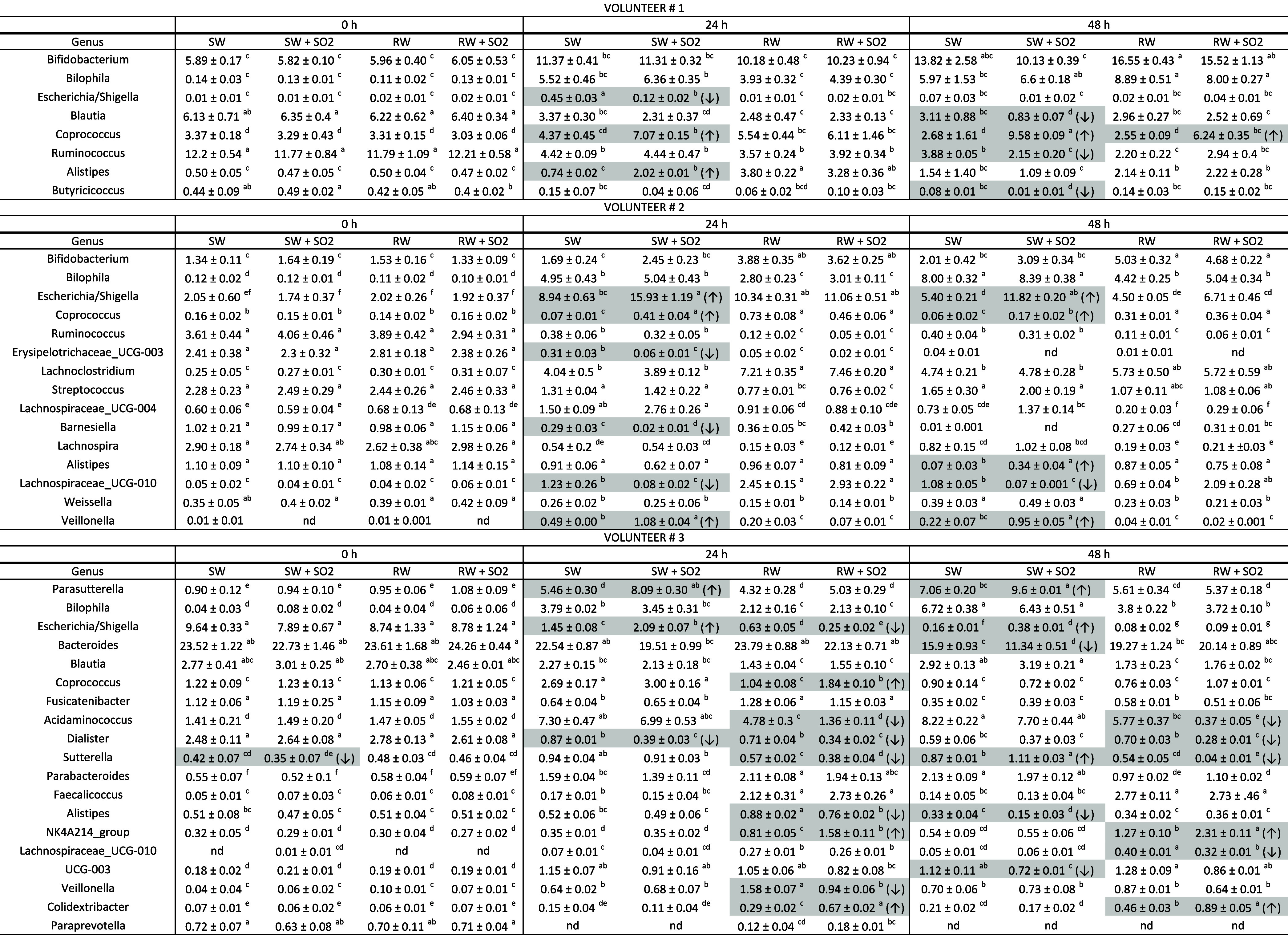
Taxa at Genus Level During Colonic Fermentation at Different Times (0, 24, and 48 h) for the Four Studied Wines (SW, SW + SO2, RW, and RW + SO2) and for Each Volunteer (#1, #2, and #3)^a^

a Data are expressed as mean relative abundance (%) ± standard deviation. Only genera with a mean relative abundance >0.4% in at least one case and showing statistical differences are shown. Statistically significant differences, assessed by two-way ANOVA test and Games-Howell correction (*p* < 0.05), are marked by lowercase letters. Shaded cells indicate significant differences between SO_2_-treated wine and its untreated counterpart, and arrows (↑/↓) indicate the direction of change. nd: not detected, SW: synthetic wine, SW + SO2: synthetic wine treated with SO_2_, RW: red wine, RW + SO2: red wine treated with SO_23_.

Finally, looking in greater depth at changes at species level, results revealed significantly higher levels of *Coprococcus comes* for SW + SO2 than for SW in volunteer #1, an effect also observed when comparing RW + SO2 with RW conditions, albeit much less pronounced (Table S4). Further, SO_2_ treatment of synthetic wine promoted lower levels of gut keystone species associated with positive effects, such as *Parabacteroides distasonis*, *Bacteroides thetaiotaomicron* and *Blautia obeum*, compared to the SO_2_-treated red wine matrix. In volunteer #2, SO_2_ in synthetic wine promoted a slight increase in pathogen- and sulfide-reducing bacteria such as *Alistipes putredinis*, *Coprococcus catus*, and *Veillonella parvula*, together with a drop in commensal taxa such as *Bacteroides coprocola*, while no differences were observed between RW and RW + SO2 conditions (Table S4). For volunteer #3, incubations with SW + SO2 promoted an increase in the levels of some potential pathogen- and protein-degrader bacteria such as species of *Parasutterella* and *Escherichia*/*Shigella*, together with a drop in commensals such as *Bacteroides uniformis*, *Alistipes finegoldii*, and *Bifidobacterium bifidum*, among others. This effect was not observed for SO_2_-treated red wine (RW + SO2), which showed in general a profile similar to that observed for RW, except for *Acidaminococcus intestini*, which revealed a drop in its relative abundance in the presence of SO_2_, and a slight increase in *Colidextribacter massiliensis*, *C. comes*, and *P. distasonis* levels compared to RW incubation (Table S4). All these findings suggested a slight modulating effect of SO_2_ on the abundance of selected minority microbial taxa, which seemed to be minimized in the presence of red wine components.

### 3.2 Effect of SO_2_ on the Metabolic Activity of the Intestinal Microbiota

As means of microbial fermentative activity, short-chain fatty acids (SCFAs) (acetic, propionic, butyric, isobutyric, valeric, and isovaleric acids) were analyzed, as well as medium-chain fatty acids (MCFAs) (hexanoic, heptanoic, octanoic, and decanoic acids), although only acetic, propionic, and butyric acids were detected in quantifiable amounts in all the analyzed samples (Table S5). As expected, the main production of these acids occurs in the first 24 h of incubation with fecal microbiota, with levels being maintained or slightly increased at 48 h (Figure 2). At both times (24 and 48 h), SCFA levels in the fermentations with red wine (untreated or treated with SO_2_) were comparatively higher than in their counterpart fermentations with synthetic wine, with the only exception being that of acetic acid in SO_2_-treated red wine from volunteer #1. On the other hand, the addition of SO_2_ led to different effects on SCFA production depending on the wine matrix considered (red wine vs synthetic wine). Fecal fermentations in the presence of RW + SO2 showed significantly higher values for acetic, propionic, and butyric acids than fermentations in the presence of RW at 24 and/or 48 h for volunteers #2 and #3. However, the opposite behavior was observed for volunteer #1, whose corresponding contents of propionic and butyric acids were significantly lower for RW + SO2 than for RW at 48 h of incubation. As regards synthetic wines, the main effects were observed for volunteer #1, whose corresponding contents of acetic and butyric acids were significantly higher in the presence of SO_2_ (SW + SO2). Therefore, confirming that the fermentative activity of the microbiota was favored by the occurrence of red wine components (i.e., polyphenols) compared to synthetic wine, the results revealed that the individual particularity of the microbiota seemed to condition the impact of sulfites in relation to SCFA production.

**Figure 2:**
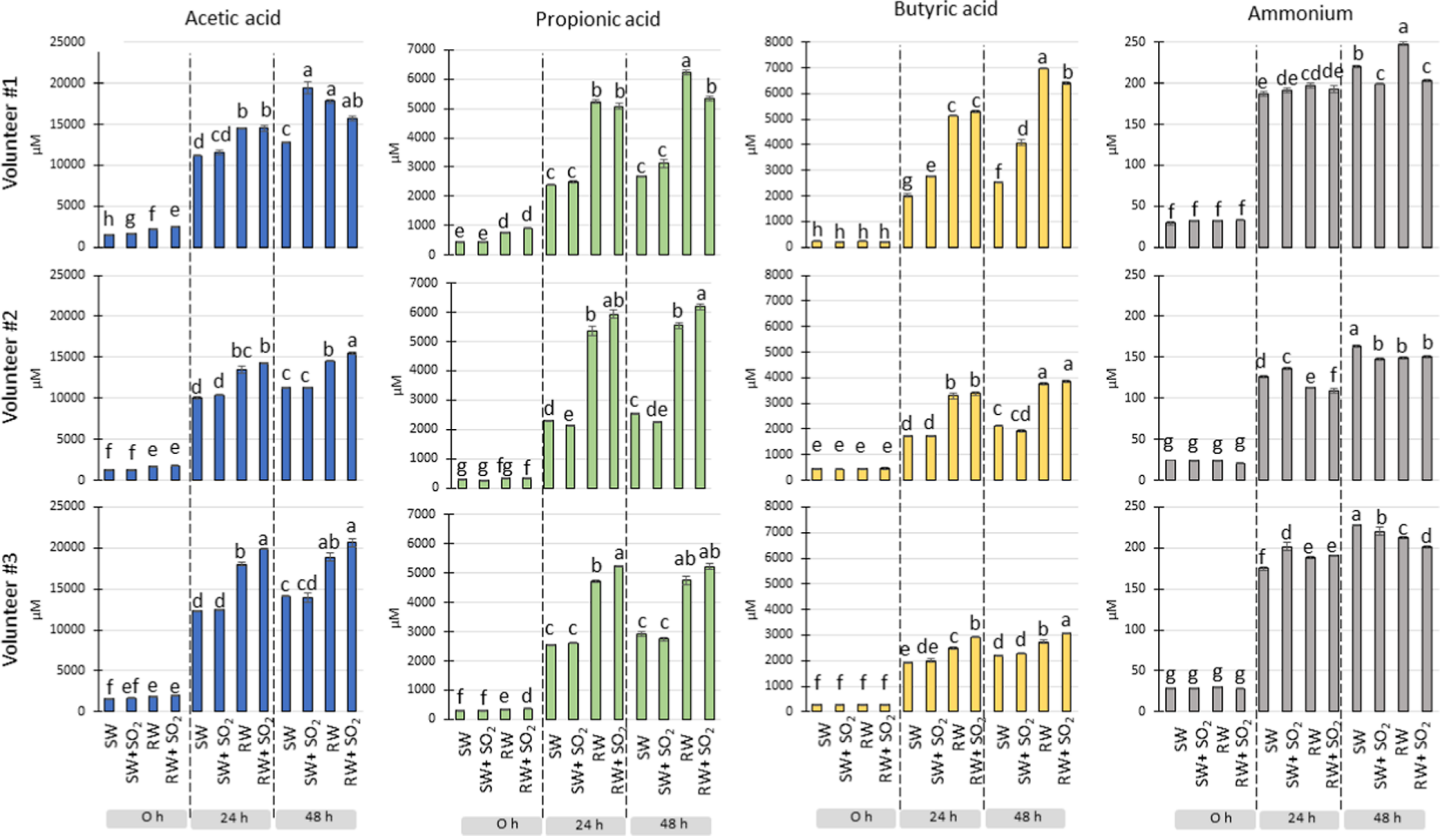
Production of short-chain fatty acids (mM) and ammonium (mg/L) at the end point (48 h) of colonic fermentation for the four studied wines (SW, SW + SO2, RW, and RW + SO2) and for each volunteer (#1, #2, and #3). Data are expressed as mean values ±standard deviation. Lowercase letters denote significant differences between treatments at the colonic fermentation time.

Microbial proteolytic activity (measured as ammonium production) was accelerated during the first 24 h of incubation, with levels being maintained or slightly increased at 48 h (Table S6 and Figure 2). However, in contrast to SCFA concentration, there was no general trend in the comparison of ammonium levels between fermentations with red wines (untreated or treated with SO_2_) and with synthetic wines, at both 24 and 48 h. On the other hand, the addition of SO_2_ seemed to influence ammonium concentration, depending on the fermentation time considered. At 24 h, almost no significant levels were found in untreated or SO_2_-treated wines for either red or synthetic wines, with the exception of SW for volunteers #2 and #3. However, at 48 h, lower levels of ammonium were found in SO_2_-treated wines than in their counterpart untreated wines, for both SW and RW and the three volunteers. Therefore, and under the colonic fermentation model used in this study, sulfites in wine appeared to decrease the overall proteolytic activity of the gut microbiota in longer fermentation periods (48 h), in which some nutrient depletion may have occurred leading to adaptative changes in intestinal microbiota. Minor microbial metabolites such as hydrogen sulfide (H_2_S) were not evaluated in this paper since our goal was to assess the overall microbial response to bisulfite exposure using global biomarkers (i.e., short- and medium-chain fatty acids and ammonium). Besides, microbially-produced H_2_S does not originate solely from inorganic sulfites, but also from the breakdown of organic sulfur amino acids like cysteine, methionine, and taurine.^43^

### 3.3 Effect of SO_2_ on the Metabolism of Wine Polyphenols in the Gut

Wine polyphenols were analyzed before and after gastrointestinal digestion (Table S7). Among the different phenolic classes determined, anthocyanins were found to show a significantly higher content in the SO_2_-treated red wine (RW + SO2) than in the untreated wine (RW). On the other hand, the relatively lower content of gallic acid in RW + SO2 with respect to RW should be noted. After gastrointestinal digestion, a notable decrease in the content of phenolic compounds in comparison to initial wines was observed for both RW and RW + SO2, due to the dilution inherent in the in vitro gastrointestinal simulation, similarly to previous studies using the same gastrointestinal simulator.^29^ In any case, the gastrointestinal digest from the SO_2_-treated wine showed a significantly higher concentration of many phenolic compounds than the untreated wine, particularly in terms of flavan-3-ols and anthocyanins.

As expected, colonic fermentation of the digested wines led to the catabolism of all these phenolic precursors into microbial-derived phenolic metabolites such as phenyl-γ-valerolactones [5-(3′-hydroxyphenyl)-γ-valerolactone and 5-(3′,4′-dihydroxyphenyl)-γ-valerolactone], phenylvaleric acids [4-hydroxy-5-(3′-hydroxyphenyl) valeric acid and 4-hydroxy-5-(3′,4′-dihydroxyphenyl) valeric acid], phenylacetic acids (3-hydroxyphenylacetic acid, 4-hydroxyphenylacetic acid, 3,4-dihydroxyphenylacetic acid and phenylacetic acid), phenylpropionic acids [3-(3′-hydroxyphenyl)­propionic acid and phenylpropionic acid], cinnamic acid (*p*-coumaric acid), and benzoic acids (4-hydroxybenzoic acid, salicylic acid, protocatechuic acid, vanillic acid, 4-*O*-methylgallic acid, and syringic acid) (Table S8). With the exception of some benzoic acids initially present in the gastrointestinal digests, the production of metabolites occurred sequentially over time, with the first detection of valerolactones (6 h) preceding the generation of phenylvaleric acids, phenylacetic acids, and phenylpropionic acids (24–48 h). In general, and despite individual variability, higher contents of wine polyphenol-derived metabolites were detected in the colonic digest of SO_2_-treated red wine than in its counterpart without SO_2_, being statistically significant in many compounds. Figure 3 illustrates specific trends for 5-(3′,4′-dihydroxyphenyl)-γ-valerolactone, 4-hydroxy-5-(3′-hydroxyphenyl)­valeric acid, 3-(3′-hydroxyphenyl)­propionic acid, and phenylpropionic acid. For example, in the case of 5-(3′,4′-dihydroxyphenyl)-γ-valerolactone, significant differences were observed between the untreated wine (<0.78 mg/L) and the SO_2_-treated red wine (0.86–1.28 mg/L) for the three donors after 6 h of incubation (Figure 3). Therefore, under the conditions carried out in this study for simulating gastrointestinal digestion of red wine, SO_2_ treatment seemed to slightly increase the concentration of low-molecular-weight phenolic compounds bioavailable both at small intestinal and colonic levels.

**Figure 3:**
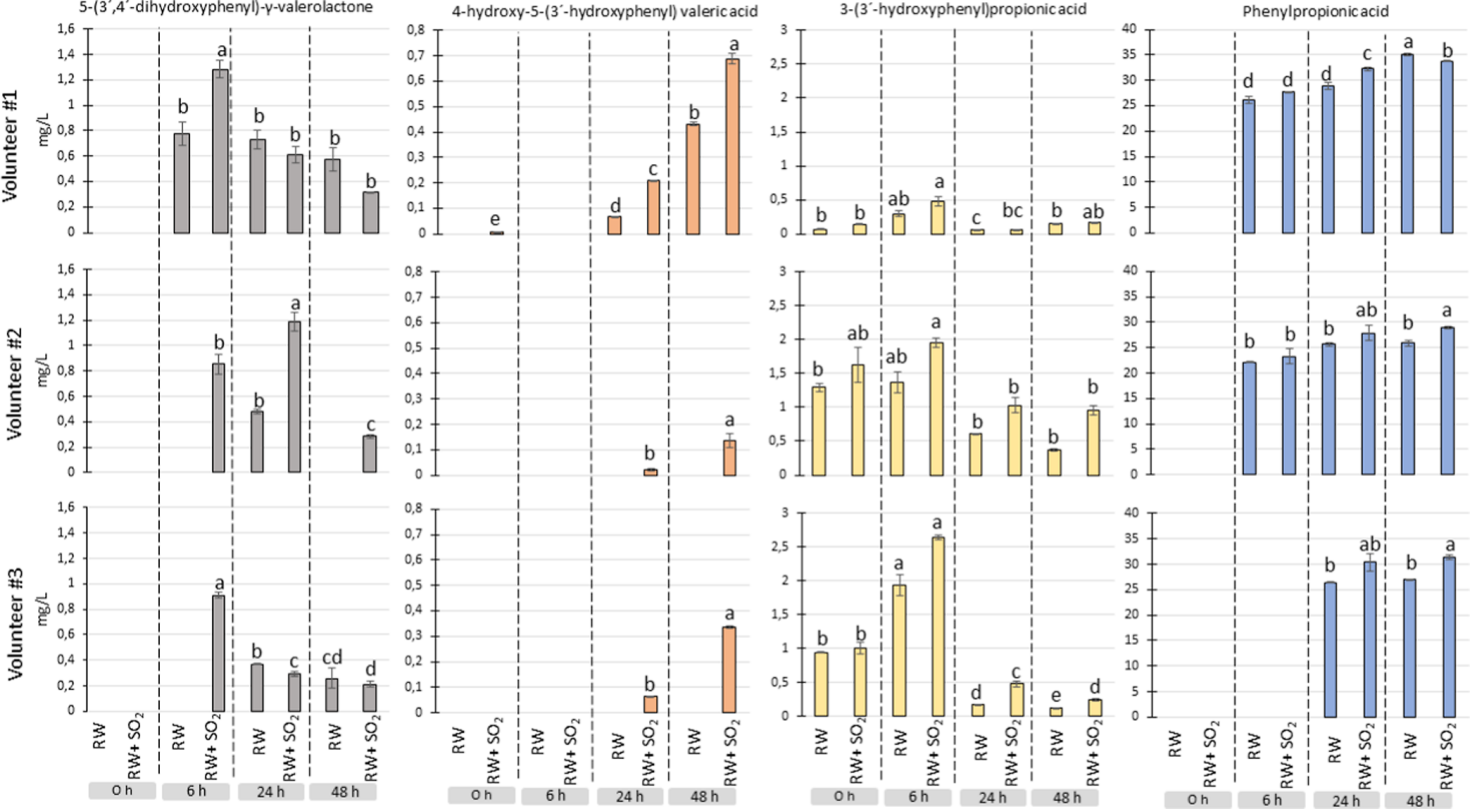
Evolution of concentration of phenolic metabolites during colonic fermentation at different times (0, 6, 24, and 48 h) for the four studied wines (SW, SW + SO2, RW, and RW + SO2) and for each volunteer (#1, #2, and #3). Lowercase letters denote significant differences between treatments within each phenolic metabolite.

## 4 Discussion

In food systems, sulfur dioxide (SO_2_) exhibits a broad spectrum of antimicrobial activity, which is based on its capacity to deteriorate cellular membrane integrity, the leakage of intracellular metabolites, the change in intracellular pH, and the depletion of oxygen in microbial cells, among other mechanisms.^44^ However, one of the fields that have remained practically unexplored until now is the effect of sulfites on gut microbiota, which is considered a currently active research target in relation to food and health. To go deeper into this knowledge gap, and in light of the widespread use of sulfites in wine, we designed an in vitro gastrointestinal-simulation experiment that included red and synthetic wines, untreated and treated with SO_2_, in permitted concentrations. As a result, the study allowed us to investigate not only the impact of sulfites on gut microbiota by themselves (using synthetic wine) but also the influence on the latter of the whole wine matrix, polyphenols in particular (using red wine).

Previous characterization of the wines used revealed the ability of red wine components to be combined with SO_2_, a matter that has been described at length in the literature. It is well-known that, once added to wine, a portion of the bisulfite form (HSO_3_^–^) is able to bind wine components such as polyphenols (anthocyanins in particular) and carbonyl compounds (e.g., acetaldehyde), ketoacids, and sugars, thereby decreasing the levels of the free form.^45^ This sulfite reactivity with wine components was evidenced in our results since part of the SO_2_ was found as a combined form in the SO_2_-treated red wine (RW + SO2) but not in the SO_2_-treated synthetic wine (SW + SO2). Moreover, as acetaldehyde is able to react rapidly with SO_2_ even at low temperatures,^46^ we hypothesized (and later confirmed) that this combined SO_2_ in RW + SO2 was done, at least partly, with acetaldehyde, leading to a lower acetaldehyde level in the SO_2_-treated wine. With regard to polyphenols, reactions with SO_2_ are known to allow a dissociation of their adducts with proteins and polysaccharides, and also to prevent oxidation of wine polyphenols, especially anthocyanins as more oxidation-susceptible wine phenolic compounds.^47^ This could explain the slightly higher concentration of polyphenols, particularly anthocyanins, determined in the SO_2_-treated red wine than in the untreated wine. The exception to this general trend was gallic acid, whose content was lower in the SO_2_-treated red wine, and that was explained by the particular oxidation of this acid by sulfate radical anions.^48^ Therefore, it is conceivable that the impact of polyphenols and sulfites on the gut microbiota would be mediated, at least in part, by their dynamic interactions, including the formation of sulfonated adducts.

Microbial counting and metataxonomic analysis of gut microbiota after fermentation with gastrointestinal-digested wines revealed a noticeable impact of wine sulfites on microbiota composition based on the simgi model, an impact that was greatly influenced by individual variability. Taxonomically, the Proteobacteria phylum seemed to be stimulated by the presence of sulfites, as evidenced by the synthetic wine. This phylum, which includes a great variety of pathogens, has been related to metabolic disorders and inflammatory bowel disease, an assumption that should be treated with caution.^49^ Results comparing SO_2_-treated and untreated synthetic wines also showed that certain genera might be affected by wine sulfites as significant population shifts were detected for at least one particular gut microbiota. Thus, decreases in the populations of beneficial genera like *Blautia*, *Ruminococcus*, *Butyricicoccus*, and *Bacteroides* as well as increases in *Coprococcus*, *Escherichia*/*Shigella*, and *Parasutterella* levels were observed in some cases. In line with these findings, in a previous study of batch-culture fermentations of different food additives with fecal microbiota, the Shannon α-diversity index was affected, and the addition of sodium sulfite increased the abundance of *Escherichia*/*Shigella* and *Bilophila* and had an inhibitory effect on the growth of *Bifidobacterium*.^50^ Similarly, in a mouse model, increases in the populations of *Bacteroides*, *Blautia*, *Ruminococcus*, *Oscillospira*, and *Dorea* were found after feeding with benzoate, along with a greater abundance of *Firmicutes*, *Turicibacter*, and *Alkaliphilus* following sodium nitrate consumption, and an increased proportion of Parabacteroides and *Adlercreutzia* after consuming potassium sorbate at recommended doses.^51^

At species level, SO_2_ treatment in synthetic wine, and for at least one particular gut microbiota, appeared to decrease the relative abundance of some bacteria related to positive health effects and reported to have critical effects on the ecology and balance of gut microbial communities,^52^ such as *Bifidobacterium longum*, *P. distasonis*, and *B. obeum*.^53,54^ On the other hand, SO_2_-treated synthetic wine led to a higher relative abundance of some taxa related to negative health effects such as *Escherichia*/*Shigella* ^55^ and *A. putredinis*,^56^ as well as *V. parvula*, all of them exhibiting pro-inflammatory potential and negative health effects.^57,58^ Therefore, wine sulfites seemed to affect selected minority taxa, with individual particularities of gut microbiome conditioning its resilience toward SO_2_. This was in line with finding almost no differences in microbial diversity (Shannon and Simpson indexes) between SO_2_-treated and untreated wines for each volunteer. In the same way, in terms of microbial functionality, SO_2_ treatment in wines appeared to slightly condition all fermentative (SCFA production) and proteolytic (ammonium production) gut microbiota activities, with the extent of the conditioning being dependent on individual microbiota.

Additionally, we investigated potential changes in sulfite-reducing bacteria. Although these bacteria are generally considered to be a minor component of the human gut microbiota, they may play a role in certain disease contexts. We focused not only in the phylum *Thermodesulfobacteriota*, which includes most described sulfite-reducing bacteria, such as the genera *Desulfobacter*, *Desulfobacterium*, *Desulfovibrio*, and *Desulfotomaculum* ^59^ but also on other microbial groups capable of reducing sulfite to sulfide, collectively referred to as sulfidogenic bacteria. Among these, *Bilophila*, particularly *Bilophila wadsworthia*, is one of the best characterized,^57^ and was indeed detected in our study. However, no significant differences were observed in the relative abundance of any of these groups between SO_2_-treated and untreated wine conditions across the three donors (results not shown).

Another remarkable finding from this study was that all these SO_2_-induced changes in microbial viability, taxonomy, and metabolic activity were observed to a lesser extent in red wine, which suggested that certain components of the red wine matrix may play a putative gut protective role. This could be due to the ability of red wine components, mainly polyphenols and fiber, to promote the growth of commensals and taxa related to positive health effects such as *Bifidobacterium*, *Blautia*, and *Bacteroides* members,^28–30^ and to limit the growth of, or exert an inhibitory effect against, pathogens such as *Escherichia*/*Shigella* or pro-inflammatory bacteria such as *B. wadsworthia*, all of which could buffer or counteract SO_2_-negative effects on microbial communities. On the other hand, wine polyphenols and/or their metabolites could act as protectors against the potential negative effects of SO_2_ on microbial communities through their direct interaction with SO_2_, lowering its levels in free reactive form.^60,61^ Further research on these interactions involving SO_2_, wine components (i.e., polyphenols), and gut microbiome, including monitoring free and bound SO_2_, is worthwhile.

The results of this study also complement those obtained by other authors on the effect of sulfites on the human microbiota in general. Irwin and collaborators^62^ previously demonstrated the bacteriostatic effect of sulfites (NaHSO_3_ and Na_2_SO_3_) against four bacteria related to positive health effects belonging to the *Streptococcus* and *Lactobacillus* genera. However, in our study, a limited effect of SO_2_ was observed in plate-counting results. The greater complexity of the colonic environment, which seems to favor combined forms of SO_2_ characterized by weak antimicrobial activity,^9^ could be responsible for the observed effects. On the other hand, SO_2_ was also shown to contribute to a decrease in the viability of *Proteus mirabilis*, *Escherichia fergusonii*, *Morganella morganii*, and *Klebsiella pneumoniae* isolated from patients with Crohn’s disease.^63^ However, it should be noted that the previous studies were performed on cultures and directly added a concentration of SO_2_ similar to that allowed in food, therefore without considering the changes that occur during the digestion process, unlike the present work. Irwin et al.^23^ evaluated the effects of sulfite preservatives on the oral microbiome. In the specific case of sodium bisulfite (NaHSO_3_), a higher alpha-diversity when compared to the control samples and a dissimilarity in microbial structure profiles within the samples were observed. These findings differ greatly from those obtained in our study, which could be due to differences between the oral and intestinal microbial communities. Furthermore, it should be taken into consideration that, while in Irwin’s study a direct exposure of sulfites was carried out for a short time (30 min), our study aimed to go a step further by trying to evaluate, within a complex food matrix such as wine subjected to the physiological process of digestion and colonic fermentation, the effects of SO_2_ on the intestinal microbiota.

In addition, this study provides new data on the colonic metabolism of wine polyphenols. After the gastrointestinal stage, most polyphenols reach the colon intact, where they undergo a marked microbial metabolism, which conditions their biological effects.^64^ Therefore, taking advantage of the modular character of simgi, the gastrointestinal digests were used to carry out colonic fermentation assays with SO_2_-treated wines. The simgi modular system has been successfully applied in previous codigestion studies with wine and beer.^29,30,65^ As expected, during colonic fermentation, the degradation of wine polyphenols led to the appearance of microbial phenolic metabolites (phenyl-γ-valerolactones phenylvaleric acids, phenylacetic acids, phenylpropionic acids, and benzoic acids) as seen in previous studies.^28^ The sequence of metabolite generation observed from 0 to 6, 24, and 48 h [first, 5-(3′,4′-dihydroxyphenyl)-γ-valerolactone, and then, 5-(3′-hydroxyphenyl)-γ-valerolactone, and later their corresponding valeric acids] was in accordance with the described pathway for the catabolism of wine flavan-3-ols.^66^ The first catabolic steps give rise to the intermediate 1-(3′,4′-dihydroxyphenyl)-3-(2″,4″,6″-trihydroxyphenyl)-propan-2-ol (this compound was not detected in our study), which is subsequently converted into 5-(3′,4′-dihydroxyphenyl)-γ-valerolactone and 4-hydroxy-5-(3′,4′-dihydroxyphenyl)-valeric acid, which are further dehydroxylated into the 3′-hydroxyphenyl forms. In our case, only the derivate 4-hydroxy-5-(3′-hydroxyphenyl)-valeric acid was detected. Later, the microbial metabolism of these intermediate metabolites leads to the formation of phenylpropionic and phenylacetic acids, as seen in our study. Moreover, the higher concentration of low-molecular-weight phenolic compounds bioavailable both at small intestinal and colonic level could be attributed to the higher content of phenolic precursors in the wine before digestion and/or an activation of phenolic metabolism at the intestinal level as a consequence of SO_2_ treatment. We cannot ignore the fact either that the above-described slight modulating effect of SO_2_ on the abundance of selected-minority taxa/microbial subpopulations in the intestinal microbiota impacts the metabolism of wine polyphenols.

Overall, these results support the potential effects of SO_2_ on the composition and structure of the human colonic microbiota by slightly increasing the levels of minor taxa with sulfite-reducing, bile-tolerant, and protein-degrading activities and to the detriment of commensals or species related to positive health effects. This SO_2_ impact on gut microbiota was found to be attenuated, at least partly, in red wine, which suggested a protective action of wine components (polyphenols) against sulfite-induced microbial alterations. Although this mechanism remains speculative, the presence of residual combined SO_2_ after gastrointestinal digestion suggests possible interactions between sulfites and wine matrix components—potentially including polyphenols. Further studies are needed to confirm the chemical identity of these adducts and their functional relevance. In terms of microbial functionality, SO_2_ treatment in wines appeared not to greatly impact the fermentative (SCFA production) and proteolytic (ammonium production) activities of gut microbiota. It should be noted that the relatively low number of fecal donors (*n* = 3) may limit the generalizability of the results. However, it is important to emphasize that the present study was conceived as a first exploratory investigation, aimed at evaluating the potential impact of wine sulfites, within regulatory limits, on human gut microbiota activity. In any case, the impact of SO_2_ on gut microbiota is found to be dependent on individual variability, which opens new research lines to ascertain what the individual particularities of the gut microbiome are that may affect its resilience toward SO_2_.

The observed effects may be clinically relevant for individuals sensitive to sulfites, particularly those experiencing intolerances or allergies. For instance, the association of *Escherichia*/*Shigella* with inflammatory mechanisms in IgE-mediated food allergies highlights the need for further investigation into these links through clinical studies.^67^ In this context, the increase in *Escherichia*/*Shigella* observed under SO_2_-treated conditions is suggestive, as members of this genus have been associated with adverse immune responses in previous studies.^68^ However, it is essential to note that this work was conducted in an in vitro model. Although dynamic simulators like simgi offer high reproducibility and experimental control, the findings must be validated in vivo to fully understand their physiological implications. This study opens new research avenues on the interaction between sulfites, food matrices, and gut microbiota. Future studies with could explore the specific mechanisms by which polyphenols counteract the adverse effects of sulfites and identify the individual microbiome characteristics that influence resilience to these compounds. Longitudinal human studies could further clarify the long-term impacts of sulfite consumption on gut health.

## Supplementary Material



## Notes

Table S1. Alpha-diversity analysis of gut microbiota in terms of Observed ASVs, Shannon and Simpson indexes during fermentations of gastrointestinal-digested wines with faecal microbiota from three different volunteers (#1, #2, and #3). Statistically significant differences, assessed by two-way ANOVA test and Games-Howell correction (*p* < 0.05), are marked by lowercase letters. Shaded cells indicate significant differences between SO_2_-treated wine and its untreated counterpart. Figure S1. Nonmetric multidimensional scaling (NMDS) representation of the microbial beta-diversity from the colonic fermentation samples at 0, 24, and 48 h for the four studied wines (SW, SW + SO2, RW, RW + SO2) and for each volunteer (#1, #2, and #3). Table S2. Taxa at phylum level during colonic fermentation at different times (0, 24, and 48 h) for the four studied wines (SW, SW + SO2, RW, RW + SO2) and for each volunteer (#1, #2, and #3). Data are expressed as mean relative abundance (%) ± standard deviation. Only phyla with a mean relative abundance >0.1% in at least one case are showed. Statistically significant differences, assessed by two-way ANOVA test and Games-Howell correction (*p* < 0.05), are marked by lowercase letters. Shaded cells indicate significant differences between SO_2_-treated wine and its untreated counterpart. Table S3. Taxa at genus level during colonic fermentation at different times (0, 24, and 48 h) for the four studied wines (SW, SW + SO2, RW, and RW + SO2) and for each volunteer (#1, #2, and #3). Data are expressed as mean relative abundance (%) ± standard deviation. Only genera with a mean relative abundance >0.4% in at least one case are showed. Statistically significant differences, assessed by two-way ANOVA test and Games–Howell correction (*p* < 0.05), are marked by lowercase letters. Table S4. Taxa at species level during colonic fermentation at different times (0, 24, and 48 h) for the four studied wines (SW, SW + SO2, RW, and RW + SO2) and for each volunteer (#1, #2, and #3). Data are expressed as mean relative abundance (%) ± standard deviation. Only species properly assigned to a species present in taxonomic databases and showing statistical differences in at least one case are showed. Statistically significant differences, assessed by two-way ANOVA test and Games–Howell correction (*p* < 0.05), are marked by lowercase letters. Shaded cells indicate significant differences between SO_2_-treated wine and its untreated counterpart. Table S5. Concentration (μM) of short-chain fatty acids (SCFAs) during colonic fermentation at different times (0, 24, and 48 h) for the four studied wines (SW, SW + SO2, RW, and RW + SO2) and for each volunteer (#1, #2, and #3). Statistically significant differences, assessed by two-way ANOVA test and Games-Howell correction (*p* < 0.05), are marked by lowercase letters. Shaded cells indicate significant differences between SO_2_-treated wine and its untreated counterpart. Table S6. Concentration (μM) of ammonium (NH_4_^+^) during colonic fermentation at different times (0, 24, and 48 h) for the four studied wines (SW, SW + SO2, RW, and RW + SO2) and for each volunteer (#1, #2, and #3). Statistically significant differences, assessed by two-way ANOVA test with Tukey post hoc correction (*p* < 0.05), are marked by lowercase letters. Shaded cells indicate significant differences between SO_2_-treated wine and its untreated counterpart. Table S7. Concentration of phenolic compounds in red wines (RW and RW + SO2) and their corresponding gastrointestinal digests. Data are expressed as mean values (*n* = 3) ± standard deviation. The Kruskal–Wallis test was used to test for significant differences. Table S8. Concentration of wine phenolic metabolites during colonic fermentation at different times (0, 6, 24, and 48 h) for the red wines (RW and RW + SO2) and for each volunteer (#1, #2, and #3). Data are expressed as mean values (*n* = 3) ± standard deviation. Statistically significant differences, assessed by two-way ANOVA test with Tukey post hoc correction (*p* < 0.05), are marked by lowercase letters. Shaded cells indicate significant differences between SO2-treated wine and its untreated counterpart (PDF)
