# Supplementary material for: May Sulfites in Wine Affect Gut Microbiota? An In Vitro Study of Their Digestion and Interplay with Wine Polyphenols
Source: J Agric Food Chem. 2025 Jul 23;73(31):19397–409. doi: 10.1021/acs.jafc.5c02710 (PMC12510145; doi:10.1021/acs.jafc.5c02710)
Supplement: Supplementary file 1 [file jf5c02710_si_001.pdf]

SUPPORTING INFORMATION

May sulfites in wine affect gut microbiota? An *in vitro* study of their digestion and interplay with wine polyphenols

Edgard Relaño de la Guía[1], Carolina Cueva[1], Natalia Molinero[1], Ana Ruano[2], M.José Motilva[3], Begoña Bartolomé[1], M. Victoria Moreno-Arribas[1,*]

[1] Institute of Food Science Research (CIAL), CSIC-UAM, c/ Nicolás Cabrera 9. 28049, Madrid, Spain

[2]Agri-food Arbitration Laboratory. S. G. Food Quality Control and Agri-Food Laboratories. D.G. of Food. Ministry of Agriculture, Fisheries and Food. Madrid, Spain

[3]Institute of Grapevine and Wine Sciences (ICVV), CSIC-University of La Rioja-Government of La Rioja, 26007 Logroño (La Rioja), Spain

* Corresponding author: victoria.moreno@csic.es (M. V. Moreno-Arribas)

Table S1. Alpha-diversity analysis of gut microbiota in terms of Observed ASVs, Shannon and Simpson indexes during fermentations of gastrointestinal-digested wines with faecal microbiota from three different volunteers (#1, #2, and #3). Statistically significant differences, assessed by two-way ANOVA test and Games-Howell correction ($p < 0.05$), are marked by lowercase letters. Shaded cells indicate significant differences between SO_2-treated wine and its untreated counterpart, and arrows (↑/↓) indicate the direction of change.

VOLUNTEER # 1												
	0 h				24 h				48 h			
Diversity	SW	SW + SO2	RW	RW + SO2	SW	SW + SO2	RW	RW + SO2	SW	SW + SO2	RW	RW + SO2
Observed	234.33 ± 6.43 ^{a}	242.33 ± 11.72 ^{a}	239.67 ± 14.22 ^{a}	226.00 ± 9.54 ^{a}	236.67 ± 19.35 ^{a}	228.67 ± 6.51 ^{a}	231.67 ± 8.08 ^{a}	220.67 ± 23.63 ^{a}	234.00 ± 5.00 ^{a}	205.00 ± 2.65 ^{a}	245.33 ± 10.97 ^{a}	230.33 ± 7.02 ^{a}
Shannon	4.26 ± 0.05 ^{a}	4.27 ± 0.06 ^{a}	4.27 ± 0.04 ^{a}	4.24 ± 0.02 ^{a}	4.18 ± 0.02 ^{a}	4.17 ± 0.04 ^{a}	4.16 ± 0.01 ^{a}	4.19 ± 0.02 ^{a}	4.09 ± 0.1 ^{ab}	3.79 ± 0.06 ^{b}	3.92 ± 0.04 ^{b}	3.97 ± 0.04 ^{b}
Simpson	0.97 ± 0.01 ^{a}	0.97 ± 0.01 ^{a}	0.97 ± 0.01 ^{a}	0.97 ± 0.01 ^{a}	0.96 ± 0.01 ^{a}	0.96 ± 0.01 ^{a}	0.97 ± 0.01 ^{a}	0.97 ± 0.01 ^{a}	0.96 ± 0.01 ^{ab}	0.95 ± 0.01 ^{b}	0.95 ± 0.01 ^{b}	0.95 ± 0.01 ^{b}
VOLUNTEER # 2												
	0 h				24 h				48 h			
Diversity	SW	SW + SO2	RW	RW + SO2	SW	SW + SO2	RW	RW + SO2	SW	SW + SO2	RW	RW + SO2
Observed	212.00 ± 12.77 ^{a}	217.33 ± 7.51 ^{a}	208.33 ± 17.21 ^{a}	215.00 ± 1.00 ^{a}	193.67 ± 26.84 ^{a}	184.33 ± 6.66 ^{a}	205.33 ± 8.50 ^{a}	207.00 ± 4.36 ^{a}	212.67 ± 3.79 ^{a}	184.00 ± 12.17 ^{a}	205.00 ± 13.23 ^{a}	201.67 ± 10.60 ^{a}
Shannon	4.03 ± 0.05 ^{ab}	4.03 ± 0.03 ^{a}	4.04 ± 0.02 ^{a}	4.05 ± 0.01 ^{a}	3.81 ± 0.01 ^{b}	3.71 ± 0.08 ^{bc}	3.86 ± 0.05 ^{ab}	3.82 ± 0.06 ^{ab}	3.71 ± 0.13 ^{bc}	3.69 ± 0.02 ^{c}	3.68 ± 0.09 ^{c}	3.66 ± 0.09 ^{c}
Simpson	0.96 ± 0.01 ^{a}	0.96 ± 0.01 ^{a}	0.96 ± 0.01 ^{a}	0.96 ± 0.01 a	0.95 ± 0.00 ^{b}	0.94 ± 0.01 ^{b}	0.95 ± 0.01^{ab}	0.95 ± 0.01 ^{ab}	0.94 ± 0.01 ^{b}	0.95 ± 0.04 ^{b}	0.93 ± 0.01 ^{b}	0.94 ± 0.01 ^{b}
VOLUNTEER # 3												
	0 h				24 h				48 h			
Diversity	SW	SW + SO2	RW	RW + SO2	SW	SW + SO2	RW	RW + SO2	SW	SW + SO2	RW	RW + SO2
Observed	255.00 ± 7.81 ^{b}	257.67 ± 17.62 ^{b}	285.33 ± 16.01 ^{b}	239.67 ± 4.04 ^{b}	247.00 ± 8.89 ^{b}	240.00 ± 7.94 ^{b}	277.67 ± 2.89 ^{b}	281.67 ± 29.74 ^{b}	298.33 ± 13.61 ^{ab}	296.00 ± 19.00 ^{b}	296.33 ± 7.51 ^{b}	339.00 ± 2.00 ^{a} (↑)
Shannon	4.39 ± 0.01 ^{abc}	4.43 ± 0.01 ^{ab}	4.44 ± 0.02 ^{a}	4.38 ± 0.02 ^{abc}	4.26 ± 0.01 ^{de}	4.17 ± 0.04 ^{e}	4.35 ± 0.02 ^{abcd}	4.35 ± 0.03 ^{abcd}	4.18 ± 0.03 ^{e}	4.14 ± 0.05 ^{e}	4.27 ± 0.03 ^{bcde}	4.27 ± 0.03 ^{cde}
Simpson	0.97 ± 0.01 ^{abc}	0.98 ± 0.01 ^{a}	0.98 ± 0.01 ^{ab}	0.98 ± 0.01 ^{abc}	0.97 ± 0.01 ^{bc}	0.97 ± 0.01 ^{cd}	0.97 ± 0.01 ^{bc}	0.97 ± 0.01 ^{bc}	0.96 ± 0.01 ^{d}	0.96 ± 0.01 ^{d}	0.96 ± 0.01 ^{cd}	0.96 ± 0.01 ^{d}

nd: not detected, SW: synthetic wine, SW+SO2: synthetic wine treated with SO_2, RW: red wine, RW+SO2: red wine treated with SO_2

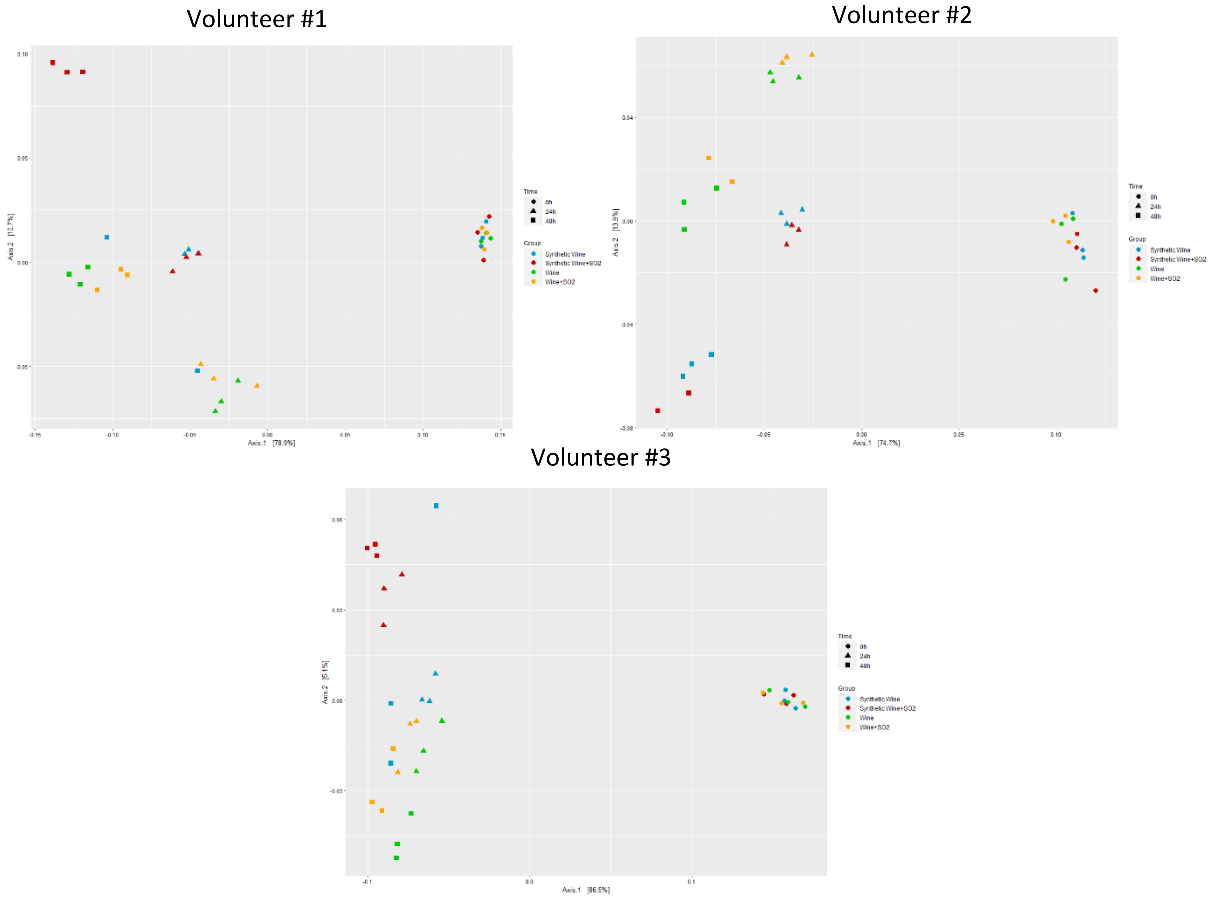

Figure S1. Non-metric multidimensional scaling (NMDS) representation of the microbial beta-diversity from the colonic fermentation samples at 0, 24 and 48 h for the four studied wines (SW, SW+SO2, RW, RW+SO2) and for each volunteer (#1, #2, and #3).

Table S2. Taxa at phylum level during colonic fermentation at different times (0, 24, and 48 hours) for the four studied wines (SW, SW+SO2, RW, RW+SO2) and for each volunteer (#1, #2, and #3). Data are expressed as mean relative abundance (%) ± standard deviation. Only phyla with a mean relative abundance >0.1% in at least one case are showed. Statistically significant differences, assessed by two-way ANOVA test and Games-Howell correction ($p < 0.05$), are marked by lowercase letters. Shaded cells indicate significant differences between SO_2-treated wine and its untreated counterpart, and arrows (↑/↓) indicate the direction of change.

VOLUNTEER # 1												
	0 h				24 h				48 h			
Phylum	SW	SW + SO2	RW	RW + SO2	SW	SW + SO2	RW	RW + SO2	SW	SW + SO2	RW	RW + SO2
Actinobacteriota	10.82 ± 0.44^{c}	10.53 ± 0.33^{c}	10.64 ± 0.54^{c}	10.84 ± 0.38^{c}	25.3 ± 0.49^{bc}	23.91 ± 1.02^{c}	21.38 ± 0.75^{c}	21.06 ± 3.27^{c}	29.07 ± 5.36^{abc}	22.22 ± 0.49^{c}	34.46 ± 0.98^{a}	30.65 ± 1.52^{ab}
Firmicutes	69.06 ± 1.57^{a}	69.12 ± 2.05^{a}	68.56 ± 0.38^{a}	68.31 ± 0.77^{a}	54.33 ± 0.15^{b}	53.03 ± 2.11^{bc}	50.57 ± 2.00^{bc}	52.4 ± 3.28^{bc}	47.92 ± 1.32^{bc}	37.45 ± 1.04^{d} (↓)	44.59 ± 1.17^{c}	49.49 ± 2.35^{bc}
Desulfobacterota	0.16 ± 0.03^{c}	0.15 ± 0.02^{c}	0.15 ± 0.02^{c}	0.16 ± 0.01^{c}	5.55 ± 0.46^{bc}	6.38 ± 0.34^{b}	3.95 ± 0.31^{c}	4.41 ± 0.3^{c}	6.02 ± 1.55^{bc}	6.61 ± 0.18^{ab}	8.9 ± 0.51^{a}	8.03 ± 0.27^{a}
Proteobacteria	0.51 ± 0.04^{c}	0.58 ± 0.12^{c}	0.52 ± 0.06^{c}	0.57 ± 0.02^{c}	4.02 ± 0.21^{b}	4.29 ± 0.32^{b}	2.83 ± 0.06^{b}	3.45 ± 0.47^{b}	4.5 ± 0.43^{b}	26.13 ± 1.68^{a} (↑)	4.26 ± 0.19^{b}	3.63 ± 0.16^{b}
Bacteroidota	19.34 ± 1.17^{a}	19.46 ± 1.87^{a}	19.99 ± 0.14^{a}	20.02 ± 0.71^{a}	10.39 ± 0.35^{b}	12.03 ± 0.63^{b}	21.02 ± 1.50^{a}	18.42 ± 0.78^{a}	11.97 ± 6.25^{b}	7.12 ± 0.59^{c} (↓)	7.08 ± 0.08^{c}	7.84 ± 1.10^{bc}
Fusobacteriota	nd	nd	nd	nd	0.11 ± 0.01^{a}	0.05 ± 0.01^{a}	0.03 ± 0.01^{a}	0.03 ± 0.03^{a}	0.16 ± 0.11^{a}	0.05 ± 0.01^{a}	0.14 ± 0.01^{a}	0.06 ± 0.01^{a}
VOLUNTEER # 2												
	0 h				24 h				48 h			
Phylum	SW	SW + SO2	RW	RW + SO2	SW	SW + SO2	RW	RW + SO2	SW	SW + SO2	RW	RW + SO2
Actinobacteriota	2.05 ± 0.06^{de}	2.29 ± 0.35^{de}	2.36 ± 0.28^{de}	2.02 ± 0.13^{e}	2.49 ± 0.06^{de}	3.23 ± 0.12^{c} (↑)	4.41 ± 0.22^{b}	4.16 ± 0.16^{b}	3.15 ± 0.23^{cd}	4.28 ± 0.13^{b} (↑)	5.86 ± 0.17^{a}	5.50 ± 0.33^{ab}
Firmicutes	70.47 ± 2.49^{a}	69.68 ± 2.86^{a}	70.52 ± 3.35^{a}	68.94 ± 2.65^{a}	54.73 ± 0.38^{a}	49.75 ± 0.55^{b} (↓)	57.33 ± 0.34^{a}	57.01 ± 0.90^{a}	54.44 ± 6.10^{ab}	48.3 ± 0.90^{b}	59.26 ± 1.12^{a}	55.93 ± 1.97^{a}
Desulfobacterota	0.14 ± 0.02^{d}	0.15 ± 0.01^{d}	0.13 ± 0.02^{d}	0.13 ± 0.02^{d}	4.96 ± 0.43^{b}	5.07 ± 0.42^{b}	2.81 ± 0.23^{c}	3.02 ± 0.11^{bc}	8.04 ± 0.32^{a}	8.46 ± 0.38^{a}	4.44 ± 0.26^{b}	5.06 ± 0.33^{b}
Proteobacteria	5.13 ± 1.13^{e}	5.20 ± 0.59^{e}	4.97 ± 0.53^{e}	5.21 ± 0.76^{e}	19.37 ± 0.74^{bc}	24.26 ± 0.75^{a} (↑)	17.11 ± 0.26^{c}	18.23 ± 0.49^{c}	18.58 ± 3.3^{bc}	22.65 ± 0.36^{ab}	13.1 ± 0.23^{d}	15.96 ± 1.33^{cd}
Bacteroidota	22.1 ± 1.35^{a}	22.59 ± 2.41^{a}	21.93 ± 2.51^{a}	23.63 ± 1.88^{a}	18.33 ± 0.33^{a}	17.45 ± 0.85^{a}	18.19 ± 0.81^{a}	17.42 ± 0.53^{a}	15.67 ± 2.48^{a}	16.08 ± 0.97^{a}	17.25 ± 1.20^{a}	17.43 ± 1.16^{a}
VOLUNTEER # 3												
	0 h				24 h				48 h			
Phylum	SW	SW + SO2	RW	RW + SO2	SW	SW + SO2	RW	RW + SO2	SW	SW + SO2	RW	RW + SO2
Actinobacteriota	8.34 ± 0.44^{d}	8.47 ± 0.31^{d}	9.14 ± 1.12^{d}	9.26 ± 0.44^{d}	16.39 ± 1.17^{bc}	15.23 ± 0.96^{c}	17.03 ± 1.09^{bc}	17.62 ± 0.86^{bc}	23.08 ± 1.11^{a}	22.49 ± 1.57^{ab}	24.41 ± 2.00^{a}	24.93 ± 1.29^{a}
Firmicutes	54.72 ± 1.25^{a}	57.39 ± 2.59^{a}	54.56 ± 2.20^{ab}	53.7 ± 0.36^{abc}	46.62 ± 0.67^{abcd}	48.13 ± 1.13^{abc}	47.44 ± 1.83^{abc}	48.6 ± 2.16^{abcd}	42.89 ± 0.92^{d}	45.68 ± 1.66^{bcd}	44.31 ± 2.09^{cd}	43.47 ± 2.14^{cd}
Desulfobacterota	0.04 ± 0.03^{d}	0.08 ± 0.02^{d}	0.04 ± 0.01^{d}	0.06 ± 0.01^{d}	3.79 ± 0.02^{b}	3.45 ± 0.31^{bc}	2.12 ± 0.16^{c}	2.13 ± 0.1^{c}	6.72 ± 0.38^{a}	6.43 ± 0.51^{a}	3.8 ± 0.22^{b}	3.72 ± 0.1^{b}
Proteobacteria	11.1 ± 0.50^{a}	9.3 ± 0.86^{ab}	10.29 ± 1.40^{ab}	10.44 ± 1.10^{ab}	7.95 ± 0.22^{b}	11.17 ± 0.38^{a} (↑)	5.69 ± 0.35^{b}	5.85 ± 0.35^{b}	8.24 ± 0.20^{b}	11.17 ± 0.03^{a} (↑)	6.33 ± 0.43^{b}	5.57 ± 0.17^{b}
Bacteroidota	25.71 ± 1.41^{ab}	24.7 ± 1.70^{ab}	25.83 ± 1.86^{ab}	26.47 ± 0.52^{a}	25.14 ± 0.98^{ab}	21.89 ± 1.11^{b}	27.59 ± 0.85^{ab}	25.67 ± 0.82^{a}	18.92 ± 1.07^{bc}	14.05 ± 0.44^{c}	21.01 ± 1.29^{b}	22.14 ± 0.89^{b}

nd: not detected, SW: synthetic wine, SW+SO2: synthetic wine treated with SO_2, RW: red wine, RW+SO2: red wine treated with SO_2

Table S3. Taxa at genus level during colonic fermentation at different times (0, 24, and 48 hours) for the four studied wines (SW, SW+SO2, RW, and RW+SO2) and for each volunteer (#1, #2, and #3). Data are expressed as mean relative abundance (%) ± standard deviation. Only genera with a mean relative abundance >0.4 % in at least one case are showed. Statistically significant differences, assessed by two-way ANOVA test and Games-Howell correction ($p < 0.05$), are marked by lowercase letters.

VOLUNTEER # 1

	0h				24h				48h			
Genus	SW	SW + SO2	RW	RW + SO2	SW	SW + SO2	RW	RW + SO2	SW	SW + SO2	RW	RW + SO2
Collinsella	3.75 ± 0.31 ^{b}	3.55 ± 0.17 ^{b}	3.45 ± 0.20 ^{b}	3.66 ± 0.17 ^{b}	10.61 ± 0.44 ^{b}	9.57 ± 1.02 ^{b}	9.00 ± 0.07 ^{b}	8.53 ± 1.83 ^{b}	11.41 ± 1.51 ^{ab}	9.14 ± 0.54 ^{b}	14.35 ± 0.70 ^{a}	11.66 ± 2.30 ^{ab}
Bifidobacterium	5.89 ± 0.17 ^{c}	5.82 ± 0.10 ^{c}	5.96 ± 0.40 ^{c}	6.05 ± 0.53 ^{c}	11.37 ± 0.41 ^{bc}	11.31 ± 0.32 ^{bc}	10.18 ± 0.48 ^{c}	10.23 ± 0.94 ^{c}	13.82 ± 2.58 ^{abc}	10.13 ± 0.39 ^{c}	16.55 ± 0.43 ^{a}	15.52 ± 1.13 ^{ab}
Bilophila	0.14 ± 0.03 ^{c}	0.13 ± 0.01 ^{c}	0.11 ± 0.02 ^{c}	0.13 ± 0.01 ^{c}	5.52 ± 0.46 ^{bc}	6.36 ± 0.35 ^{b}	3.93 ± 0.32 ^{c}	4.39 ± 0.30 ^{c}	5.97 ± 1.53 ^{bc}	6.6 ± 0.18 ^{ab}	8.89 ± 0.51 ^{a}	8 ± 0.27 ^{a}
Agathobacter	6.14 ± 0.41 ^{a}	6.11 ± 0.18 ^{a}	6.79 ± 0.27 ^{a}	6.35 ± 0.11 ^{a}	1.17 ± 0.17 ^{b}	0.90 ± 0.04 ^{b}	0.68 ± 0.13 ^{b}	0.64 ± 0.09 ^{b}	0.41 ± 0.08 ^{bc}	0.18 ± 0.07 ^{c}	0.39 ± 0.06 ^{bc}	0.21 ± 0.02 ^{bc}
Escherichia/Shigella	nd	nd	0.02 ± 0.01 ^{c}	0.02 ± 0.01 ^{c}	0.45 ± 0.03 ^{a}	0.12 ± 0.02 ^{b}	nd	0.02 ± 0.01 ^{bc}	0.07 ± 0.03 ^{bc}	0.01 ± 0.02 ^{c}	0.02 ± 0.01 ^{bc}	0.04 ± 0.01 ^{bc}
Phascolarctobacterium	2.01 ± 0.15 ^{a}	2.08 ± 0.17 ^{a}	1.68 ± 0.12 ^{a}	1.59 ± 0.18 ^{a}	2.57 ± 0.25 ^{a}	2.15 ± 0.08 ^{a}	2.29 ± 0.08 ^{a}	2.18 ± 0.26 ^{a}	2.29 ± 0.01 ^{a}	1.14 ± 0.03 ^{a}	1.96 ± 0.12 ^{a}	2.11 ± 0.13 ^{a}
Faecalibacterium	13.01 ± 1.37 ^{a}	13.31 ± 1.70 ^{a}	12.62 ± 1.05 ^{a}	12.85 ± 0.97 ^{a}	2.21 ± 0.22 ^{b}	1.9 ± 0.13 ^{b}	1.78 ± 0.40 ^{bc}	1.83 ± 0.03 ^{b}	1.52 ± 0.38 ^{bc}	0.65 ± 0.10 ^{c}	0.72 ± 0.05 ^{c}	0.89 ± 0.11 ^{c}
Dorea	2.96 ± 0.39 ^{a}	3.05 ± 0.42 ^{a}	3.23 ± 0.41 ^{a}	3.67 ± 0.40 ^{a}	4.44 ± 0.60 ^{a}	3.09 ± 0.07 ^{a}	4.53 ± 0.77 ^{a}	3.41 ± 0.75 ^{a}	1.49 ± 1.04 ^{a}	0.18 ± 0.03 ^{a}	0.99 ± 0.04 ^{a}	0.51 ± 0.06 ^{a}
Bacteroides	10.52 ± 0.40 ^{a}	11.09 ± 0.89 ^{a}	11.17 ± 0.18 ^{a}	11.54 ± 0.32 ^{a}	6.56 ± 0.35 ^{b}	6.86 ± 0.50 ^{b}	13.81 ± 0.88 ^{a}	11.46 ± 0.42 ^{a}	7.76 ± 3.82 ^{ab}	5.35 ± 0.43 ^{b}	3.63 ± 0.09 ^{b}	3.95 ± 0.52 ^{b}
Blautia	6.13 ± 0.71 ^{ab}	6.35 ± 0.4 ^{a}	6.22 ± 0.62 ^{a}	6.40 ± 0.34 ^{a}	3.37 ± 0.30 ^{bc}	2.31 ± 0.37 ^{cd}	2.48 ± 0.47 ^{c}	2.33 ± 0.13 ^{c}	3.11 ± 0.88 ^{bc}	0.83 ± 0.07 ^{d}	2.96 ± 0.27 ^{bc}	2.52 ± 0.69 ^{c}
Coprococcus	3.37 ± 0.18 ^{d}	3.29 ± 0.43 ^{d}	3.31 ± 0.15 ^{d}	3.03 ± 0.06 ^{d}	4.37 ± 0.45 ^{cd}	7.07 ± 0.15 ^{b}	5.54 ± 0.44 ^{bc}	6.11 ± 1.46 ^{bc}	2.68 ± 1.61 ^{d}	9.58 ± 0.09 ^{a}	2.55 ± 0.09 ^{d}	6.24 ± 0.35 ^{bc}
Ruminococcus	12.2 ± 0.54 ^{a}	11.77 ± 0.84 ^{a}	11.79 ± 1.09 ^{a}	12.21 ± 0.58 ^{a}	4.42 ± 0.09 ^{b}	4.44 ± 0.47 ^{b}	3.57 ± 0.24 ^{b}	3.92 ± 0.34 ^{b}	3.88 ± 0.05 ^{b}	2.15 ± 0.20 ^{c}	2.2 ± 0.22 ^{c}	2.94 ± 0.40 ^{bc}
Subdoligranulum	0.29 ± 0.15 ^{a}	0.31 ± 0.16 ^{a}	0.29 ± 0.14 ^{a}	0.35 ± 0.17 ^{a}	1.84 ± 0.50 ^{a}	1.44 ± 0.28 ^{a}	1.67 ± 0.41 ^{a}	1.77 ± 0.46 ^{a}	2.11 ± 0.52 ^{a}	0.7 ± 0.12 ^{a}	2.49 ± 0.48 ^{a}	2.33 ± 0.28 ^{a}
Fusicatenibacter	0.69 ± 0.02 ^{a}	0.72 ± 0.07 ^{a}	0.69 ± 0.02 ^{a}	0.73 ± 0.01 ^{a}	0.27 ± 0.02 ^{a}	0.31 ± 0.01 ^{a}	0.64 ± 0.07 ^{a}	0.79 ± 0.25 ^{a}	0.33 ± 0.23 ^{a}	0.06 ± 0.02 ^{a}	0.13 ± 0.01 ^{a}	0.15 ± 0.02 ^{a}
Erysipelotrichaceae_UCG-003	3.13 ± 0.49 ^{a}	3.1 ± 0.27 ^{a}	2.98 ± 0.22 ^{a}	2.83 ± 0.48 ^{ab}	1.61 ± 0.29 ^{ab}	1.56 ± 0.27 ^{ab}	0.72 ± 0.12 ^{b}	0.77 ± 0.20 ^{b}	1.83 ± 0.82 ^{ab}	1.15 ± 0.3 ^{ab}	1.38 ± 0.34 ^{ab}	1.23 ± 0.32 ^{ab}
Holdemanella	1.25 ± 0.09 ^{b}	1.23 ± 0.1 ^{b}	1.2 ± 0.12 ^{b}	1.19 ± 0.05 ^{b}	2.43 ± 0.2 ^{b}	2.32 ± 0.09 ^{b}	6.42 ± 0.53 ^{a}	6.87 ± 0.54 ^{a}	3.73 ± 2.34 ^{ab}	1.64 ± 0.12 ^{b}	3.5 ± 0.14 ^{b}	2.87 ± 0.24 ^{b}
UCG-002	1.92 ± 0.16 ^{a}	1.82 ± 0.16 ^{a}	1.97 ± 0.09 ^{a}	1.9 ± 0.20 ^{a}	6.10 ± 0.90 ^{a}	6.40 ± 0.85 ^{a}	4.61 ± 0.98 ^{a}	4.72 ± 0.78 ^{a}	4.97 ± 0.32 ^{a}	5.66 ± 0.45 ^{a}	4.67 ± 1.04 ^{a}	5.85 ± 0.79 ^{a}
Lachnoclostridium	0.43 ± 0.03 ^{b}	0.37 ± 0.03 ^{b}	0.35 ± 0.01 ^{b}	0.41 ± 0.01 ^{b}	2.37 ± 0.14 ^{a}	3.24 ± 0.15 ^{a}	3.5 ± 0.15 ^{a}	3.74 ± 0.48 ^{a}	3.37 ± 0.01 ^{a}	3.25 ± 0.19 ^{a}	6.00 ± 0.20 ^{a}	6.86 ± 1.03 ^{a}
Streptococcus	0.12 ± 0.02 ^{b}	0.12 ± 0.04 ^{b}	0.14 ± 0.01 ^{b}	0.13 ± 0.02 ^{b}	0.15 ± 0.05 ^{ab}	0.16 ± 0.07 ^{ab}	0.1 ± 0.03 ^{b}	0.09 ± 0.04 ^{b}	0.20 ± 0.08 ^{ab}	0.13 ± 0.01 ^{b}	0.20 ± 0.10 ^{ab}	0.21 ± 0.01 ^{a}
Lachnospiraceae_UCG-004	0.31 ± 0.06 ^{a}	0.25 ± 0.03 ^{a}	0.30 ± 0.05 ^{a}	0.30 ± 0.04 ^{a}	1.85 ± 0.12 ^{a}	1.50 ± 0.06 ^{a}	1.58 ± 0.06 ^{a}	1.86 ± 0.14 ^{a}	1.6 ± 0.05 ^{a}	0.57 ± 0.05 ^{a}	1.91 ± 0.10 ^{a}	2.09 ± 0.33 ^{a}
UBA1819	nd	0.02 ± 0.01 ^{a}	0.02 ± 0.01 ^{a}	0.02 ± 0.01 ^{a}	0.11 ± 0.01 ^{a}	0.16 ± 0.01 ^{a}	0.12 ± 0.04 ^{a}	0.12 ± 0.02 ^{a}	0.17 ± 0.03 ^{a}	0.44 ± 0.05 ^{a}	0.47 ± 0.10 ^{a}	0.32 ± 0.05 ^{a}
Sutterella	0.46 ± 0.02 ^{c}	0.53 ± 0.11 ^{bc}	0.46 ± 0.07 ^{bc}	0.54 ± 0.01 ^{b}	3.56 ± 0.24 ^{a}	3.91 ± 0.22 ^{a}	2.81 ± 0.06 ^{a}	3.42 ± 0.46 ^{a}	4.44 ± 0.40 ^{a}	3.84 ± 0.13 ^{a}	4.24 ± 0.19 ^{a}	3.58 ± 0.17 ^{a}
Roseburia	0.76 ± 0.03 ^{a}	0.71 ± 0.08 ^{a}	0.87 ± 0.04 ^{a}	0.75 ± 0.02 ^{a}	nd	nd	nd	nd	nd	nd	nd	nd
Barnesiella	0.34 ± 0.07 ^{a}	0.39 ± 0.01 ^{a}	0.33 ± 0.01 ^{a}	0.39 ± 0.06 ^{a}	0.21 ± 0.03 ^{a}	0.64 ± 0.10 ^{a}	1.04 ± 0.20 ^{a}	1.03 ± 0.03 ^{a}	0.52 ± 0.50 ^{a}	0.04 ± 0.02 ^{a}	0.16 ± 0.04 ^{a}	0.31 ± 0.07 ^{a}
Parabacteroides	0.93 ± 0.16 ^{d}	1.03 ± 0.07 ^{d}	0.99 ± 0.07 ^{d}	1.01 ± 0.02 ^{d}	2.57 ± 0.03 ^{a}	1.97 ± 0.03 ^{bc}	1.84 ± 0.14 ^{bc}	2.14 ± 0.14 ^{ab}	1.83 ± 0.32 ^{bcd}	0.49 ± 0.06 ^{d}	1.07 ± 0.07 ^{d}	1.19 ± 0.19 ^{cd}
Senegalimassilia	0.24 ± 0.06 ^{a}	0.24 ± 0.07 ^{a}	0.29 ± 0.05 ^{a}	0.25 ± 0.06 ^{a}	1.62 ± 0.20 ^{a}	1.50 ± 0.20 ^{a}	1.10 ± 0.20 ^{a}	1.09 ± 0.29 ^{a}	1.67 ± 0.56 ^{a}	1.40 ± 0.35 ^{a}	1.41 ± 0.23 ^{a}	1.63 ± 0.15 ^{a}
Lachnospira	2.26 ± 0.11 ^{a}	2.41 ± 0.11 ^{a}	2.20 ± 0.06 ^{a}	2.09 ± 0.06 ^{a}	0.21 ± 0.02 ^{b}	0.21 ± 0.02 ^{b}	0.15 ± 0.04 ^{b}	0.19 ± 0.03 ^{b}	0.24 ± 0.00 ^{b}	0.08 ± 0.00 ^{b}	0.15 ± 0.04 ^{b}	0.25 ± 0.04 ^{b}
Clostridium_sensu_stricto_1	0.16 ± 0.1 ^{a}	0.21 ± 0.07 ^{a}	0.20 ± 0.06 ^{a}	0.18 ± 0.04 ^{a}	0.25 ± 0.16 ^{a}	0.25 ± 0.12 ^{a}	0.16 ± 0.06 ^{a}	0.21 ± 0.07 ^{a}	0.37 ± 0.19 ^{a}	0.23 ± 0.10 ^{a}	0.31 ± 0.02 ^{a}	0.31 ± 0.05 ^{a}
Alistipes	0.50 ± 0.05 ^{c}	0.47 ± 0.05 ^{c}	0.50 ± 0.04 ^{c}	0.47 ± 0.02 ^{c}	0.74 ± 0.02 ^{c}	2.02 ± 0.01 ^{b}	3.80 ± 0.22 ^{a}	3.28 ± 0.36 ^{ab}	1.54 ± 1.40 ^{bc}	1.09 ± 0.09 ^{c}	2.14 ± 0.11 ^{b}	2.22 ± 0.28 ^{b}
Alloprevotella	2.72 ± 0.02 ^{a}	2.49 ± 0.6 ^{ab}	2.91 ± 0.3 ^{a}	2.7 ± 0.4 ^{a}	0.01 ± 0 ^{b}	0.02 ± 0 ^{b}	nd	nd	nd	nd	0.01 ± 0.00 ^{b}	0.01 ± 0.00 ^{b}
Oscillibacter	0.16 ± 0.04 ^{a}	0.11 ± 0.02 ^{a}	0.14 ± 0.01 ^{a}	0.15 ± 0.03 ^{a}	0.8 ± 0.15 ^{a}	0.75 ± 0.06 ^{a}	1.28 ± 0.26 ^{a}	1.22 ± 0.32 ^{a}	0.92 ± 0.37 ^{a}	0.54 ± 0.16 ^{a}	1.38 ± 0.46 ^{a}	1.37 ± 0.37 ^{a}
Catenibacterium	2.06 ± 0.33 ^{a}	1.99 ± 0.26 ^{a}	2.04 ± 0.10 ^{a}	1.94 ± 0.29 ^{a}	2.82 ± 1.03 ^{a}	2.44 ± 1.11 ^{a}	0.38 ± 0.24 ^{a}	0.78 ± 0.31 ^{a}	2.6 ± 1.65 ^{a}	1.65 ± 0.83 ^{a}	0.19 ± 0.07 ^{a}	0.26 ± 0.11 ^{a}
Klebsiella	nd	nd	nd	nd	nd	0.22 ± 0.08 ^{b}	0.01 ± 0.01 ^{b}	nd	nd	21.81 ± 1.69 ^{a}	nd	0.01 ± 0.00 ^{b}
Lachnospiraceae_ND3007_group	0.05 ± 0.01 ^{bc}	0.04 ± 0.03 ^{bc}	0.04 ± 0.02 ^{bc}	0.06 ± 0.02 ^{abc}	0.04 ± 0.01 ^{c}	0.03 ± 0.01 ^{c}	0.10 ± 0.01 ^{a}	0.09 ± 0.01 ^{ab}	0.05 ± 0.02 ^{abc}	0.03 ± 0.01 ^{c}	0.03 ± 0.01 ^{c}	0.04 ± 0.01 ^{c}
Anaerostipes	0.58 ± 0.27 ^{a}	0.53 ± 0.21 ^{a}	0.56 ± 0.22 ^{a}	0.46 ± 0.12 ^{a}	0.11 ± 0.04 ^{a}	0.07 ± 0.01 ^{a}	0.25 ± 0.03 ^{a}	0.20 ± 0.01 ^{a}	0.09 ± 0.05 ^{a}	0.00 ± 0.01 ^{a}	0.05 ± 0.02 ^{a}	0.07 ± 0.02 ^{a}
Lachnospiraceae_UCG-010	0.07 ± 0.01 ^{b}	0.06 ± 0.01 ^{b}	0.08 ± 0.02 ^{b}	0.03 ± 0.03 ^{b}	0.08 ± 0.04 ^{b}	0.21 ± 0.01 ^{a}	0.12 ± 0.03 ^{b}	0.12 ± 0.02 ^{b}	0.06 ± 0.06 ^{b}	0.05 ± 0.03 ^{b}	0.04 ± 0.00 ^{b}	0.05 ± 0.01 ^{b}
Prevotella	2.98 ± 0.41 ^{a}	2.76 ± 0.23 ^{a}	2.93 ± 0.12 ^{a}	2.78 ± 0.19 ^{a}	nd	nd	nd	nd	nd	nd	nd	nd
Romboutsia	0.12 ± 0.03 ^{a}	0.15 ± 0.05 ^{a}	0.14 ± 0.02 ^{a}	0.15 ± 0.02 ^{a}	0.20 ± 0.06 ^{a}	0.20 ± 0.07 ^{a}	0.14 ± 0.02 ^{a}	0.17 ± 0.08 ^{a}	0.34 ± 0.14 ^{a}	0.22 ± 0.04 ^{a}	0.25 ± 0.02 ^{a}	0.27 ± 0.01 ^{a}
Monoglobus	0.42 ± 0.08 ^{a}	0.42 ± 0.08 ^{a}	0.42 ± 0.03 ^{a}	0.38 ± 0.06 ^{a}	0.20 ± 0.02 ^{a}	0.16 ± 0.04 ^{a}	0.12 ± 0.01 ^{a}	0.13 ± 0.03 ^{a}	0.21 ± 0.06 ^{a}	0.1 ± 0.02 ^{a}	0.23 ± 0.05 ^{a}	0.2 ± 0.05 ^{a}
UCG-009	0.01 ± 0.01 ^{b}	0.02 ± 0.01 ^{b}	0.02 ± 0.01 ^{b}	0.01 ± 0.01 ^{b}	0.19 ± 0.02 ^{a}	0.24 ± 0.04 ^{a}	0.12 ± 0.04 ^{a}	0.13 ± 0.02 ^{a}	0.09 ± 0.00 ^{a}	0.08 ± 0.01 ^{a}	0.05 ± 0.01 ^{a}	0.09 ± 0.03 ^{a}
Butyricicoccus	0.44 ± 0.09 ^{ab}	0.49 ± 0.02 ^{a}	0.42 ± 0.05 ^{ab}	0.4 ± 0.02 ^{b}	0.15 ± 0.07 ^{bc}	0.04 ± 0.06 ^{cd}	0.06 ± 0.02 ^{bcd}	0.10 ± 0.03 ^{bc}	0.08 ± 0.00 ^{bc}	0.01 ± 0.01 ^{d}	0.14 ± 0.03 ^{bc}	0.15 ± 0.02 ^{bc}
UCG-003	0.11 ± 0.02 ^{a}	0.12 ± 0.03 ^{a}	0.12 ± 0.03 ^{a}	0.1 ± 0.03 ^{a}	0.03 ± 0.01 ^{a}	0.02 ± 0.00 ^{a}	0.11 ± 0.01 ^{a}	0.10 ± 0.03 ^{a}	0.04 ± 0.02 ^{a}	nd	0.03 ± 0.01 ^{a}	0.05 ± 0.01 ^{a}
Holdemania	0.05 ± 0.01 ^{c}	0.06 ± 0.02 ^{c}	0.05 ± 0.01 ^{c}	0.06 ± 0.02 ^{c}	0.17 ± 0.01 ^{b}	0.19 ± 0.03 ^{b}	0.29 ± 0.04 ^{ab}	0.35 ± 0.03 ^{a}	0.32 ± 0.04 ^{ab}	0.15 ± 0.01 ^{b}	0.25 ± 0.02 ^{ab}	0.34 ± 0.05 ^{ab}
UCG-005	0.08 ± 0.02 ^{a}	0.1 ± 0.01 ^{a}	0.11 ± 0.03 ^{a}	0.07 ± 0.01 ^{a}	0.07 ± 0.02 ^{a}	0.06 ± 0.01 ^{a}	0.09 ± 0.02 ^{a}	0.08 ± 0.01 ^{a}	0.08 ± 0.03 ^{a}	0.02 ± 0.01 ^{a}	0.08 ± 0.01 ^{a}	0.07 ± 0.01 ^{a}
Colidextribacter	0.04 ± 0.02 ^{a}	0.04 ± 0.01 ^{a}	0.05 ± 0.01 ^{a}	0.03 ± 0.01 ^{a}	0.05 ± 0.01 ^{a}	0.03 ± 0.02 ^{a}	0.10 ± 0.04 ^{a}	0.12 ± 0.04 ^{a}	0.04 ± 0.03 ^{a}	0.02 ± 0.02 ^{a}	0.03 ± 0.03 ^{a}	0.07 ± 0.01 ^{a}
Anaerofilum	nd	nd	nd	nd	0.08 ± 0.03 ^{a}	0.20 ± 0.05 ^{a}	0.30 ± 0.01 ^{a}	0.23 ± 0.03 ^{a}	0.16 ± 0.01 ^{a}	0.5 ± 0.04 ^{a}	0.88 ± 0.16 ^{a}	0.6 ± 0.16 ^{a}
Flavonifractor	0.02 ± 0.02 ^{b}	0.03 ± 0.01 ^{b}	0.03 ± 0.01 ^{b}	0.02 ± 0.02 ^{b}	0.1 ± 0.03 ^{b}	0.14 ± 0.03 ^{b}	0.34 ± 0.03 ^{a}	0.36 ± 0.05 ^{a}	0.24 ± 0.15 ^{ab}	0.16 ± 0.03 ^{ab}	0.18 ± 0.05 ^{ab}	0.28 ± 0.1 ^{ab}
Odoribacter	0.16 ± 0.01 ^{a}	0.14 ± 0.04 ^{abc}	0.13 ± 0.01 ^{abc}	0.14 ± 0.02 ^{ab}	0.03 ± 0.01 ^{c}	0.13 ± 0.02 ^{abc}	0.10 ± 0.03 ^{abc}	0.11 ± 0.02 ^{abc}	0.05 ± 0.04 ^{bc}	0.07 ± 0.02 ^{bc}	0.03 ± 0.01 ^{c}	0.05 ± 0.02 ^{c}
Incertae_Sedis	0.18 ± 0.01 ^{ab}	0.17 ± 0.01 ^{ab}	0.15 ± 0.01 ^{ab}	0.17 ± 0.01 ^{ab}	0.30 ± 0.03 ^{a}	0.29 ± 0.01 ^{a}	0.18 ± 0.02 ^{ab}	0.21 ± 0.01 ^{a}	0.26 ± 0.02 ^{a}	0.19 ± 0.03 ^{ab}	0.14 ± 0.01 ^{b}	0.18 ± 0.03 ^{ab}
Oscillospira	0.03 ± 0.01 ^{a}	0.02 ± 0.01 ^{a}	0.02 ± 0.01 ^{a}	0.03 ± 0.01 ^{a}	0.25 ± 0.02 ^{a}	0.29 ± 0.06 ^{a}	0.11 ± 0.02 ^{a}	0.1 ± 0.03 ^{a}	0.06 ± 0.04 ^{a}	0.01 ± 0.02 ^{a}	nd	nd
Christensenellaceae_R-7_group	0.24 ± 0.07 ^{a}	0.18 ± 0.02 ^{a}	0.21 ± 0.04 ^{a}	0.24 ± 0.07 ^{a}	0.48 ± 0.16 ^{a}	0.41 ± 0.09 ^{a}	0.27 ± 0.1 ^{a}	0.29 ± 0.14 ^{a}	0.54 ± 0.12 ^{a}	0.56 ± 0.18 ^{a}	0.56 ± 0.18 ^{a}	0.53 ± 0.13 ^{a}
Paraprevotella	0.29 ± 0.02 ^{a}	0.32 ± 0.05 ^{a}	0.32 ± 0.03 ^{a}	0.31 ± 0.02 ^{a}	nd	nd	nd	nd	nd	nd	nd	nd
Lachnospiraceae_FCS020_group	0.17 ± 0.03 ^{a}	0.18 ± 0.02 ^{a}	0.17 ± 0.02 ^{a}	0.15 ± 0.01 ^{a}	0.03 ± 0.00 ^{b}	nd	0.04 ± 0.01 ^{b}	0.04 ± 0.01 ^{b}	0.01 ± 0.01 ^{b}	nd	0.01 ± 0.02 ^{b}	0.04 ± 0.01 ^{b}
Lachnospiraceae_NK4A136_group	0.44 ± 0.04 ^{a}	0.45 ± 0.04 ^{a}	0.45 ± 0.09 ^{a}	0.44 ± 0.01 ^{a}	nd	nd	nd	nd	nd	nd	nd	nd
Tyzzerella	0.25 ± 0.02 ^{a}	0.21 ± 0.02 ^{a}	0.25 ± 0.03 ^{a}	0.25 ± 0.05 ^{a}	0.08 ± 0.01 ^{a}	0.05 ± 0.02 ^{a}	0.04 ± 0.00 ^{a}	0.04 ± 0.00 ^{a}	0.04 ± 0.01 ^{a}	0.00 ± 0.01 ^{a}	0.01 ± 0.00 ^{a}	0.01 ± 0.01 ^{a}
Rikenellaceae_RC9_gut_group	0.35 ± 0.15 ^{a}	0.27 ± 0.03 ^{a}	0.24 ± 0.03 ^{a}	0.22 ± 0.02 ^{a}	0.02 ± 0.00 ^{a}	0.10 ± 0.00 ^{a}	0.09 ± 0.04 ^{a}	0.12 ± 0.03 ^{a}	0.06 ± 0.06 ^{a}	0.02 ± 0.01 ^{a}	0.00 ± 0.00 ^{a}	0.07 ± 0.02 ^{a}
Butyricimonas	0.14 ± 0.04 ^{a}	0.13 ± 0.01 ^{a}	0.11 ± 0.01 ^{a}	0.14 ± 0.03 ^{a}	0.09 ± 0.01 ^{a}	0.10 ± 0.02 ^{a}	0.04 ± 0.02 ^{a}	0.07 ± 0.01 ^{a}	0.08 ± 0.01 ^{a}	0.01 ± 0.02 ^{a}	0.02 ± 0.01 ^{a}	0.02 ± 0.02 ^{a}
Fusobacterium	nd	nd	nd	nd	0.11 ± 0.01 ^{a}	0.05 ± 0.00 ^{a}	0.03 ± 0.01 ^{a}	0.03 ± 0.03 ^{a}	0.16 ± 0.11 ^{a}	0.05 ± 0.00 ^{a}	0.14 ± 0.00 ^{a}	0.06 ± 0.01 ^{a}
Mogibacterium	0.01 ± 0.00 ^{c}	nd	nd	nd	0.07 ± 0.02 ^{abc}	0.12 ± 0.01 ^{a}	0.04 ± 0.01 ^{bc}	0.04 ± 0.01 ^{bc}	0.10 ± 0.03 ^{ab}	0.03 ± 0.00 ^{bc}	0.09 ± 0.02 ^{ab}	0.11 ± 0.02 ^{ab}

VOLUNTEER # 2

	0h				24h				48h			
Genus	SW	SW + SO2	RW	RW + SO2	SW	SW + SO2	RW	RW + SO2	SW	SW + SO2	RW	RW + SO2
Bifidobacterium	1.34 ± 0.11 ^{c}	1.64 ± 0.19 ^{c}	1.53 ± 0.16 ^{c}	1.33 ± 0.09 ^{c}	1.69 ± 0.24 ^{c}	2.45 ± 0.23 ^{bc}	3.88 ± 0.35 ^{ab}	3.62 ± 0.25 ^{ab}	2.01 ± 0.42 ^{bc}	3.09 ± 0.34 ^{bc}	5.03 ± 0.32 ^{a}	4.68 ± 0.22 ^{a}
Enterococcus	2.89 ± 0.65 ^{b}	3.05 ± 0.75 ^{b}	2.94 ± 0.57 ^{b}	3.31 ± 0.83 ^{b}	20.3 ± 1.02 ^{a}	18.14 ± 1.73 ^{a}	18.23 ± 1.59 ^{a}	18.39 ± 1.33 ^{a}	19.84 ± 9.06 ^{a}	15.74 ± 0.23 ^{a}	24.75 ± 2.36 ^{a}	21.76 ± 1.86 ^{a}
Parasutterella	2.74 ± 0.37 ^{a}	3.08 ± 0.36 ^{a}	2.65 ± 0.37 ^{a}	2.96 ± 0.5 ^{a}	10.06 ± 0.12 ^{a}	7.91 ± 0.67 ^{a}	6.45 ± 0.42 ^{a}	6.91 ± 0.22 ^{a}	12.59 ± 2.99 ^{a}	10.11 ± 0.53 ^{a}	8.21 ± 0.2 ^{a}	8.77 ± 0.76 ^{a}
Bilophila	0.12 ± 0.02 ^{d}	0.12 ± 0.01 ^{d}	0.11 ± 0.02 ^{d}	0.1 ± 0.01 ^{d}	4.95 ± 0.43 ^{b}	5.04 ± 0.43 ^{b}	2.8 ± 0.23 ^{c}	3.01 ± 0.11 ^{c}	8 ± 0.32 ^{a}	8.39 ± 0.38 ^{a}	4.42 ± 0.25 ^{b}	5.04 ± 0.34 ^{b}
Agathobacter	16.32 ± 3.16 ^{a}	15.57 ± 1.78 ^{a}	15.88 ± 0.36 ^{a}	15.88 ± 1.37 ^{a}	0.51 ± 0.08 ^{a}	0.51 ± 0.04 ^{a}	0.64 ± 0.03 ^{a}	0.38 ± 0.04 ^{a}	0.24 ± 0.05 ^{a}	0.24 ± 0.03 ^{a}	0.18 ± 0.00 ^{a}	0.08 ± 0.01 ^{a}
Escherichia/Shigella	2.05 ± 0.60 ^{ef}	1.74 ± 0.37 ^{f}	2.02 ± 0.26 ^{f}	1.92 ± 0.37 ^{f}	8.94 ± 0.63 ^{bc}	15.93 ± 1.19 ^{a}	10.34 ± 0.31 ^{ab}	11.06 ± 0.51 ^{ab}	5.40 ± 0.21 ^{d}	11.82 ± 0.20 ^{ab}	4.5 ± 0.05 ^{de}	6.71 ± 0.46 ^{cd}
Phascolarctobacterium	1.39 ± 0.23 ^{c}	1.64 ± 0.16 ^{c}	1.10 ± 0.17 ^{c}	1.13 ± 0.17 ^{c}	5.45 ± 0.49 ^{b}	7.10 ± 1.02 ^{ab}	8.76 ± 0.50 ^{a}	9.57 ± 0.83 ^{a}	6.24 ± 0.96 ^{ab}	6.66 ± 0.47 ^{ab}	7.87 ± 0.72 ^{ab}	9.23 ± 1.02 ^{a}
Faecalibacterium	8.52 ± 1.99 ^{a}	8.09 ± 1.69 ^{a}	7.69 ± 1.33 ^{a}	7.71 ± 2.09 ^{a}	0.23 ± 0.07 ^{a}	0.21 ± 0.06 ^{a}	0.28 ± 0.11 ^{a}	0.31 ± 0.06 ^{a}	0.40 ± 0.14 ^{a}	0.20 ± 0.03 ^{a}	0.14 ± 0.05 ^{a}	0.17 ± 0.10 ^{a}
Dorea	3.26 ± 0.43 ^{ab}	2.96 ± 0.36 ^{ab}	3.55 ± 0.38 ^{a}	3.38 ± 0.53 ^{ab}	2.46 ± 0.94 ^{abc}	0.88 ± 0.08 ^{bc}	1.51 ± 0.51 ^{abc}	1.11 ± 0.36 ^{bc}	0.90 ± 0.12 ^{bc}	0.55 ± 0.04 ^{c}	1.21 ± 0.49 ^{bc}	0.59 ± 0.32 ^{c}
Bacteroides	17.00 ± 0.94 ^{a}	17.51 ± 1.91 ^{a}	17.05 ± 2.00 ^{a}	18.15 ± 1.36 ^{a}	14.24 ± 0.45 ^{a}	14.33 ± 0.72 ^{a}	13.94 ± 0.85 ^{a}	13.25 ± 0.33 ^{a}	12.74 ± 1.88 ^{a}	12.85 ± 0.69 ^{a}	13.73 ± 0.88 ^{a}	13.81 ± 0.89 ^{a}
Blautia	3.92 ± 0.88 ^{a}	4.28 ± 0.96 ^{a}	3.53 ± 1.27 ^{a}	3.35 ± 1.31 ^{a}	0.65 ± 0.28 ^{a}	0.70 ± 0.32 ^{a}	0.59 ± 0.10 ^{a}	0.37 ± 0.10 ^{a}	0.62 ± 0.21 ^{a}	0.43 ± 0.17 ^{a}	0.51 ± 0.13 ^{a}	0.25 ± 0.13 ^{a}
Coprococcus	0.16 ± 0.02 ^{b}	0.15 ± 0.01 ^{b}	0.14 ± 0.02 ^{b}	0.16 ± 0.02 ^{b}	0.07 ± 0.01 ^{c}	0.41 ± 0.04 ^{a}	0.73 ± 0.08 ^{a}	0.46 ± 0.06 ^{a}	0.06 ± 0.02 ^{c}	0.17 ± 0.02 ^{b}	0.31 ± 0.01 ^{a}	0.36 ± 0.04 ^{a}
Ruminococcus	3.61 ± 0.44 ^{a}	4.06 ± 0.46 ^{a}	3.89 ± 0.42 ^{a}	2.94 ± 0.31 ^{a}	0.38 ± 0.06 ^{b}	0.32 ± 0.05 ^{b}	0.12 ± 0.02 ^{c}	0.05 ± 0.01 ^{c}	0.39 ± 0.04 ^{b}	0.31 ± 0.02 ^{b}	0.11 ± 0.01 ^{c}	0.06 ± 0.01 ^{c}
Subdoligranulum	3.06 ± 1.28 ^{a}	2.89 ± 0.78 ^{a}	2.99 ± 1.09 ^{a}	3.00 ± 0.90 ^{a}	1.74 ± 0.17 ^{a}	0.60 ± 0.14 ^{a}	0.96 ± 0.06 ^{a}	0.60 ± 0.10 ^{a}	1.24 ± 0.37 ^{a}	0.37 ± 0.09 ^{a}	0.35 ± 0.12 ^{a}	0.19 ± 0.05 ^{a}
Fusicatenibacter	5.03 ± 1.39 ^{a}	4.96 ± 0.80 ^{a}	5.19 ± 0.92 ^{a}	5.28 ± 0.90 ^{a}	0.34 ± 0.02 ^{a}	0.26 ± 0.03 ^{a}	0.37 ± 0.06 ^{a}	0.39 ± 0.07 ^{a}	0.41 ± 0.03 ^{a}	0.32 ± 0.01 ^{a}	0.12 ± 0.01 ^{a}	0.07 ± 0.02 ^{a}
Erysipelotrichaceae_UCG-003	2.41 ± 0.38 ^{a}	2.30 ± 0.32 ^{a}	2.81 ± 0.18 ^{a}	2.38 ± 0.26 ^{a}	0.31 ± 0.03 ^{b}	0.06 ± 0.01 ^{c}	0.05 ± 0.02 ^{c}	0.02 ± 0.00 ^{c}	0.04 ± 0.01 ^{c}	0.01 ± 0.01 ^{c}	0.01 ± 0.01 ^{c}	nd
UCG-002	0.06 ± 0.02 ^{c}	0.04 ± 0.02 ^{c}	0.06 ± 0.00 ^{c}	0.05 ± 0.01 ^{c}	0.96 ± 0.12 ^{b}	0.37 ± 0.07 ^{bc}	0.44 ± 0.05 ^{b}	0.42 ± 0.02 ^{b}	1.34 ± 0.08 ^{b}	0.73 ± 0.07 ^{b}	1.87 ± 0.02 ^{a}	1.57 ± 0.23 ^{ab}
Lachnoclostridium	0.25 ± 0.05 ^{c}	0.27 ± 0.01 ^{c}	0.3 ± 0.01 ^{c}	0.31 ± 0.07 ^{c}	4.04 ± 0.5 ^{b}	3.89 ± 0.12 ^{b}	7.21 ± 0.35 ^{a}	7.46 ± 0.20 ^{a}	4.74 ± 0.21 ^{b}	4.78 ± 0.28 ^{b}	5.73 ± 0.50 ^{ab}	5.72 ± 0.59 ^{ab}
Streptococcus	2.28 ± 0.23 ^{a}	2.49 ± 0.29 ^{a}	2.44 ± 0.26 ^{a}	2.46 ± 0.33 ^{a}	1.31 ± 0.04 ^{a}	1.42 ± 0.22 ^{a}	0.77 ± 0.01 ^{bc}	0.76 ± 0.02 ^{c}	1.65 ± 0.30 ^{a}	2.00 ± 0.19 ^{a}	1.07 ± 0.11 ^{abc}	1.08 ± 0.06 ^{ab}
Lachnospiraceae_UCG-004	0.6 ± 0.06 ^{e}	0.59 ± 0.04 ^{e}	0.68 ± 0.13 ^{de}	0.68 ± 0.13 ^{de}	1.5 ± 0.09 ^{ab}	2.76 ± 0.26 ^{a}	0.91 ± 0.06 ^{cd}	0.88 ± 0.10 ^{cde}	0.73 ± 0.05 ^{cde}	1.37 ± 0.14 ^{bc}	0.20 ± 0.03 ^{f}	0.29 ± 0.06 ^{f}
UBA1819	0.04 ± 0.01 ^{a}	0.03 ± 0.00 ^{a}	0.04 ± 0.02 ^{a}	0.04 ± 0.03 ^{a}	1.35 ± 0.11 ^{a}	1.47 ± 0.17 ^{a}	1.11 ± 0.13 ^{a}	0.72 ± 0.03 ^{a}	2.10 ± 0.36 ^{a}	1.78 ± 0.24 ^{a}	1.71 ± 0.18 ^{a}	1.20 ± 0.14 ^{a}
Sutterella	nd	nd	nd	nd	nd	nd	0.01 ± 0.01 ^{a}	0.01 ± 0.00 ^{a}	nd	nd	0.02 ± 0.01 ^{a}	0.01 ± 0.01 ^{a}
Roseburia	2.93 ± 0.67 ^{ab}	2.42 ± 0.44 ^{ab}	3.58 ± 0.13 ^{a}	3.21 ± 0.66 ^{ab}	0.06 ± 0.01 ^{b}	0.09 ± 0.01 ^{b}	0.03 ± 0.03 ^{b}	0.02 ± 0.03 ^{b}	0.01 ± 0.02 ^{b}	nd	nd	nd
Barnesiella	1.02 ± 0.21 ^{a}	0.99 ± 0.17 ^{a}	0.98 ± 0.06 ^{a}	1.15 ± 0.06 ^{a}	0.29 ± 0.03 ^{c}	0.02 ± 0.01 ^{d}	0.36 ± 0.05 ^{bc}	0.42 ± 0.03 ^{b}	nd	nd	0.27 ± 0.06 ^{cd}	0.31 ± 0.01 ^{bc}
Parabacteroides	2.56 ± 0.23 ^{a}	2.57 ± 0.27 ^{a}	2.41 ± 0.34 ^{a}	2.72 ± 0.31 ^{a}	2.17 ± 0.23 ^{a}	1.53 ± 0.04 ^{a}	2.39 ± 0.06 ^{a}	2.44 ± 0.17 ^{a}	2.14 ± 0.36 ^{a}	2.04 ± 0.24 ^{a}	2.08 ± 0.12 ^{a}	2.28 ± 0.12 ^{a}
Lachnospira	2.9 ± 0.18 ^{a}	2.74 ± 0.34 ^{ab}	2.62 ± 0.38 ^{abc}	2.98 ± 0.26 ^{a}	0.54 ± 0.20 ^{de}	0.54 ± 0.03 ^{cd}	0.15 ± 0.03 ^{e}	0.12 ± 0.01 ^{e}	0.82 ± 0.15 ^{cd}	1.02 ± 0.08 ^{bcd}	0.19 ± 0.03 ^{e}	0.21 ± 0.03 ^{e}
Clostridium_sensu_stricto_1	0.42 ± 0.04 ^{a}	0.58 ± 0.08 ^{a}	0.40 ± 0.06 ^{a}	0.3 ± 0.03 ^{a}	0.03 ± 0.01 ^{b}	0.05 ± 0.02 ^{b}	0.09 ± 0.03 ^{b}	0.45 ± 0.19 ^{a}	0.04 ± 0.03 ^{b}	0.04 ± 0.02 ^{b}	0.02 ± 0.02 ^{b}	0.01 ± 0.01 ^{b}
Alistipes	1.10 ± 0.09 ^{a}	1.10 ± 0.10 ^{a}	1.08 ± 0.14 ^{a}	1.14 ± 0.15 ^{a}	0.91 ± 0.06 ^{a}	0.62 ± 0.07 ^{a}	0.96 ± 0.07 ^{a}	0.81 ± 0.09 ^{a}	0.07 ± 0.03 ^{b}	0.34 ± 0.04 ^{a}	0.87 ± 0.05 ^{a}	0.75 ± 0.08 ^{a}
Oscillibacter	0.14 ± 0.02 ^{a}	0.13 ± 0.02 ^{a}	0.19 ± 0.01 ^{a}	0.16 ± 0.04 ^{a}	0.3 ± 0.05 ^{a}	0.11 ± 0.03 ^{a}	0.43 ± 0.09 ^{a}	0.36 ± 0.06 ^{a}	0.53 ± 0.04 ^{a}	0.13 ± 0.03 ^{a}	0.7 ± 0.07 ^{a}	0.79 ± 0.13 ^{a}
Sellimonas	0.09 ± 0.02 ^{b}	0.08 ± 0.02 ^{b}	0.08 ± 0.02 ^{b}	0.09 ± 0.02 ^{b}	1.52 ± 0.34 ^{ab}	1.39 ± 0.44 ^{ab}	1.01 ± 0.06 ^{ab}	0.60 ± 0.13 ^{b}	1.86 ± 0.26 ^{a}	1.79 ± 0.55 ^{ab}	0.97 ± 0.10 ^{ab}	0.52 ± 0.17 ^{b}
Lachnospiraceae_ND3007_group	1.13 ± 0.13 ^{a}	1.23 ± 0.12 ^{a}	0.93 ± 0.19 ^{a}	0.90 ± 0.10 ^{a}	0.02 ± 0.02 ^{b}	0.02 ± 0.01 ^{b}	nd	nd	0.01 ± 0.01 ^{b}	0.01 ± 0.01 ^{b}	0.01 ± 0.01 ^{b}	nd
Anaerostipes	0.34 ± 0.20 ^{a}	0.37 ± 0.26 ^{a}	0.42 ± 0.35 ^{a}	0.41 ± 0.37 ^{a}	0.33 ± 0.15 ^{a}	0.22 ± 0.10 ^{a}	0.92 ± 0.10 ^{a}	0.68 ± 0.15 ^{a}	0.46 ± 0.06 ^{a}	0.34 ± 0.11 ^{a}	0.97 ± 0.17 ^{a}	0.72 ± 0.05 ^{a}
Lachnospiraceae_UCG-010	0.05 ± 0.02 ^{c}	0.04 ± 0.01 ^{c}	0.04 ± 0.02 ^{c}	0.06 ± 0.01 ^{c}	1.23 ± 0.26 ^{b}	0.08 ± 0.02 ^{c}	2.45 ± 0.15 ^{a}	2.93 ± 0.22 ^{a}	1.08 ± 0.05 ^{b}	0.07 ± 0.00 ^{c}	0.69 ± 0.04 ^{b}	2.09 ± 0.28 ^{ab}
Romboutsia	0.49 ± 0.21 ^{a}	0.55 ± 0.10 ^{a}	0.52 ± 0.16 ^{a}	0.48 ± 0.12 ^{a}	0.15 ± 0.08 ^{a}	0.21 ± 0.17 ^{a}	0.10 ± 0.04 ^{a}	0.09 ± 0.05 ^{a}	0.18 ± 0.04 ^{a}	0.22 ± 0.11 ^{a}	0.1 ± 0.01 ^{a}	0.1 ± 0.02 ^{a}
Monoglobus	0.55 ± 0.10 ^{a}	0.51 ± 0.13 ^{a}	0.57 ± 0.22 ^{a}	0.54 ± 0.18 ^{a}	nd	nd	nd	nd	nd	nd	nd	nd
UCG-009	0.1 ± 0.02 ^{d}	0.09 ± 0.03 ^{d}	0.1 ± 0.00 ^{cd}	0.09 ± 0.01 ^{d}	0.51 ± 0.02 ^{a}	0.45 ± 0.05 ab	0.25 ± 0.02 ^{bc}	0.27 ± 0.03 ^{bc}	0.57 ± 0.05 ^{a}	0.5 ± 0.06 ^{ab}	0.22 ± 0.05 ^{cd}	0.25 ± 0.00 ^{bc}
Eggerthella	0.46 ± 0.04 ^{a}	0.43 ± 0.08 ^{a}	0.54 ± 0.06 ^{a}	0.49 ± 0.06 ^{a}	0.52 ± 0.19 ^{a}	0.50 ± 0.23 a	0.38 ± 0.10 ^{a}	0.35 ± 0.13 ^{a}	0.69 ± 0.28 ^{a}	0.74 ± 0.41 ^{a}	0.57 ± 0.28 ^{a}	0.55 ± 0.24 ^{a}
Butyricicoccus	1.28 ± 0.11 ^{a}	1.26 ± 0.07 ^{a}	1.27 ± 0.13 ^{a}	1.24 ± 0.14 ^{a}	0.09 ± 0.02 ^{b}	0.08 ± 0.02 b	0.13 ± 0.05 ^{b}	0.16 ± 0.02 ^{b}	0.06 ± 0.03 ^{b}	0.10 ± 0.03 ^{b}	0.11 ± 0.05 ^{b}	0.16 ± 0.04 ^{b}
Holdemania	0.08 ± 0.01 ^{a}	0.09 ± 0.02 ^{a}	0.09 ± 0.01 ^{a}	0.09 ± 0.02 ^{a}	0.23 ± 0.03 ^{a}	0.19 ± 0.06 a	0.51 ± 0.09 ^{a}	0.34 ± 0.05 ^{a}	0.33 ± 0.12 ^{a}	0.30 ± 0.04 ^{a}	0.47 ± 0.07 ^{a}	0.36 ± 0.06 ^{a}
Weissella	0.35 ± 0.05 ^{ab}	0.4 ± 0.02 ^{a}	0.39 ± 0.01 ^{a}	0.42 ± 0.09 ^{a}	0.26 ± 0.02 ^{b}	0.25 ± 0.06 b	0.15 ± 0.01 ^{b}	0.14 ± 0.01 ^{b}	0.39 ± 0.03 ^{a}	0.49 ± 0.03 ^{a}	0.23 ± 0.03 ^{b}	0.21 ± 0.03 ^{b}
Veillonella	0.01 ± 0.01 ^{c}	nd	0.01 ± 0.01 ^{c}	nd	0.49 ± 0 ^{b}	1.08 ± 0.04 a	0.20 ± 0.03 ^{c}	0.07 ± 0.01 ^{c}	0.22 ± 0.07 ^{bc}	0.95 ± 0.05 ^{a}	0.04 ± 0.01 ^{c}	0.02 ± 0.02 ^{c}
Colidextribacter	0.24 ± 0.04 ^{a}	0.25 ± 0.02 ^{a}	0.24 ± 0.05 ^{a}	0.24 ± 0.04 ^{a}	0.73 ± 0.33 ^{a}	0.3 ± 0.18 a	0.18 ± 0.04 ^{a}	0.24 ± 0.04 ^{a}	0.59 ± 0.48 ^{a}	0.31 ± 0.26 ^{a}	0.35 ± 0.07 ^{a}	0.42 ± 0.06 ^{a}
Flavonifractor	0.13 ± 0.03 ^{a}	0.16 ± 0.02 ^{a}	0.13 ± 0.03 ^{a}	0.13 ± 0.02 ^{a}	0.33 ± 0.07 ^{a}	0.32 ± 0.09 a	0.29 ± 0.08 ^{a}	0.25 ± 0.06 ^{a}	0.32 ± 0.1 ^{a}	0.31 ± 0.07 ^{a}	0.24 ± 0.05 ^{a}	0.21 ± 0.09 ^{a}
Negativibacillus	0.11 ± 0.01 ^{a}	0.19 ± 0.12 ^{a}	0.18 ± 0.04 ^{a}	0.11 ± 0.08 ^{a}	nd	nd	nd	nd	nd	nd	nd	nd
CHKCI002	0.18 ± 0.03 ^{ab}	0.18 ± 0.07 ^{ab}	0.23 ± 0.03 ^{ab}	0.17 ± 0.06 ^{ab}	0.2 ± 0.04 ^{ab}	0.18 ± 0.01 ab	0.10 ± 0.01 ^{b}	0.13 ± 0.02 ^{b}	0.27 ± 0.01 ^{a}	0.30 ± 0.04 ^{a}	0.17 ± 0.01 ^{ab}	0.19 ± 0.05 ^{ab}
Candidatus_Soleaferrea	0.02 ± 0.00 ^{b}	nd	0.02 ± 0.00 ^{b}	0.02 ± 0.02 ^{b}	0.26 ± 0.02 ^{a}	0.20 ± 0.01 a	0.18 ± 0.02 ^{a}	0.14 ± 0.01 ^{a}	0.30 ± 0.04 ^{a}	0.14 ± 0.02 ^{a}	0.22 ± 0.03 ^{a}	0.13 ± 0.02 ^{a}
Odoribacter	0.11 ± 0.02 ^{a}	0.08 ± 0.02 ^{a}	0.10 ± 0.02 ^{a}	0.09 ± 0.01 ^{a}	0.14 ± 0.03 ^{a}	0.19 ± 0.08 a	0.14 ± 0.02 ^{a}	0.14 ± 0.02 ^{a}	0.28 ± 0.11 ^{a}	0.20 ± 0.02 ^{a}	0.17 ± 0.09 ^{a}	0.13 ± 0.05 ^{a}
Incertae_Sedis	0.14 ± 0.01 ^{a}	0.14 ± 0.01 ^{a}	0.12 ± 0.02 ^{a}	0.14 ± 0.04 ^{a}	0.13 ± 0.02 ^{a}	0.16 ± 0.01 a	0.30 ± 0.04 ^{a}	0.26 ± 0.03 ^{a}	0.15 ± 0.05 ^{a}	0.20 ± 0.02 ^{a}	0.24 ± 0.06 ^{a}	0.18 ± 0.04 ^{a}
Oscillospira	0.14 ± 0.03 ^{abc}	0.13 ± 0.03 ^{abc}	0.18 ± 0.01 ^{a}	0.15 ± 0.02 ^{ab}	0.13 ± 0.04 ^{abc}	0.05 ± 0.00 c	0.07 ± 0.01 ^{bc}	0.08 ± 0.02 ^{bc}	0.03 ± 0.00 ^{c}	0.01 ± 0.01 ^{c}	0.03 ± 0.01 ^{c}	0.03 ± 0.01 ^{c}
Erysipelatoclostridium	0.28 ± 0.02 ^{abc}	0.24 ± 0.03 ^{abc}	0.26 ± 0.07 ^{abc}	0.27 ± 0.08 ^{abc}	0.15 ± 0.05 ^{bcd}	0.13 ± 0.03 cd	0.56 ± 0.09 ^{a}	0.42 ± 0.06 ^{a}	0.08 ± 0.02 ^{d}	0.09 ± 0.03 ^{d}	0.34 ± 0.05 ^{ab}	0.33 ± 0.04 ^{ab}
Citrobacter	nd	nd	0.01 ± 0.01 ^{b}	nd	0.24 ± 0.05 ^{ab}	0.31 ± 0.07 ab	0.16 ± 0.01 ^{ab}	0.12 ± 0.02 ^{b}	0.43 ± 0.06 ^{a}	0.53 ± 0.07 ^{a}	0.19 ± 0.02 ^{ab}	0.12 ± 0.01 ^{ab}
Leuconostoc	0.08 ± 0.02 ^{b}	0.12 ± 0.04 ^{ab}	0.13 ± 0.06 ^{ab}	0.10 ± 0.03 ^{b}	0.14 ± 0.02 ^{ab}	0.11 ± 0.01 b	0.07 ± 0.01 ^{b}	0.07 ± 0.02 ^{b}	0.22 ± 0.04 ^{ab}	0.22 ± 0.02 ^{a}	0.10 ± 0.00 ^{b}	0.09 ± 0.01 ^{b}
Allisonella	0.13 ± 0.02 ^{c}	0.13 ± 0.01 ^{c}	0.14 ± 0.03 ^{bc}	0.13 ± 0.03 ^{c}	0.10 ± 0.01 ^{c}	0.19 ± 0.03 bc	0.29 ± 0.01 ^{b}	0.84 ± 0.02 ^{a}	0.02 ± 0.00 ^{d}	0.03 ± 0.01 ^{d}	nd	nd
DTU089	0.05 ± 0.02 ^{a}	0.05 ± 0.02 ^{a}	0.06 ± 0.02 ^{a}	0.07 ± 0.03 ^{a}	0.04 ± 0.01 ^{a}	0.05 ± 0.01 a	0.07 ± 0.03 ^{a}	0.08 ± 0.02 ^{a}	0.05 ± 0.01 ^{a}	0.05 ± 0.01 ^{a}	0.10 ± 0.01 ^{a}	0.11 ± 0.01 ^{a}
Merdibacter	0.13 ± 0.04 ^{a}	0.15 ± 0.03 ^{a}	0.11 ± 0.02 ^{a}	0.1 ± 0.04 ^{a}	0.06 ± 0.03 ^{a}	0.09 ± 0.09 a	0.05 ± 0.05 ^{a}	0.06 ± 0.03 ^{a}	0.06 ± 0.02 ^{a}	0.07 ± 0.01 ^{a}	0.06 ± 0.04 ^{a}	0.06 ± 0.03 ^{a}
Eisenbergiella	nd	nd	nd	0.01 ± 0.01 ^{b}	0.01 ± 0.01 ^{b}	0.02 ± 0.02 b	0.14 ± 0.00 ^{ab}	0.13 ± 0.02 ^{ab}	0.01 ± 0.01 ^{b}	0.03 ± 0.02 ^{b}	0.16 ± 0.04 ^{ab}	0.17 ± 0.02 ^{a}
UCG-008	0.15 ± 0.04 ^{a}	0.13 ± 0.01 ^{a}	0.1 ± 0.01 ^{a}	0.1 ± 0.02 ^{a}	nd	nd	0.03 ± 0.04 ^{a}	0.01 ± 0.01 ^{a}	nd	nd	nd	nd
Butyricimonas	0.07 ± 0.02 ^{b}	0.07 ± 0.01 ^{b}	0.06 ± 0.01 ^{b}	0.07 ± 0.01 ^{b}	0.11 ± 0.01 ^{b}	0.13 ± 0.03 ab	0.06 ± 0.02 ^{b}	0.04 ± 0.02 ^{b}	0.18 ± 0.05 ^{ab}	0.22 ± 0.01 ^{a}	0.08 ± 0.04 ^{b}	0.06 ± 0.02 ^{b}

VOLUNTEER # 3

Genus	0h				24h				48h			
	SW	SW + SO2	RW	RW + SO2	SW	SW + SO2	RW	RW + SO2	SW	SW + SO2	RW	RW + SO2
Collinsella	3.48 ± 0.18 ^{c}	3.54 ± 0.34 ^{c}	3.81 ± 0.48 ^{c}	3.63 ± 0.15 ^{c}	8.79 ± 0.79 ^{b}	8.14 ± 0.75 ^{b}	7.75 ± 0.58 ^{b}	8.83 ± 0.52 ^{b}	11.75 ± 1.11 ^{ab}	11.98 ± 1.51 ^{ab}	11.62 ± 1.38 ^{ab}	12.07 ± 0.21 ^{a}
Bifidobacterium	4.15 ± 0.29 ^{b}	4.33 ± 0.11 ^{b}	4.61 ± 0.55 ^{b}	4.92 ± 0.35 ^{b}	7.05 ± 0.41 ^{a}	6.61 ± 0.26 ^{a}	8.87 ± 0.58 ^{a}	8.36 ± 0.44 ^{a}	10.64 ± 0.72 ^{a}	9.83 ± 0.10 ^{a}	12.23 ± 1.15 ^{a}	12.32 ± 1.15 ^{a}
Enterococcus	0.03 ± 0.00 ^{b}	0.03 ± 0.01 ^{b}	0.03 ± 0.01 ^{b}	0.02 ± 0.01 ^{b}	0.07 ± 0.02 ^{b}	0.05 ± 0.00 ^{b}	0.07 ± 0.01 ^{b}	0.07 ± 0.02 ^{b}	0.11 ± 0.02 ^{ab}	0.09 ± 0.01 ^{b}	0.14 ± 0.01 ^{a}	0.13 ± 0.01 ^{a}
Parasutterella	0.90 ± 0.12 ^{e}	0.94 ± 0.10 ^{e}	0.95 ± 0.06 ^{e}	1.08 ± 0.09 ^{e}	5.46 ± 0.30 ^{d}	8.09 ± 0.30 ^{ab}	4.32 ± 0.28 ^{d}	5.03 ± 0.29 ^{d}	7.06 ± 0.20 ^{bc}	9.60 ± 0.01 ^{a}	5.61 ± 0.34 ^{cd}	5.37 ± 0.18 ^{d}
Bilophila	0.04 ± 0.03 ^{d}	0.08 ± 0.02 ^{d}	0.04 ± 0.04 ^{d}	0.06 ± 0.06 ^{d}	3.79 ± 0.02 ^{b}	3.45 ± 0.31 ^{bc}	2.12 ± 0.16 ^{c}	2.13 ± 0.10 ^{c}	6.72 ± 0.38 ^{a}	6.43 ± 0.51 ^{a}	3.8 ± 0.22 ^{b}	3.72 ± 0.10 ^{b}
Agathobacter	5.53 ± 0.46 ^{ab}	5.81 ± 0.37 ^{a}	5.26 ± 0.58 ^{abc}	5.60 ± 0.20 ^{a}	3.95 ± 0.26 ^{bc}	2.72 ± 0.15 ^{cd}	2.06 ± 0.13 ^{de}	2.81 ± 0.24 ^{cd}	1.44 ± 0.18 ^{ef}	1.79 ± 0.17 ^{def}	1.21 ± 0.06 ^{f}	1.30 ± 0.08 ^{f}
Escherichia/Shigella	9.64 ± 0.33 ^{a}	7.89 ± 0.67 ^{a}	8.74 ± 1.33 ^{a}	8.78 ± 1.24 ^{a}	1.45 ± 0.08 ^{c}	2.09 ± 0.07 ^{b}	0.63 ± 0.05 ^{d}	0.25 ± 0.02 ^{e}	0.16 ± 0.01 ^{f}	0.38 ± 0.00 ^{d}	0.08 ± 0.02 ^{g}	0.09 ± 0.01 ^{g}
Faecalibacterium	7.24 ± 0.61 ^{a}	8.17 ± 1.13 ^{a}	6.96 ± 0.60 ^{a}	7.39 ± 0.66 ^{a}	2.00 ± 0.16 ^{ab}	2.28 ± 0.09 ^{a}	1.34 ± 0.09 ^{b}	1.82 ± 0.19 ^{ab}	1.45 ± 0.06 ^{ab}	1.56 ± 0.00 ^{ab}	1.19 ± 0.02 ^{b}	1.36 ± 0.07 ^{b}
Dorea	2.00± 0.21 ^{b}	2.02 ± 0.25 ^{b}	1.95 ± 0.33 ^{b}	1.77 ± 0.10 ^{b}	5.13 ± 0.53 ^{a}	9.43 ± 1.12 ^{a}	8.00 ± 0.35 ^{a}	8.45 ± 0.46 ^{a}	0.76 ± 0.06 ^{b}	1.43 ± 0.14 ^{b}	1.28 ± 0.09 ^{b}	1.83 ± 0.14 ^{b}
Bacteroides	23.52 ± 1.22 ^{ab}	22.73 ± 1.46 ^{ab}	23.61 ± 1.68 ^{ab}	24.26 ± 0.44 ^{a}	22.54 ± 0.87 ^{ab}	19.51 ± 0.99 ^{bc}	23.79 ± 0.88 ^{ab}	22.13 ± 0.71 ^{ab}	15.9 ± 0.93 ^{c}	11.34 ± 0.51 ^{d}	19.27 ± 1.24 ^{bc}	20.14 ± 0.89 ^{abc}
Blautia	2.77 ± 0.41 ^{abc}	3.01 ± 0.25 ^{ab}	2.7 ± 0.38 ^{abc}	2.46 ± 0.01 ^{abc}	2.27 ± 0.15 ^{bc}	2.13 ± 0.18 ^{bc}	1.43 ± 0.04 ^{c}	1.55 ± 0.10 ^{c}	2.92 ± 0.13 ^{ab}	3.19 ± 0.21 ^{a}	1.73 ± 0.23 ^{c}	1.76 ± 0.02 ^{bc}
Coprococcus	1.22 ± 0.09 ^{c}	1.23 ± 0.13 ^{c}	1.13 ± 0.06 ^{c}	1.21 ± 0.05 ^{c}	2.69 ± 0.17 ^{a}	3.00 ± 0.16 ^{a}	1.04 ± 0.08 ^{c}	1.84 ± 0.10 ^{b}	0.90 ± 0.14 ^{c}	0.72 ± 0.02 ^{c}	0.76 ± 0.03 ^{c}	1.07 ± 0.01 ^{c}
Ruminococcus	4.54 ± 0.2 ^{a}	4.79 ± 0.13 ^{a}	4.50 ± 0.05 ^{a}	4.36 ± 0.08 ^{a}	1.63 ± 0.06 ^{b}	1.57 ± 0.06 ^{b}	0.88 ± 0.07 ^{cd}	0.90 ± 0.05 ^{c}	1.60 ± 0.17 ^{b}	1.56 ± 0.00 ^{b}	0.65 ± 0.04 ^{d}	0.71 ± 0.04 ^{cd}
Subdoligranulum	2.5 ± 0.69 ^{a}	2.53 ± 0.84 ^{a}	2.39 ± 0.68 ^{a}	2.35 ± 0.86 ^{a}	3.31 ± 0.79 ^{a}	3.26 ± 0.90 ^{a}	3.94 ± 0.99 ^{a}	4.58 ± 1.20 ^{a}	3.32 ± 1.09 ^{a}	3.89 ± 1.00 ^{a}	4.00 ± 1.15 ^{a}	6.01 ± 1.05 ^{a}
Fusicatenibacter	1.12 ± 0.06 ^{a}	1.19 ± 0.25 ^{a}	1.15 ± 0.09 ^{a}	1.03 ± 0.03 ^{a}	0.64 ± 0.04 ^{b}	0.65 ± 0.04 ^{b}	1.28 ± 0.06 ^{a}	1.15 ± 0.03 ^{a}	0.35 ± 0.02 ^{c}	0.39 ± 0.03 ^{c}	0.58 ± 0.01 ^{b}	0.51 ± 0.06 ^{bc}
Acidaminococcus	1.41 ± 0.21 ^{d}	1.49 ± 0.20 ^{d}	1.47 ± 0.05 ^{d}	1.55 ± 0.02 ^{d}	7.30 ± 0.47 ^{ab}	6.99 ± 0.53 ^{abc}	4.78 ± 0.30 ^{c}	1.36 ± 0.11 ^{d}	8.22 ± 0.22 ^{a}	7.70 ± 0.44 ^{ab}	5.77 ± 0.37 ^{bc}	0.37 ± 0.05 ^{e}
Erysipelotrichaceae_UCG-003	0.94 ± 0.05 ^{a}	0.76 ± 0.05 ^{a}	0.91 ± 0.13 ^{a}	0.87 ± 0.06 ^{a}	0.12 ± 0.02 ^{b}	0.20 ± 0.01 ^{b}	0.78 ± 0.22 ^{a}	0.87 ± 0.30 ^{a}	0.04 ± 0.02 ^{b}	0.04 ± 0.01 ^{b}	0.34 ± 0.13 ^{ab}	0.67 ± 0.18 ^{ab}
UCG-002	1.08 ± 0.08 ^{b}	1.02 ± 0.06 ^{b}	1.04 ± 0.07 ^{b}	0.92 ± 0.13 ^{b}	2.99 ± 0.09 ^{a}	2.94 ± 0.46 ^{a}	2.85 ± 0.14 ^{a}	3.55 ± 0.19 ^{a}	3.61 ± 0.25 ^{a}	4.20 ± 0.47 ^{a}	3.35 ± 0.35 ^{a}	4.14 ± 0.29 ^{a}
Lachnoclostridium	0.67 ± 0.05 ^{d}	0.67 ± 0.09 ^{d}	0.66 ± 0.10 ^{d}	0.62 ± 0.07 ^{d}	1.66 ± 0.07 ^{bc}	1.64 ± 0.04 ^{bc}	1.76 ± 0.04 ^{bc}	1.58 ± 0.13 ^{c}	3.44 ± 0.43 ^{a}	3.36 ± 0.08 ^{a}	3.52 ± 0.27 ^{a}	2.71 ± 0.22 ^{ab}
Streptococcus	0.21 ± 0.02 ^{bc}	0.23 ± 0.02 ^{b}	0.24 ± 0.04 ^{b}	0.26 ± 0.02 ^{b}	0.17 ± 0.03 ^{bc}	0.2 ± 0.03 ^{bc}	0.16 ± 0.03 ^{bc}	0.14 ± 0.02 ^{c}	0.37 ± 0.02 ^{a}	0.28 ± 0.02 ^{ab}	0.26 ± 0.03 ^{ab}	0.27 ± 0.02 ^{ab}
Lachnospiraceae_UCG-004	0.36 ± 0.06 ^{b}	0.45 ± 0.05 ^{b}	0.44 ± 0.07 ^{b}	0.37 ± 0.05 ^{b}	1.01 ± 0.02 ^{a}	0.86 ± 0.02 ^{a}	0.87 ± 0.10 ^{a}	1.05 ± 0.07 ^{a}	0.93 ± 0.04 ^{a}	1.03 ± 0.09 ^{a}	1.20 ± 0.10 ^{a}	1.09 ± 0.03 ^{a}
Dialister	2.48 ± 0.11 ^{a}	2.64 ± 0.08 ^{a}	2.78 ± 0.13 ^{a}	2.61 ± 0.08 ^{a}	0.87 ± 0.01 ^{b}	0.39 ± 0.03 ^{c}	0.71 ± 0.04 ^{b}	0.34 ± 0.02 ^{c}	0.59 ± 0.06 ^{bc}	0.37 ± 0.03 ^{c}	0.70 ± 0.03 ^{b}	0.28 ± 0.01 ^{c}
UBA1819	nd	nd	nd	nd	0.07 ± 0.01 ^{c}	0.06 ± 0.01 ^{c}	0.11 ± 0.01 ^{bc}	0.07 ± 0.02 ^{bc}	0.15 ± 0.03 ^{ab}	0.14 ± 0.00 ^{ab}	0.16 ± 0.01 ^{a}	0.15 ± 0.01 ^{ab}
Sutterella	0.42 ± 0.07 ^{cd}	0.35 ± 0.07 ^{de}	0.48 ± 0.03 ^{cd}	0.46 ± 0.04 ^{cd}	0.94 ± 0.04 ^{ab}	0.91 ± 0.03 ^{b}	0.57 ± 0.02 ^{c}	0.38 ± 0.04 ^{d}	0.87 ± 0.01 ^{b}	1.11 ± 0.03 ^{a}	0.54 ± 0.05 ^{cd}	0.04 ± 0.01 ^{e}
Roseburia	2.47 ± 0.20 ^{a}	2.63 ± 0.21 ^{a}	2.55 ± 0.27 ^{a}	2.71 ± 0.10 ^{a}	0.01 ± 0.02 ^{b}	0.06 ± 0.02 ^{b}	0.01 ± 0.02 ^{b}	0.04 ± 0.03 ^{b}	nd	nd	nd	0.01 ± 0.01 ^{b}
Barnesiella	0.22 ± 0.01 ^{c}	0.21 ± 0.01 ^{c}	0.22 ± 0.05 ^{bc}	0.22 ± 0.02 ^{bc}	0.10 ± 0.03 ^{de}	0.13 ± 0.01 ^{d}	0.32 ± 0.02 ^{a}	0.29 ± 0.01 ^{ab}	nd	nd	0.03 ± 0.00 ^{e}	0.03 ± 0.00 ^{e}
Parabacteroides	0.55 ± 0.07 ^{f}	0.52 ± 0.10 ^{f}	0.58 ± 0.04 ^{f}	0.59 ± 0.07 ^{ef}	1.59 ± 0.04 ^{bc}	1.39 ± 0.11 ^{cd}	2.11 ± 0.08 ^{a}	1.94 ± 0.13 ^{abc}	2.13 ± 0.09 ^{a}	1.97 ± 0.12 ^{ab}	0.97 ± 0.02 ^{de}	1.1 ± 0.02 ^{d}
Lachnospira	1.34 ± 0.17 ^{a}	1.36 ± 0.25 ^{a}	1.15 ± 0.10 ^{a}	1.19 ± 0.04 ^{a}	0.06 ± 0.03 ^{a}	0.05 ± 0.02 ^{a}	0.04 ± 0.03 ^{a}	0.06 ± 0.03 ^{a}	0.09 ± 0.02 ^{a}	0.12 ± 0.00 ^{a}	0.07 ± 0.03 ^{a}	0.09 ± 0.00 ^{a}
Faecalicoccus	0.05 ± 0.01 ^{c}	0.07 ± 0.03 ^{c}	0.06 ± 0.01 ^{c}	0.08 ± 0.01 ^{c}	0.17 ± 0.01 ^{b}	0.15 ± 0.04 ^{bc}	2.12 ± 0.31 ^{a}	2.73 ± 0.26 ^{a}	0.14 ± 0.05 ^{bc}	0.13 ± 0.04 ^{bc}	2.77 ± 0.11 ^{a}	2.73 ± 0.46 ^{a}
Clostridium_sensu_stricto_1	4.79 ± 0.12 ^{a}	4.88 ± 0.12 ^{a}	4.78 ± 0.52 ^{a}	4.72 ± 0.11 ^{a}	0.18 ± 0.03 ^{bc}	0.15 ± 0.02 ^{c}	0.14 ± 0.02 ^{c}	0.16 ± 0.02 ^{bc}	0.22 ± 0.04 ^{bc}	0.23 ± 0.01 ^{b}	0.16 ± 0.00 ^{c}	0.17 ± 0.01 ^{bc}
Alistipes	0.51 ± 0.08 ^{bc}	0.47 ± 0.05 ^{c}	0.51 ± 0.04 ^{c}	0.51 ± 0.02 ^{c}	0.52 ± 0.06 ^{bc}	0.49 ± 0.06 ^{c}	0.88 ± 0.02 ^{a}	0.76 ± 0.02 ^{b}	0.33 ± 0.04 ^{c}	0.15 ± 0.03 ^{d}	0.34 ± 0.02 ^{c}	0.36 ± 0.00 ^{c}
Oscillibacter	0.05 ± 0.00 ^{a}	0.05 ± 0.00 ^{a}	0.07 ± 0.01 ^{a}	0.08 ± 0.02 ^{a}	0.21 ± 0.03 ^{a}	0.10 ± 0.03 ^{a}	0.42 ± 0.04 ^{a}	0.32 ± 0.04 ^{a}	0.23 ± 0.02 ^{a}	0.15 ± 0.02 ^{a}	0.55 ± 0.09 ^{a}	0.4 ± 0.03 ^{a}
NK4A214_group	0.32 ± 0.05 ^{d}	0.29 ± 0.00 ^{d}	0.3 ± 0.04 ^{d}	0.27 ± 0.02 ^{d}	0.35 ± 0.01 ^{d}	0.35 ± 0.02 ^{d}	0.81 ± 0.05 ^{c}	1.58 ± 0.11 ^{b}	0.54 ± 0.09 ^{cd}	0.55 ± 0.06 ^{cd}	1.27 ± 0.10 ^{b}	2.31 ± 0.11 ^{a}
Klebsiella	0.07 ± 0.02 ^{a}	0.07 ± 0.02 ^{a}	0.05 ± 0.01 ^{a}	0.06 ± 0.01 ^{a}	0.07 ± 0.01 ^{a}	0.07 ± 0.00 ^{a}	0.13 ± 0.02 ^{a}	0.14 ± 0.04 ^{a}	0.1 ± 0.02 ^{a}	0.05 ± 0.00 ^{a}	0.02 ± 0.01 ^{a}	0.01 ± 0.01 ^{a}
Lachnospiraceae_ND3007_group	0.44 ± 0.03 ^{a}	0.56 ± 0.11 ^{a}	0.45 ± 0.04 ^{a}	0.4 ± 0.08 ^{a}	0.04 ± 0.01 ^{a}	0.06 ± 0.02 ^{a}	0.03 ± 0.00 ^{a}	0.04 ± 0.01 ^{a}	0.05 ± 0.01 ^{a}	0.05 ± 0.01 ^{a}	0.02 ± 0.01 ^{a}	0.02 ± 0.00 ^{a}
Lachnospiraceae_UCG-010	nd	0.01 ± 0.01 ^{cd}	nd	nd	0.07 ± 0.01 ^{c}	0.04 ± 0.01 ^{cd}	0.27 ± 0.01 ^{b}	0.26 ± 0.01 ^{b}	0.05 ± 0.01 ^{cd}	0.06 ± 0.01 ^{cd}	0.4 ± 0.01 ^{a}	0.32 ± 0.01 ^{b}
Romboutsia	1.11 ± 0.05 ^{a}	1.08 ± 0.14 ^{a}	1.11 ± 0.13 ^{a}	1.01 ± 0.07 ^{a}	0.06 ± 0.03 ^{b}	0.02 ± 0.02 ^{b}	0.05 ± 0.04 ^{b}	0.03 ± 0.01 ^{b}	0.1 ± 0.03 ^{b}	0.08 ± 0.03 ^{b}	0.06 ± 0.02 ^{b}	0.06 ± 0.01 ^{b}
Monoglobus	0.34 ± 0.01 ^{a}	0.31 ± 0.00 ^{a}	0.33 ± 0.04 ^{a}	0.33 ± 0.01 ^{a}	0.10 ± 0.03 ^{b}	0.09 ± 0.01 ^{b}	0.06 ± 0.03 ^{b}	0.07 ± 0.02 ^{b}	0.04 ± 0.01 ^{b}	0.04 ± 0.00 ^{b}	0.04 ± 0.01 ^{b}	0.06 ± 0.03 ^{b}
UCG-009	0.04 ± 0.02 ^{abc}	0.04 ± 0.00 ^{abc}	0.03 ± 0 ^{c}	0.04 ± 0.01 ^{abc}	0.05 ± 0.01 ^{ab}c	0.03 ± 0.01 ^{bc}	0.07 ± 0.01 ^{ab}	0.04 ± 0.01 ^{abc}	0.07 ± 0.01 ^{ab}	0.05 ± 0.00 ^{ab}	0.11 ± 0.02 ^{a}	0.07 ± 0.02 ^{ab}
Phocea	nd	nd	nd	nd	0.50 ± 0.06 ^{abc}	0.49 ± 0.03 ^{bc}	0.40 ± 0.03 ^{c}	0.26 ± 0.02 ^{c}	0.77 ± 0.06 ^{ab}	0.78 ± 0.05 ^{a}	0.59 ± 0.04 ^{ab}	0.46 ± 0.07 ^{bc}
Terrisporobacter	1.23 ± 0.1 ^{a}	1.23 ± 0.16 ^{a}	1.2 ± 0.01 ^{a}	1.15 ± 0.05 ^{a}	0.03 ± 0.03 ^{b}	0.04 ± 0.01 ^{b}	0.03 ± 0.04 ^{b}	0.05 ± 0.01 ^{b}	0.05 ± 0.04 ^{b}	0.05 ± 0.00 ^{b}	0.04 ± 0.01 ^{b}	0.05 ± 0.02 ^{b}
Butyricicoccus	0.35 ± 0.00 ^{a}	0.34 ± 0.04 ^{a}	0.40 ± 0.03 ^{a}	0.30 ± 0.04 ^{a}	0.07 ± 0.03 ^{a}	0.08 ± 0.02 ^{a}	0.43 ± 0.15 ^{a}	0.62 ± 0.23 ^{a}	0.04 ± 0.01 ^{a}	0.04 ± 0.02 ^{a}	0.60 ± 0.14 ^{a}	1.08 ± 0.18 ^{a}
UCG-003	0.18 ± 0.02 ^{d}	0.21 ± 0.01 ^{d}	0.19 ± 0.01 ^{d}	0.19 ± 0.01 ^{d}	1.15 ± 0.07 ^{ab}	0.91 ± 0.16 ^{ab}	1.05 ± 0.06 ^{ab}	0.82 ± 0.08 ^{bc}	1.12 ± 0.11 ^{ab}	0.72 ± 0.01 ^{c}	1.28 ± 0.09 ^{a}	0.86 ± 0.01 ^{ab}
Holdemania	0.01 ± 0.01 ^{a}	0.02 ± 0.00 ^{a}	0.02 ± 0.01 ^{a}	0.02 ± 0.01 ^{a}	0.06 ± 0.01 ^{a}	0.07 ± 0.03 ^{a}	0.29 ± 0.03 ^{a}	0.30 ± 0.03 ^{a}	0.08 ± 0.01 ^{a}	0.08 ± 0.02 ^{a}	0.43 ± 0.08 ^{a}	0.40 ± 0.05 ^{a}
Intestinibacter	0.39 ± 0.20 ^{a}	0.36 ± 0.17 ^{a}	0.40 ± 0.24 ^{a}	0.27 ± 0.17 ^{a}	0.24 ± 0.12 ^{a}	0.27 ± 0.06 ^{a}	0.26 ± 0.05 ^{a}	0.18 ± 0.03 ^{a}	0.22 ± 0.03 ^{a}	0.28 ± 0.01 ^{a}	0.18 ± 0.05 ^{a}	0.21 ± 0.01 ^{a}
Epulopiscium	0.65 ± 0.06 ^{a}	0.67 ± 0.03 ^{a}	0.7 ± 0.05 ^{a}	0.69 ± 0.03 ^{a}	0.08 ± 0.03 ^{b}	0.07 ± 0.02 ^{b}	0.07 ± 0.02 ^{b}	0.06 ± 0.01 ^{b}	0.10 ± 0.01 ^{b}	0.10 ± 0.01 ^{b}	0.08 ± 0.03 ^{b}	0.09 ± 0.02 ^{b}
Veillonella	0.04 ± 0.04 ^{c}	0.06 ± 0.02 ^{c}	0.10 ± 0.01 ^{c}	0.07 ± 0.01 ^{c}	0.64 ± 0.02 ^{b}	0.68 ± 0.07 ^{b}	1.58 ± 0.07 ^{a}	0.94 ± 0.06 ^{b}	0.70 ± 0.06 ^{b}	0.73 ± 0.08 ^{b}	0.87 ± 0.01 ^{b}	0.64 ± 0.01 ^{b}
UCG-005	0.23 ± 0.04 ^{a}	0.19 ± 0.05 ^{a}	0.22 ± 0.03 ^{a}	0.23 ± 0.09 ^{a}	0.31 ± 0.02 ^{a}	0.35 ± 0.09 ^{a}	0.23 ± 0.05 ^{a}	0.30 ± 0.11 ^{a}	0.52 ± 0.1 ^{a}	0.66 ± 0.12 ^{a}	0.39 ± 0.04 ^{a}	0.54 ± 0.07 ^{a}
Colidextribacter	0.07 ± 0.01 ^{e}	0.06 ± 0.02 ^{e}	0.06 ± 0.01 ^{e}	0.07 ± 0.01 ^{e}	0.15 ± 0.04 ^{de}	0.11 ± 0.04 ^{de}	0.29 ± 0.02 ^{c}	0.67 ± 0.02 ^{a}	0.21 ± 0.02 ^{cd}	0.17 ± 0.02 ^{d}	0.46 ± 0.03 ^{b}	0.89 ± 0.05 ^{a}
Anaerofilum	nd	nd	nd	nd	0.04 ± 0.02 ^{abc}	0.02 ± 0.02 ^{bc}	0.02 ± 0.01 ^{bc}	0.03 ± 0.03 ^{abc}	0.09 ± 0.01 ^{a}	0.05 ± 0.00 ^{ab}	0.06 ± 0.01 ^{ab}	0.05 ± 0.00 ^{ab}
Flavonifractor	0.01 ± 0.02 ^{b}	0.02 ± 0.01 ^{b}	0.02 ± 0.01 ^{b}	0.02 ± 0.02 ^{b}	0.19 ± 0.06 ^{ab}	0.11 ± 0.02 ^{b}	0.08 ± 0.02 ^{b}	0.08 ± 0.01 ^{b}	0.25 ± 0.01 ^{a}	0.22 ± 0.05 ^{ab}	0.17 ± 0.05 ^{b}	0.17 ± 0.02 ^{b}
Negativibacillus	0.12 ± 0.05 ^{a}	0.17 ± 0.00 ^{a}	0.14 ± 0.04 ^{a}	0.14 ± 0.02 ^{a}	0.32 ± 0.16 ^{a}	0.22 ± 0.09 ^{a}	0.15 ± 0.04 ^{a}	0.13 ± 0.03 ^{a}	0.35 ± 0.11 ^{a}	0.37 ± 0.07 ^{a}	0.18 ± 0.07 ^{a}	0.19 ± 0.05 ^{a}
Candidatus_Soleaferrea	0.01 ± 0.01 ^{b}	nd	nd	nd	0.06 ± 0.01 ^{a}	0.04 ± 0.00 ^{b}	0.06 ± 0.02 ^{ab}	0.07 ± 0.01 ^{a}	0.09 ± 0.02 ^{a}	0.06 ± 0.00 ^{a}	0.10 ± 0.01 ^{a}	0.07 ± 0.02 ^{a}
Turicibacter	0.72 ± 0.05 ^{a}	0.7 ± 0.02 ^{a}	0.7 ± 0.06 ^{a}	0.67 ± 0.03 ^{a}	0.04 ± 0.02 ^{b}	0.05 ± 0.02 ^{b}	0.07 ± 0.01 ^{b}	0.06 ± 0.00 ^{b}	0.06 ± 0.01 ^{b}	0.06 ± 0.00 ^{b}	0.06 ± 0.02 ^{b}	0.06 ± 0.00 ^{b}
Odoribacter	0.09 ± 0.04 ^{b}	0.07 ± 0.02 ^{b}	0.11 ± 0.02 ^{b}	0.1 ± 0.00 ^{b}	0.25 ± 0.03 ^{ab}	0.26 ± 0.03 ^{ab}	0.19 ± 0.03 ^{ab}	0.17 ± 0.02 ^{b}	0.28 ± 0.05 ^{ab}	0.32 ± 0.00 ^{a}	0.18 ± 0.01 ^{b}	0.19 ± 0.01 ^{b}
Incertae_Sedis	0.11 ± 0.01 ^{b}	0.12 ± 0.02 ^{b}	0.13 ± 0.01 ^{b}	0.11 ± 0.01 ^{b}	0.10 ± 0.01 ^{b}	0.10 ± 0.01 ^{b}	0.19 ± 0.02 ^{ab}	0.19 ± 0.03 ^{ab}	0.14 ± 0.01 ^{b}	0.18 ± 0.01 ^{b}	0.20 ± 0.03 ^{ab}	0.26 ± 0.00 ^{a}
Oscillospira	nd	0.02 ± 0.02 ^{b}	0.02 ± 0.01 ^{b}	0.01 ± 0.01 ^{b}	0.09 ± 0.01 ^{ab}	0.09 ± 0.01 ^{ab}	0.13 ± 0.03 ^{ab}	0.15 ± 0.03 ^{a}	0.11 ± 0.03 ^{ab}	0.13 ± 0.01 ^{a}	0.13 ± 0.00 ^{a}	0.14 ± 0.01 ^{a}
Christensenellaceae_R-7_group	0.15 ± 0.01 ^{b}	0.12 ± 0.02 ^{b}	0.12 ± 0.02 ^{b}	0.12 ± 0.02 ^{b}	0.27 ± 0.02 ^{a}	0.27 ± 0.02 ^{a}	0.16 ± 0.03 ^{ab}	0.11 ± 0.02 ^{b}	0.3 ± 0.03 ^{a}	0.28 ± 0.03 ^{a}	0.22 ± 0.03 ^{ab}	0.23 ± 0.03 ^{ab}
Paraprevotella	0.72 ± 0.07 ^{a}	0.63 ± 0.08 ^{ab}	0.7 ± 0.11 ^{ab}	0.71 ± 0.04 ^{a}	0.01 ± 0.01 ^{d}	0.01 ± 0.01 ^{d}	0.12 ± 0.04 ^{cd}	0.18 ± 0.01 ^{bc}	nd	nd	0.01 ± 0.01 ^{d}	nd
Erysipelatoclostridium	0.06 ± 0.00 ^{b}	0.06 ± 0.01 ^{b}	0.07 ± 0.01 ^{b}	0.06 ± 0.01 ^{b}	0.07 ± 0.02 ^{b}	0.08 ± 0.02 ^{b}	0.27 ± 0.04 ^{a}	0.29 ± 0.05 ^{a}	0.06 ± 0.01 ^{b}	0.06 ± 0.01 ^{b}	0.36 ± 0.05 ^{a}	0.4 ± 0.00 ^{a}
Lachnospiraceae_FCS020_group	0.08 ± 0.02 ^{ab}	0.08 ± 0.02 ^{ab}	0.11 ± 0.01 ^{a}	0.10 ± 0.02 ^{ab}	0.08 ± 0.02 ^{ab}	0.06 ± 0.02 ^{ab}	0.04 ± 0.01 ^{b}	0.03 ± 0.02 ^{b}	nd	nd	nd	nd
CAG-56	0.18 ± 0.02 ^{a}	0.24 ± 0.03 ^{a}	0.21 ± 0.02 ^{a}	0.20 ± 0.03 ^{a}	nd	0.02 ± 0.01 ^{bc}	0.07 ± 0.01 ^{a}	0.10 ± 0.02 ^{a}	nd	nd	0.02 ± 0.01 ^{bc}	0.04 ± 0.01 ^{b}
Adlercreutzia	0.19 ± 0.02 ^{a}	0.12 ± 0.00 ^{a}	0.16 ± 0.02 ^{a}	0.17 ± 0.01 ^{a}	0.19 ± 0.06 ^{a}	0.17 ± 0.07 ^{a}	0.13 ± 0.03 ^{a}	0.11 ± 0.05 ^{a}	0.19 ± 0.08 ^{a}	0.18 ± 0.06 ^{a}	0.14 ± 0.05 ^{a}	0.12 ± 0.04 ^{a}
Lachnospiraceae_NK4A136_group	0.38 ± 0.01 ^{a}	0.34 ± 0.04 ^{a}	0.4 ± 0.04 ^{a}	0.33 ± 0.04 ^{a}	0.04 ± 0.01 ^{bc}	0.02 ± 0.02 ^{bc}	0.05 ± 0.00 ^{b}	0.04 ± 0.02 ^{bc}	0.01 ± 0.01 ^{c}	0.02 ± 0.00 ^{c}	0.02 ± 0.01 ^{bc}	0.02 ± 0.00 ^{c}
Family_XIII_AD3011_group	0.05 ± 0.00 ^{b}	0.05 ± 0.04 ^{b}	0.05 ± 0.01 ^{b}	0.05 ± 0.01 ^{b}	0.06 ± 0.04 ^{b}	0.02 ± 0.01 ^{b}	0.25 ± 0.07 ^{ab}	0.28 ± 0.02 ^{a}	0.06 ± 0.03 ^{b}	0.04 ± 0.01 ^{b}	0.32 ± 0.04 ^{a}	0.37 ± 0.02 ^{a}
Intestinimonas	nd	nd	nd	nd	nd	0.01 ± 0.01 ^{b}	0.04 ± 0.03 ^{ab}	0.02 ± 0.01 ^{b}	0.03 ± 0.01 ^{ab}	0.03 ± 0.00 ^{ab}	0.12 ± 0.02 ^{a}	0.10 ± 0.00 ^{a}
Family_XIII_UCG-001	0.05 ± 0.02 ^{a}	0.03 ± 0.01 ^{a}	0.04 ± 0.01 ^{a}	0.01 ± 0.02 ^{a}	0.11 ± 0.04 ^{a}	0.10 ± 0.01 ^{a}	0.08 ± 0.03 ^{a}	0.09 ± 0.01 ^{a}	0.23 ± 0.04 ^{a}	0.23 ± 0.02 ^{a}	0.18 ± 0.04 ^{a}	0.25 ± 0.01 ^{a}
Peptococcus	0.03 ± 0.00 ^{a}	0.013 ± 0.00 ^{a}	0.020 ± 0.00 ^{a}	0.02 ± 0.00 ^{a}	0.07 ± 0.02 ^{a}	0.04 ± 0.01 ^{a}	0.02 ± 0.01 ^{a}	0.02 ± 0.01 ^{a}	0.17 ± 0.01 ^{a}	0.29 ± 0.02 ^{a}	0.05 ± 0.01 ^{a}	0.05 ± 0.00 ^{a}

nd: not detected, SW: synthetic wine, SW+SO2: synthetic wine treated with SO_2, RW: red wine, RW+SO2: red wine treated with SO_2

Table S4. Taxa at species level during colonic fermentation at different times (0, 24, and 48 hours) for the four studied wines (SW, SW+SO2, RW, and RW+SO2) and for each volunteer (#1, #2, and #3). Data are expressed as mean relative abundance (%) ± standard deviation. Only species properly assigned to a species present in taxonomic databases and showing statistical differences in at least one case are showed. Statistically significant differences, assessed by two-way ANOVA test and Games-Howell correction ($p < 0.05$), are marked by lowercase letters. Shaded cells indicate significant differences between SO_2-treated wine and its untreated counterpart, and arrows (↑/↓) indicate the direction of change.

VOLUNTEER # 1

	0 h				24 h				48 h			
Species	SW	SW + SO2	RW	RW + SO2	SW	SW + SO2	RW	RW + SO2	SW	SW + SO2	RW	RW + SO2
Bilophila wadsworthia	0.14 ± 0.03^{c}	0.13 ± 0.01^{c}	0.11 ± 0.02^{c}	0.13 ± 0.01^{c}	5.38 ± 0.47^{bc}	6.19 ± 0.35^{b}	3.86 ± 0.25^{c}	4.3 ± 0.22^{c}	5.85 ± 1.42^{bc}	6.41 ± 0.18^{ab}	8.67 ± 0.5^{a}	7.77 ± 0.26^{a}
Bacteroides vulgatus	0.72 ± 0.09^{cd}	0.73 ± 0.08^{cd}	0.82 ± 0.02^{c}	0.91 ± 0.01^{b} (↑)	0.30 ± 0.04^{d}	0.31 ± 0.01^{d}	1.73 ± 0.06^{a}	1.47 ± 0.10^{ab}	0.74 ± 0.74^{cd}	0.11 ± 0.02^{d}	0.17 ± 0.01^{d}	0.31 ± 0.03^{d}
Coprococcus comes	0.61 ± 0.06^{d}	0.57 ± 0.08^{d}	0.60 ± 0.04^{d}	0.64 ± 0.03^{d}	4.16 ± 0.40^{cd}	6.67 ± 0.16^{b} (↑)	3.9 ± 0.43^{cd}	4.41 ± 1.25^{cd}	2.04 ± 0.99^{cd}	8.83 ± 0.08^{a} (↑)	2.04 ± 0.05^{d}	5.49 ± 0.18^{c} (↑)
Bifidobacterium longum	1.06 ± 0.11^{c}	1.09 ± 0.14^{c}	0.99 ± 0.26^{c}	1.03 ± 0.18^{c}	1.65 ± 0.07^{bc}	1.64 ± 0.14^{bc}	1.90 ± 0.18^{bc}	1.91 ± 0.22^{abc}	2.17 ± 0.43^{abc}	1.33 ± 0.21^{c}	2.74 ± 0.25^{ab}	2.77 ± 0.15^{a}
Bacteroides uniformis	4.35 ± 0.19^{a}	4.73 ± 0.29^{a}	4.43 ± 0.10^{a}	4.58 ± 0.19^{a}	2.42 ± 0.23^{a}	3.20 ± 0.38^{a}	5.06 ± 0.56^{a}	3.33 ± 0.24^{a}	1.54 ± 1.54^{ab}	3.34 ± 0.24^{a}	0.48 ± 0.02^{b}	0.37 ± 0.13^{b}
Bacteroides thetaiotaomicron	0.07 ± 0.02^{c}	0.05 ± 0.01^{c}	0.07 ± 0.01^{c}	0.07 ± 0.02^{c}	0.11 ± 0.01^{c}	0.12 ± 0.02^{c}	0.16 ± 0.04^{c}	0.41 ± 0.01^{b} (↑)	0.28 ± 0.14^{bc}	0.08 ± 0.01^{c}	0.23 ± 0.07^{bc}	0.63 ± 0.03^{a} (↑)
Parabacteroides distasonis	0.42 ± 0.08^{bc}	0.55 ± 0.05^{ab}	0.53 ± 0.04^{ab}	0.49 ± 0.06^{ab}	0.66 ± 0.04^{ab}	0.69 ± 0.05^{ab}	0.89 ± 0.04^{a}	1.03 ± 0.13^{a}	0.53 ± 0.42^{ab}	0.02 ± 0.001^{c} (↓)	0.60 ± 0.06^{ab}	0.74 ± 0.10^{ab}
Blautia obeum	0.58 ± 0.08^{ab}	0.61 ± 0.03^{a}	0.60 ± 0.07^{ab}	0.56 ± 0.06^{ab}	0.56 ± 0.06^{ab}	0.43 ± 0.03^{b}	0.50 ± 0.04^{ab}	0.52 ± 0.04^{ab}	0.53 ± 0.06^{ab}	0.22 ± 0.02^{c} (↓)	0.44 ± 0.03^{b}	0.48 ± 0.07^{ab}

VOLUNTEER # 2

	0 h				24 h				48 h			
Species	SW	SW + SO2	RW	RW + SO2	SW	SW + SO2	RW	RW + SO2	SW	SW + SO2	RW	RW + SO2
Bilophila wadsworthia	0.12 ± 0.02^{d}	0.12 ± 0.01^{d}	0.11 ± 0.02^{d}	0.1 ± 0.01^{d}	3.76 ± 0.34^{b}	3.73 ± 0.30^{b}	2.17 ± 0.16^{c}	2.27 ± 0.10^{bc}	5.94 ± 0.26^{a}	6.30 ± 0.22^{a}	3.28 ± 0.19^{b}	3.8 ± 0.32^{b}
Bacteroides coprocola	8.01 ± 0.43^{a}	8.71 ± 1.25^{a}	8.25 ± 1.19^{a}	8.88 ± 0.7^{a}	0.34 ± 0.04^{b}	0.05 ± 0.01^{c} (↓)	0.21 ± 0.07^{bc}	0.15 ± 0.03^{c}	0.08 ± 0.02^{c}	0.04 ± 0.02^{c}	0.02 ± 0.02^{c}	0.02 ± 0.001^{c}
Fusicatenibacter saccharivorans	4.50 ± 1.30^{a}	4.46 ± 0.68^{a}	4.71 ± 0.80^{a}	4.74 ± 0.76^{a}	0.34 ± 0.02^{a}	0.26 ± 0.03^{ab}	0.37 ± 0.06^{a}	0.39 ± 0.07^{a}	0.41 ± 0.03^{a}	0.32 ± 0.01^{a}	0.12 ± 0.01^{bc}	0.07 ± 0.02^{c}
Erysipelotrichaceae_UCG-003 bacterium	2.32 ± 0.37^{a}	2.22 ± 0.29^{a}	2.72 ± 0.18^{a}	2.32 ± 0.24^{a}	0.31 ± 0.03^{b}	0.06 ± 0.01^{c} (↓)	0.05 ± 0.02^{c}	0.02 ± 0.01^{c}	0.04 ± 0.01^{c}	0.00 ± 0.01^{c}	0.01 ± 0.01^{c}	nd
Ruminococcus bromii	3.59 ± 0.44^{a}	4.01 ± 0.40^{a}	3.88 ± 0.43^{a}	2.92 ± 0.32^{a}	0.38 ± 0.06^{b}	0.32 ± 0.05^{b}	0.12 ± 0.02^{c}	0.05 ± 0.01^{c}	0.39 ± 0.04^{b}	0.31 ± 0.02^{b}	0.11 ± 0.01^{c}	0.06 ± 0.01^{c}
Bacteroides thetaiotaomicron	0.09 ± 0.02^{c}	0.11 ± 0.05^{c}	0.08 ± 0.04^{c}	0.08 ± 0.01^{c}	0.17 ± 0.03^{c}	0.07 ± 0.06^{c}	0.25 ± 0.04^{c}	0.27 ± 0.01^{c}	0.35 ± 0.06^{bc}	0.17 ± 0.03^{c}	0.59 ± 0.05^{ab}	0.80 ± 0.10^{a}
Coprococcus catus	0.16 ± 0.02^{b}	0.15 ± 0.01^{b}	0.14 ± 0.02^{b}	0.16 ± 0.02^{b}	0.07 ± 0.01^{c}	0.41 ± 0.04^{a} (↑)	0.73 ± 0.08^{a}	0.46 ± 0.06^{a}	0.06 ± 0.02^{c}	0.17 ± 0.02^{b} (↑)	0.31 ± 0.01^{a}	0.36 ± 0.04^{a}
Lachnospira pectinoschiza	1.68 ± 0.19^{a}	1.55 ± 0.24^{ab}	1.46 ± 0.22^{ab}	1.67 ± 0.11^{a}	0.38 ± 0.1^{b}	0.36 ± 0.02^{b}	0.10 ± 0.02^{c}	0.09 ± 0.02^{c}	0.53 ± 0.11^{b}	0.70 ± 0.09^{b}	0.13 ± 0.02^{bc}	0.15 ± 0.03^{bc}
Alistipes putredinis	0.73 ± 0.10^{a}	0.75 ± 0.08^{a}	0.71 ± 0.09^{a}	0.78 ± 0.09^{a}	0.60 ± 0.08^{a}	0.52 ± 0.05^{a}	0.40 ± 0.06^{a}	0.35 ± 0.04^{a}	0.01 ± 0.01^{b}	0.33 ± 0.04^{a} (↑)	0.35 ± 0.02^{a}	0.28 ± 0.03^{a}
Veillonella parvula	nd	nd	nd	nd	0.20 ± 0.02^{b}	0.38 ± 0.01^{a} (↑)	0.05 ± 0.04^{cd}	0.04 ± 0.03^{d}	0.12 ± 0.01^{c}	0.32 ± 0.04^{ab} (↑)	0.04 ± 0.01^{d}	0.01 ± 0.02^{d}
Bifidobacterium breve	nd	nd	0.01 ± 0.02^{c}	0.01 ± 0.01^{c}	0.07 ± 0.02^{bc}	0.1 ± 0.001^{b}	0.39 ± 0.08^{a}	0.38 ± 0.04^{a}	0.07 ± 0.03^{bc}	0.15 ± 0.02^{b}	0.43 ± 0.01^{a}	0.50 ± 0.04^{a}
Erysipelatoclostridium ramosum	0.04 ± 0.02^{c}	0.04 ± 0.01^{c}	0.03 ± 0.02^{c}	0.03 ± 0.01^{c}	0.04 ± 0.01^{c}	0.03 ± 0.01^{c}	0.37 ± 0.03^{a}	0.28 ± 0.02^{ab}	0.03 ± 0.01^{c}	0.05 ± 0.02^{c}	0.27 ± 0.01^{ab}	0.23 ± 0.01^{b}

VOLUNTEER # 3

	0 h				24 h				48 h			
Species	SW	SW + SO2	RW	RW + SO2	SW	SW + SO2	RW	RW + SO2	SW	SW + SO2	RW	RW + SO2
Bilophila wadsworthia	0.04 ± 0.03^{d}	0.08 ± 0.02^{d}	0.04 ± 0.04^{d}	0.06 ± 0.06^{d}	3.79 ± 0.02^{b}	3.45 ± 0.31^{bc}	2.12 ± 0.16^{c}	2.13 ± 0.10^{c}	6.72 ± 0.38^{a}	6.43 ± 0.51^{a}	3.80 ± 0.22^{b}	3.72 ± 0.10^{b}
Bacteroides vulgatus	5.14 ± 0.35^{c}	5.15 ± 0.34^{c}	5.2 ± 0.37^{c}	5.49 ± 0.31^{c}	8.49 ± 0.36^{ab}	8.19 ± 0.8^{ab}	10.14 ± 0.76^{a}	9.49 ± 0.42^{ab}	5.08 ± 0.46^{c}	3.65 ± 0.41^{c}	6.49 ± 0.72^{bc}	8.06 ± 0.51^{ab}
Dorea longicatena	1.40 ± 0.13^{b}	1.53 ± 0.15^{b}	1.43 ± 0.25^{b}	1.35 ± 0.1^{b}	3.34 ± 0.3^{a}	6.73 ± 0.81^{a}	4.76 ± 0.18^{a}	4.98 ± 0.26^{a}	0.08 ± 0.01^{d}	0.32 ± 0.02^{c} (↑)	0.16 ± 0.03^{d}	0.13 ± 0.01^{d}
Blautia massiliensis	1.47 ± 0.28^{bc}	1.52 ± 0.16^{bc}	1.45 ± 0.2^{bc}	1.30 ± 0.06^{bc}	1.58 ± 0.06^{b}	1.36 ± 0.04^{bc}	0.96 ± 0.01^{c}	0.91 ± 0.05^{c}	2.09 ± 0.06^{a}	1.99 ± 0.07^{a}	1.10 ± 0.08^{c}	1.04 ± 0.02^{c}
Coprococcus comes	0.80 ± 0.08^{b}	0.75 ± 0.04^{b}	0.67 ± 0.08^{b}	0.73 ± 0.02^{b}	2.36 ± 0.6^{a}	2.80 ± 0.30^{a}	0.30 ± 0.06^{cd}	0.82 ± 0.02^{b} (↑)	0.55 ± 0.05^{bc}	0.55 ± 0.03^{bc}	0.15 ± 0.02^{d}	0.47 ± 0.06^{c} (↑)
Bifidobacterium bifidum	0.68 ± 0.06^{e}	0.72 ± 0.03^{e}	0.77 ± 0.06^{de}	0.86 ± 0.06^{de}	0.74 ± 0.05^{e}	0.64 ± 0.05^{e}	1.95 ± 0.08^{ab}	1.88 ± 0.08^{b}	1.16 ± 0.02^{c}	1.04 ± 0.01^{d} (↓)	2.79 ± 0.25^{ab}	2.82 ± 0.19^{a}
Acidaminococcus intestini	1.00 ± 0.15^{d}	1.03 ± 0.17^{d}	1.06 ± 0.05^{d}	1.12 ± 0.06^{d}	5.02 ± 0.25^{ab}	4.81 ± 0.35^{abc}	3.33 ± 0.24^{c}	0.93 ± 0.07^{d} (↓)	5.78 ± 0.23^{a}	5.33 ± 0.26^{a}	4.02 ± 0.26^{bc}	0.26 ± 0.04^{e} (↓)
Bacteroides uniformis	1.5 ± 0.02^{c}	1.32 ± 0.07^{c}	1.54 ± 0.07^{c}	1.60 ± 0.18^{c}	3.55 ± 0.12^{a}	2.85 ± 0.04^{b} (↓)	3.23 ± 0.19^{ab}	3.29 ± 0.1^{ab}	3.03 ± 0.08^{b}	0.88 ± 0.01^{c} (↓)	3.56 ± 0.09^{a}	3.54 ± 0.1^{a}
Ruminococcus bromii	3.15 ± 0.24^{a}	3.25 ± 0.1^{a}	3.15 ± 0.07^{a}	3.07 ± 0.17^{a}	1.50 ± 0.05^{b}	1.42 ± 0.09^{b}	0.82 ± 0.05^{bc}	0.79 ± 0.03^{bc}	1.55 ± 0.16^{b}	1.40 ± 0.01^{b}	0.62 ± 0.04^{c}	0.61 ± 0.05^{c}
Coprococcus catus	0.19 ± 0.02^{d}	0.21 ± 0.03^{d}	0.18 ± 0.03^{d}	0.18 ± 0.02^{d}	0.25 ± 0.02^{d}	0.14 ± 0.01^{d}	0.67 ± 0.05^{ab}	0.89 ± 0.07^{a}	0.32 ± 0.07^{cd}	0.12 ± 0.01^{d}	0.56 ± 0.06^{bc}	0.48 ± 0.02^{bc}
Faecalicoccus pleomorphus	0.05 ± 0.01^{c}	0.07 ± 0.03^{c}	0.06 ± 0.01^{c}	0.08 ± 0.01^{c}	0.17 ± 0.01^{b}	0.15 ± 0.04^{bc}	2.12 ± 0.31^{a}	2.66 ± 0.24^{a}	0.14 ± 0.05^{bc}	0.13 ± 0.04^{bc}	2.77 ± 0.11^{a}	2.73 ± 0.46^{a}
Parabacteroides distasonis	0.19 ± 0.02^{f}	0.22 ± 0.05^{f}	0.19 ± 0.01^{f}	0.23 ± 0.03^{f}	1.13 ± 0.06^{cd}	0.94 ± 0.08^{de}	1.67 ± 0.07^{b}	1.55 ± 0.13^{bc}	2.06 ± 0.08^{a}	1.87 ± 0.14^{ab}	0.84 ± 0.02^{e}	0.97 ± 0.01^{cd} (↑)
Alistipes finegoldii	0.01 ± 0.02^{cd}	0.01 ± 0.02^{bcd}	nd	nd	0.13 ± 0.02^{a}	0.11 ± 0.01^{a}	0.08 ± 0.01^{a}	0.07 ± 0.01^{abc}	0.08 ± 0.01^{ab}	0.01 ± 0.01^{d} (↓)	0.08 ± 0.01^{a}	0.11 ± 0.01^{a}
Ruminococcus bicirculans	1.26 ± 0.05^{a}	1.41 ± 0.18^{a}	1.23 ± 0.03^{a}	1.17 ± 0.12^{a}	0.12 ± 0.02^{bc}	0.15 ± 0.04^{bc}	0.06 ± 0.03^{bc}	0.10 ± 0.03^{bc}	0.05 ± 0.01^{c}	0.16 ± 0.01^{b} (↑)	0.04 ± 0.01^{c}	0.10 ± 0.02^{bc}
Phocea massiliensis	nd	nd	nd	nd	0.50 ± 0.06^{abc}	0.49 ± 0.03^{bc}	0.40 ± 0.03^{c}	0.26 ± 0.02^{c}	0.77 ± 0.06^{ab}	0.78 ± 0.05^{a}	0.59 ± 0.04^{ab}	0.46 ± 0.07^{bc}
Colidextribacter massiliensis	0.05 ± 0.001^{d}	0.04 ± 0.01^{d}	0.04 ± 0.01^{d}	0.03 ± 0.01^{d}	0.03 ± 0.03^{d}	0.03 ± 0.03^{d}	0.18 ± 0.01^{b}	0.47 ± 0.02^{a} (↑)	0.07 ± 0.02^{cd}	0.07 ± 0.01^{c}	0.29 ± 0.02^{a}	0.70 ± 0.08^{a}

nd: not detected, SW: synthetic wine, SW+SO2: synthetic wine treated with SO_2, RW: red wine, RW+SO2: red wine treated with SO_2

Table S5. Concentration (µM) of short-chain fatty acids (SCFAs) during colonic fermentation at different times (0, 24, and 48 hours) for the four studied wines (SW, SW+SO2, RW, and RW+SO2) and for each volunteer (#1, #2, and #3). Statistically significant differences, assessed by two-way ANOVA test and Games-Howell correction ($p < 0.05$), are marked by lowercase letters. Shaded cells indicate significant differences between SO_2-treated wine and its untreated counterpart, and arrows (↑/↓) indicate the direction of change.

VOLUNTEER # 1

	0 h				24 h				48 h			
Short-chain fatty acid	SW	SW + SO2	RW	RW + SO2	SW	SW + SO2	RW	RW + SO2	SW	SW + SO2	RW	RW + SO2
Acetic acid	1536.42 ± 16.1 ^{h}	1660.21 ± 17.06 ^{g} (↑)	2232.43 ± 12.17 ^{f}	2469.88 ± 36.26 ^{e} (↑)	11202.01 ± 115.63 ^{d}	11574.29 ± 241.01 ^{cd}	14514.26 ± 95.1 ^{b}	14520.94 ± 365.36 ^{b}	12806.22 ± 8.84 ^{c}	19455.89 ± 766.61 ^{a} (↑)	17818.21 ± 256.21 ^{a}	15734.08 ± 274.66 ^{ab}
Propionic acid	444.2 ± 15.38 ^{e}	433.29 ± 9.24 ^{e}	757.44 ± 1.64 ^{d}	906.07 ± 30.93 ^{d}	2392.97 ± 9.6 ^{c}	2507.2 ± 49.86 ^{c}	5246.88 ± 44.34 ^{b}	5082.94 ± 103.51 ^{b}	2691.96 ± 0.48 ^{c}	3121.85 ± 145.73 ^{c}	6214.74 ± 83.24 ^{a}	5333.98 ± 78.2 ^{b} (↓)
Isobutyric acid	34.3 ± 1.92 ^{ef}	24.73 ± 0.59 ^{f}	41.27 ± 0.7 ^{de}	45.41 ± 2.04 ^{d}	154.34 ± 10.56 ^{c}	154.97 ± 9.53 ^{c}	250.09 ± 9.63 ^{b}	251.32 ± 11.57 ^{b}	188.27 ± 11.59 ^{c}	211.39 ± 0.5 ^{bc}	356.29 ± 8.09 ^{a}	349.35 ± 9.96 ^{a}
Butyric acid	218.25 ± 20.2 ^{h}	216.11 ± 4.65 ^{h}	226.86 ± 12.88 ^{h}	233.12 ± 2.17 ^{h}	2009.58 ± 75.51 ^{g}	2796.12 ± 5.58 ^{e} (↑)	5142.94 ± 6.46 ^{c}	5317.33 ± 37.2 ^{c}	2547.09 ± 24.1 ^{f}	4086.13 ± 122.3 ^{d} (↑)	6976.91 ± 26.93 ^{a}	6405.63 ± 43.8 ^{b} (↓)
Isovaleric acid	37.24 ± 5.81 ^{f}	42.4 ± 1.51 ^{f}	43.27 ± 1.88 ^{f}	44.82 ± 3.48 ^{f}	286.53 ± 4.9 ^{e}	304.27 ± 8.41 ^{e}	402.95 ± 1.3 ^{c}	403.8 ± 2.95 ^{c}	354.29 ± 21.83 ^{de}	382.67 ± 0.95 ^{d}	580.52 ± 6.05 ^{a}	541.05 ± 0.99 ^{b} (↓)
Valeric acid	41.12 ± 3.62 ^{e}	50.47 ± 3.08 ^{e}	42.68 ± 0.17 ^{e}	48.24 ± 1.98 ^{e}	860.99 ± 5.94 ^{d}	872.96 ± 5.82 ^{d}	1277.97 ± 7.94 ^{c}	1277.26 ± 10.02 ^{c}	966.01 ± 17.68 ^{d}	990.23 ± 25.71 ^{d}	1515.35 ± 5.63 ^{a}	1331.56 ± 1.03 ^{b} (↓)
Caproic acid	nd	nd	nd	nd	nd	nd	nd	nd	nd	1087.38 ± 47.65 ^{a}	48.59 ± 2.8 ^{b}	nd
Heptanoic acid	nd	nd	nd	nd	nd	nd	nd	nd	nd	nd	48.99 ± 6.06 ^{a}	53.52 ± 2.87 ^{a}

VOLUNTEER # 2

	0 h				24 h				48 h			
Short-chain fatty acid	SW	SW + SO2	RW	RW + SO2	SW	SW + SO2	RW	RW + SO2	SW	SW + SO2	RW	RW + SO2
Acetic acid	1349.89 ± 3.8 ^{f}	1319.4 ± 25.03 ^{f}	1666.16 ± 32.59 ^{e}	1789.85 ± 35.95 ^{e}	10006.31 ± 137.67 ^{d}	10382.4 ± 19.57 ^{d}	13513.88 ± 422.41 ^{bc}	14280.64 ± 108.07 ^{b}	11317.68 ± 71.32 ^{c}	11288.52 ± 52.03 ^{c}	14542.13 ± 51.72 ^{b}	15488.33 ± 156.98 ^{a} (↑)
Propionic acid	292.22 ± 4.02 ^{g}	277.91 ± 4.25 ^{g}	324.57 ± 12.43 ^{fg}	343.97 ± 0.68 ^{f}	2292.64 ± 16.94 ^{d}	2151.73 ± 4.59 ^{e} (↓)	5366.69 ± 157.75 ^{b}	5942.41 ± 122.38 ^{ab}	2538.39 ± 0.93 ^{c}	2265.76 ± 20.61 ^{de} (↓)	5559.72 ± 99.22 ^{b}	6186.24 ± 84.2 ^{a} (↑)
Isobutyric acid	17.37 ± 3.62 ^{e}	18.4 ± 1.14 ^{e}	30.09 ± 6.06 ^{e}	29.04 ± 2.41 ^{e}	138.17 ± 9.72 ^{c}	64.68 ± 5.64 ^{d} (↓)	256.06 ± 4.01 ^{b}	237.34 ± 7.29 ^{b}	261.71 ± 7.27 ^{b}	93.68 ± 1.56 ^{cd} (↓)	452.49 ± 2.29 ^{a}	453.81 ± 6.48 ^{a}
Butyric acid	450.79 ± 13.49 ^{e}	435.75 ± 28.87 ^{e}	451.59 ± 9.93 ^{e}	460.19 ± 32.94 ^{e}	1732.02 ± 2.87 ^{d}	1759.52 ± 6.08 ^{d}	3281.13 ± 112.47 ^{b}	3376.1 ± 54.39 ^{b}	2126.76 ± 8.26 ^{c}	1939.36 ± 41.37 ^{cd}	3749.68 ± 36.95 ^{a}	3852.26 ± 45.82 ^{a}
Isovaleric acd	33.14 ± 1.46 ^{g}	26.93 ± 2.33 ^{g}	28.96 ± 0.4 ^{g}	30.49 ± 1.89 ^{g}	340.12 ± 11.03 ^{d}	141.68 ± 6.7 ^{f} (↓)	454.35 ± 7.54 ^{c}	371.37 ± 20.61 ^{cd}	540.42 ± 13.58 ^{b}	200.89 ± 5.21 ^{e} (↓)	631.83 ± 5.65 ^{a}	596.97 ± 21.77 ^{ab}
Valeric acid	27.14 ± 3.5 ^{ef}	24.56 ± 3.25 ^{f}	32.55 ± 0.59 ^{def}	29.35 ± 2.75 ^{ef}	62.88 ± 6.56 ^{cd}	41.77 ± 6.14 ^{def}	84.34 ± 8.06 ^{bc}	106.08 ± 1.71 ^{ab}	84.37 ± 7.16 ^{bc}	44.8 ± 4.37 ^{de} (↓)	121.06 ± 6.13 ^{a}	145.73 ± 10.45 ^{a}
Heptanoic acid	nd	nd	nd	nd	nd	nd	38.1 ± 5.49 ^{a}	55.9 ± 13.6 ^{a}	nd	nd	44.12 ± 2.85 ^{a}	49.04 ± 1.2 ^{a}

VOLUNTEER # 3

	0 h				24 h				48h			
Short-chain fatty acid	SW	SW + SO2	RW	RW + SO2	SW	SW + SO2	RW	RW + SO2	SW	SW + SO2	RW	RW + SO2
Acetic acid	1588.45 ± 45.05 ^{f}	1647.19 ± 92.27 ^{ef}	1883.07 ± 43.77 ^{e}	2067.8 ± 32.34 ^{e}	12301.2 ± 1.7 ^{d}	12464.72 ± 20.11 ^{d}	18007.93 ± 257.39 ^{b}	19869.68 ± 109.42 ^{a} (↑)	14131.68 ± 44.59 ^{c}	13939.45 ± 546.18 ^{cd}	18838.15 ± 503.35 ^{ab}	20623.52 ± 457.26 ^{a}
Propionic acid	311.15 ± 8.13 ^{f}	312.66 ± 0.73 ^{f}	335.71 ± 0.47 ^{e}	374.02 ± 2.99 ^{d} (↑)	2563.04 ± 19.42 ^{c}	2609.27 ± 23.66 ^{c}	4722.39 ± 28.65 ^{b}	5260.03 ± 4.51 ^{a} (↑)	2917.83 ± 67.77 ^{c}	2744.45 ± 57.24 ^{c}	4757.39 ± 149.15 ^{ab}	5219.28 ± 100.09 ^{ab}
Isobutyric acid	26.79 ± 3.53 ^{c}	27.43 ± 4.5 ^{c}	31.2 ± 0.65 ^{c}	37.67 ± 2.03 ^{c}	495.81 ± 21.93 ^{b}	501.38 ± 15.97 ^{ab}	497.87 ± 2.63 ^{b}	535 ± 19.37 ^{ab}	579.15 ± 2.27 ^{a}	541.25 ± 22.64 ^{ab}	563.85 ± 3.54 ^{a}	569.25 ± 1.84 ^{a}
Butyric acid	293.36 ± 4.04 ^{f}	295.62 ± 11.23 ^{f}	285.47 ± 11.92 ^{f}	292.64 ± 15.06 ^{f}	1928.14 ± 8.23 ^{e}	2003.03 ± 58.08 ^{de}	2485.88 ± 30.91 ^{c}	2910.65 ± 19.52 ^{b} (↑)	2217.28 ± 20.43 ^{d}	2279.46 ± 11.68 ^{d}	2739.19 ± 53.73 ^{b}	3075.73 ± 14.45 ^{a} (↑)
Isovaleric acid	30.34 ± 1.22 ^{d}	30.17 ± 1.23 ^{d}	33.54 ± 0.75 ^{d}	32.19 ± 0.76 ^{d}	784.43 ± 12.46 ^{abc}	788.03 ± 17.19 ^{ab}	617.08 ± 34.03 ^{c}	654.31 ± 29.39 ^{c}	908.72 ± 29.35 ^{a}	874.51 ± 24.64 ^{a}	738.44 ± 39.21 ^{abc}	713.67 ± 29.32 ^{bc}
Valeric acid	23.59 ± 2.48 ^{gh}	24.35 ± 3.54 ^{gh}	14.19 ± 1.16 ^{h}	27.7 ± 2.07 ^{g} (↑)	455.36 ± 2.69 ^{e}	407.43 ± 1.3 ^{f} (↓)	1092.24 ± 5.59 ^{b}	1163.74 ± 4.99 ^{a} (↑)	822.93 ± 18.85 ^{c}	673.63 ± 4 ^{d} (↓)	1158.48 ± 9.79 ^{a}	1213.16 ± 21.46 ^{a}
Caproic acid	nd	nd	nd	nd	66.86 ± 3.91 ^{d}	66.14 ± 9.18 ^{d}	187.04 ± 13.63 ^{c}	326.09 ± 18.23 ^{b} (↑)	114.11 ± 2.56 ^{c}	72.95 ± 0.91 ^{d} (↓)	312.32 ± 8.7 ^{b}	512.14 ± 18.19 ^{a} (↑)
Heptanoic acid	nd	nd	nd	nd	nd	nd	48.92 ± 2.86 ^{c}	111.92 ± 14.34 ^{bc}	nd	nd	115.4 ± 1.91 ^{b}	230.99 ± 11.86 ^{a} (↑)

nd: not detected, SW: synthetic wine, SW+SO2: synthetic wine treated with SO_2, RW: red wine, RW+SO2: red wine treated with SO_2

Table S6. Concentration (µM) of ammonium (NH_4^+) during colonic fermentation at different times (0, 24, and 48 hours) for the four studied wines (SW, SW+SO2, RW, and RW+SO2) and for each volunteer (#1, #2, and #3). Statistically significant differences, assessed by two-way ANOVA test with Tukey post hoc correction ($p < 0.05$), are marked by lowercase letters. Shaded cells indicate significant differences between SO_2-treated wine and its untreated counterpart, and arrows (↑/↓) indicate the direction of change.

VOLUNTEER # 1												
	0 h				24 h				48 h			
	SW	SW + SO2	RW	RW + SO2	SW	SW + SO2	RW	RW + SO2	SW	SW + SO2	RW	RW + SO2
Ammonium ion (NH_4^+)	28.94 ± 1.71 ^{f}	31.92 ± 0.47 ^{f}	31.86 ± 1.1 ^{f}	33.01 ± 0.22 ^{f}	187.4 ± 3.04 ^{e}	192.12 ± 2.86 ^{de}	197.14 ± 2.86 ^{cd}	193 ± 3.97 ^{de}	220.57 ± 1.16 ^{b}	199.37 ± 0.6 ^{c} (↓)	247.86 ± 2.35 ^{a}	203.01 ± 1.11 ^{c} (↓)
VOLUNTEER # 2												
	0 h				24 h				48 h			
	SW	SW + SO2	RW	RW + SO2	SW	SW + SO2	RW	RW + SO2	SW	SW + SO2	RW	RW + SO2
Ammonium ion (NH_4^+)	24.09 ± 0 ^{g}	24.23 ± 0.51 ^{g}	23.9 ± 0.36 ^{g}	20.72 ± 0.16 ^{g}	126.02 ± 1.21 ^{d}	136.16 ± 0.98 ^{c} (↑)	113.06 ± 0.43 ^{e}	108.84 ± 2.48 ^{f} (↓)	163.52 ± 1.23 ^{a}	148.07 ± 1.29 ^{b} (↓)	149.07 ± 1.96 ^{b}	150.47 ± 1.98 ^{b}
VOLUNTEER # 3												
	0 h				24 h				48 h			
	SW	SW + SO2	RW	RW + SO2	SW	SW + SO2	RW	RW + SO2	SW	SW + SO2	RW	RW + SO2
Ammonium ion (NH_4^+)	28.89 ± 0.32 ^{g}	28.59 ± 1.15 ^{g}	31.11 ± 0.04 ^{g}	27.71 ± 0.68 ^{g}	174.61 ± 1.72 ^{f}	201.64 ± 5.37 ^{d} (↑)	188.03 ± 1.74 ^{e}	191.01 ± 0.11 ^{e}	228.6 ± 0.43 ^{a}	220.18 ± 5.25 ^{b} (↓)	212.51 ± 1.59 ^{c}	201.11 ± 1.5 ^{d} (↓)

SW: synthetic wine, SW+SO2: synthetic wine treated with SO_2, RW: red wine, RW+SO2: red wine treated with SO_2

Table S7. Concentration of phenolic compounds in red wines (RW and RW+SO2) and their corresponding gastrointestinal digests. Data are expressed as mean values (n=3) ± standard deviation. The Kruskal-Wallis test was used to test for significant differences. Arrows (↑/↓) indicate the direction of change.

Compound (mg/L)	RW	RW+SO2	RW digest	RW+SO2 digest
Ethyl gallate	1.09 ± 0.05	1.08 ± 0.12	nd	nd
Gallic acid	17.2 ± 1.54*	13.8 ± 1.25* (↓)	0.12 ± 0.02	0.13 ± 0.02
Protocatechuic acid	1.06 ± 0.07	1.03 ± 0.17	0.17 ± 0.03*	0.20 ± 0.01* (↑)
Vanillic acid	8.41 ± 0.52	8.11 ± 1.39	3.25 ± 0.21*	3.71 ± 0.14* (↑)
Syringic acid	4.11 ± 0.26	4.03 ± 0.63	1.59 ± 0.24	1.71 ± 0.30
Ellagic acid	3.71 ± 1.81	3.78 ± 0.24	nd	nd
∑ Benzoic acids	35.9 ± 4.3	32.1 ± 3.8	5.24 ± 0.51	5.85 ± 0.48
Cumaric acid	0.89 ± 0.09	0.81 ± 0.11	0.29 ± 0.02	0.29 ± 0.03
Coutaric acid	3.57 ± 0.28	3.34 ± 0.44	0.35 ± 0.03*	0.37 ± 0.03* (↑)
p-Coumaroyl hexose isomer 1	1.31 ± 0.06	1.21 ± 0.10	0.201 ± 0.011	0.172 ± 0.01
p-Coumaroyl hexose isomer 2	0.301 ± 0.011*	0.265 ± 0.031* (↓)	0.042 ± 0.006	0.041 ± 0.002
Caffeic acid	0.621 ± 0.060	0.591 ± 0.120	0.11 ± 0.02	0.1 ± 0.01
Caffaric acid	0.161 ± 0.010	0.181 ± 0.030	nd	nd
3-O-ß-D-Glucosyl-trans-caffeic acid	1.56 ± 0.06	1.21 ± 0.20	0.111 ± 0.021	0.090 ± 0.010
Ferulic acid	0.231 ± 0.050	0.211 ± 0.060	nd	nd
∑ Hydroxycinnamic acids and derivates	8.64 ± 0.62	7.80 ± 1.09	1.13 ± 0.11	1.09 ± 0.11
Resveratrol	6.93 ± 0.72	6.69 ± 0.66	1.0 ± 0.4	1.13 ± 0.09
Piceid	1.2 ± 0.06	1.11 ± 0.1	0.17 ± 0.03	0.16 ± 0.03
∑ Stilbenes	8.13 ± 0.78	7.8 ± 0.80	1.16 ± 0.42	1.29 ± 0.12
Kaempferol-3-O-galactoside	0.19 ± 0.01	0.2 ± 0.02	nd	nd
Quercetin	0.83 ± 0.02	0.82 ± 0.09	nd	nd
Quercetin-3-O-galactoside	10.0 ± 0.3	9.5 ± 1.16	1.85 ± 0.07	1.88 ± 0.22
Quercetin-3-O-glucoside	5.09 ± 0.24	5.48 ± 0.34	1.51 ± 0.08	1.49 ± 0.07
∑ Flavonols	16.1 ± 0.5	16.0 ± 1.6	3.36 ± 0.15	3.37 ± 0.29
(+)-Catechin	15.0 ± 0.8	13.91 ± 1.6	0.131 ± 0.061*	0.611 ± 0.110* (↑)
(-)-Epicatechin	6.38 ± 0.45	6.54 ± 1.02	nd	0.101 ± 0.030* (↑)
(-)-Epigallocatechin	0.372 ± 0.010	0.411 ± 0.051	nd	nd
Procyanidin B1 + procyanidin B3	29.1 ± 4.5	26.4 ± 2.5	nd	nd
Procyanidin B2	10.5 ± 0.7	10.2 ± 0.6	0.531 ± 0.090*	1.32 ± 0.05* (↑)
Procyanidin C1	17.5 ± 0.2	17.4 ± 1.1	nd	nd
Procyanidin B gallates	1.66 ± 0.01*	1.81 ± 0.02* (↑)	nd	nd
∑ Flavan-3-ols	80.5 ± 6.6	76.8 ± 6.9	0.662 ± 0.151*	2.03 ± 0.19* (↑)
Malvidin-3-O-glucoside	713 ± 7*	807 ± 13* (↑)	0.221 ± 0.011*	1.15 ±0.03* (↑)
Peonidin-3-O-glucoside	75.7 ± 0.1*	85.4 ± 2.8* (↑)	0.0311± 0.0011*	0.250 ± 0.040* (↑)
Cyanidin-3-O-glucoside	0.521 ± 0.021	0.410 ± 0.130	nd	nd
Delphinidin-3-O-glucoside	20.6 ± 0.9*	14.7 ± 0.8* (↓)	nd	nd
Petunidin-3-O-glucoside	51.2 ± 3.0*	64.0 ± 0.7* (↑)	nd	nd
Malvidin-3-O-(6′-acetyl)glucoside	74.6 ± 3.4*	94.0 ± 0.2* (↑)	nd	nd
Peonidin-3-O-(6′-acetyl)glucoside	27.8 ± 0.1*	32.8 ± 0.4* (↑)	nd	nd
Cyanidin-3-O-(6′-acetyl)glucoside	0.421 ± 0.003*	0.521 ± 0.061* (↑)	nd	nd
Delphinidin-3-O-(6′-acetyl)glucoside	1.67 ± 0.03	1.61 ± 0.11	nd	nd
Petunidin-3-O-(6′-acetyl)glucoside	0.221 ± 0.291*	0.942 ± 0.122* (↑)	nd	nd

Malvidin-3-O-(6´-p-coumaroyl)glucoside	104 ± 6	109 ± 2	0.140 ± 0.002*	0.941 ± 0.070* (↑)
Peonidin-3-O-(6´-p-coumaroyl)glucoside	56.0 ± 3.6*	63.0 ± 1.0* (↑)	0.131 ± 0.021*	0.540 ± 0.021* (↑)
Cyanidin-3-O-(6´-p-coumaroyl)glucoside	1.51 ± 0.08*	1.32 ± 0.01* (↓)	nd	nd
Delphinidin-3-O-(6´-p-coumaroyl)glucoside	1.09 ± 0.48*	1.57 ± 0.16* (↑)	nd	nd
Petunidin-3-O-(6´-p-coumaroyl)glucoside	3.79 ± 1.08	4.43 ± 0.35	nd	nd
Malvidin-3-O-(6´-cafeoyl)glucoside	1.68 ± 0.04*	2.38 ± 0.12* (↑)	nd	nd
Peonidin-3-O-(6´-cafeoyl)glucoside	1.02 ± 0.09	0.95 ± 0.05	nd	nd
Petunidin-3-O-(6´-cafeoyl)glucoside	0.292 ± 0.012*	0.172 ± 0.001* (↓)	nd	nd
Malvidin-3-O-glucoside-pyruvate	9.75 ± 1.35*	7.07 ± 0.71* (↓)	1.23 ± 0.13*	1.59 ± 0.03* (↑)
Peonidin-3-O-glucoside-pyruvate	0.831 ± 0.030*	0.870 ± 0.011* (↑)	0.190 ± 0.041	0.201 ± 0.031
Petunidin-3-O-glucoside-pyruvate	0.350 ± 0.030	0.321 ± 0.020	nd	nd
∑ Anthocyanins	1146 ± 27*	1292 ± 23* (↑)	1.94 ± 0.21*	4.67 ± 0.22* (↑)

nd: not detected, SW: synthetic wine, SW+SO2: synthetic wine treated with SO_2, RW: red wine, RW+SO2: red wine treated with SO_2, * indicates statistical significance ($p < 0.05$) between SO_2-treated wine and its untreated counterpart

Table S8. Concentration of wine phenolic metabolites during colonic fermentation at different times (0, 6, 24, and 48 hours) for the red wines (RW and RW+SO2) and for each volunteer (#1, #2, and #3). Data are expressed as mean values (n=3) ±standard deviation. Statistically significant differences, assessed by two-way ANOVA test with Tukey post hoc correction ($p < 0.05$), are marked by lowercase letters. Shaded cells indicate significant differences between SO2-treated wine and its untreated counterpart, and arrows (↑/↓) indicate the direction of change.

VOLUNTEER # 1

	0 h		6 h		24 h		48 h	
Compound (mg/L)	RW	RW + SO2	RW	RW + SO2	RW	RW + SO2	RW	RW + SO2
4-Hydroxybenzoic acid	0.37 ± 0.07 ^{b}	0.41 ± 0.03 ^{b}	0.41 ± 0.01 ^{b}	0.4 ± 0.01 ^{b}	0.44 ± 0.01 ^{b}	0.52 ± 0.00 ^{a} (↑)	0.53 ± 0.01 ^{a}	0.47 ± 0.04 ^{ab}
Salicylic acid	nd	nd	nd	nd	nd	0.03 ± 0.01 ^{a}	0.03 ± 0.01 ^{a}	0.02 ± 0.01 ^{a}
Protocatechuic acid	0.10 ± 0.01 ^{c}	0.19 ± 0.02 ^{ab} (↑)	0.17 ± 0.01 ^{ab}	0.18 ± 0.05 ^{ab}	0.13 ± 0.01 ^{bc}	0.21 ± 0.01 ^{a} (↑)	0.12 ± 0.01 ^{bc}	0.14 ± 0.02 ^{bc}
Vanillic acid	2.31 ± 0.23 ^{bc}	2.70 ± 0.17 ^{ab}	2.00 ± 0.06 ^{c}	2.35 ± 0.29 ^{ab} (↑)	2.34 ± 0.01 ^{b}	2.83 ± 0.17 ^{ab}	2.48 ± 0.62 ^{ab}	3.33 ± 0.18 ^{a}
4-O-Methylgallic acid	nd	nd	0.02 ± 0.01 ^{a}	0.03 ± 0.01 ^{a}	nd	nd	nd	nd
Syringic acid	1.31 ± 0.06 ^{a}	2.11 ± 0.21 ^{a}	1.42 ± 0.01 ^{a}	2.03 ± 0.32 ^{a}	0.71 ± 0.07 ^{bc}	0.75 ± 0.02 ^{b}	0.82 ± 0.06 ^{b}	0.52 ± 0.05 ^{c} (↓)
p-Coumaric acid	0.27 ± 0.01 ^{b}	0.36 ± 0.02 ^{a} (↑)	nd	nd	nd	nd	nd	nd
3-Hydroxyphenylacetic acid	0.02 ± 0.01 ^{b}	0.01 ± 0.01 ^{b}	0.07 ± 0.02 ^{b}	0.03 ± 0.02 ^{b}	0.09 ± 0.01 ^{ab}	0.20 ± 0.01 ^{a}	0.20 ± 0.08 ^{ab}	0.25 ± 0.03 ^{a}
4-Hydroxyphenylacetic acid	0.84 ± 0.04 ^{c}	0.97 ± 0.16 ^{c}	3.63 ± 0.36 ^{b}	3.35 ± 0.72 ^{bc}	5.31 ± 0.16 ^{a}	5.77 ± 0.38 ^{a}	6.85 ± 0.68 ^{a}	5.98 ± 0.34 ^{a}
3,4-Dihydroxyphenylacetic acid	nd	nd	0.09 ± 0.01 ^{c}	0.19 ± 0.01 ^{a} (↑)	0.05 ± 0.01 ^{d}	0.12 ± 0.01 ^{b} (↑)	0.09 ± 0.02 ^{cd}	0.07 ± 0.01 ^{d}
Phenylacetic acid	nd	nd	12.11 ± 0.91 ^{b}	15.98 ± 0.35 ^{b}	25.32 ± 0.49 ^{a}	27.77 ± 1.96 ^{a}	32.92 ± 3.03 ^{a}	28.49 ± 6.40 ^{a}
3-(3´-Hydroxyphenyl)propionic acid	0.07 ± 0.00 ^{b}	0.14 ± 0.01 ^{b}	0.29 ± 0.04 ^{ab}	0.47 ± 0.07 ^{a}	0.06 ± 0.01 ^{c}	0.07 ± 0.00 ^{bc}	0.14 ± 0.01 ^{b}	0.17 ± 0.01 ^{ab}
Phenylpropionic acid	nd	nd	26.12 ± 0.69 ^{d}	27.72 ± 0.11 ^{d}	28.86 ± 0.60 ^{d}	32.21 ± 0.32 ^{c} (↑)	35.1 ± 0.22 ^{a}	33.76 ± 0.09 ^{b} (↓)
4-hydroxy-5-(3´-hydroxyphenyl) valeric acid	nd	0.006 ± 0.001 ^{e} (↑)	nd	nd	0.07 ± 0.01 ^{d}	0.21 ± 0.00 ^{c} (↑)	0.43 ± 0.01 ^{b}	0.69 ± 0.02 ^{a} (↑)
4-hydroxy-5-(3´,4´-dihydroxyphenyl) valeric ac	nd	nd	nd	0.013± 0.01 ^{a} (↑)	0.023 ± 0.01 ^{a}	0.011 ± 0.01 ^{ab} (↓)	nd	nd
5-(3′-hydroxyphenyl)-γ-valerolactone	nd	nd	nd	0.18 ± 0.02 ^{a} (↑)	nd	nd	nd	nd
5-(3′,4′-dihydroxyphenyl)-γ-valerolactone	nd	nd	0.78 ± 0.09 ^{b}	1.28 ± 0.07 ^{a} (↑)	0.73 ± 0.08 ^{b}	0.61 ± 0.06 ^{b}	0.57 ± 0.09 ^{b}	0.32 ± 0.01 ^{b}

VOLUNTEER # 2

	0 h		6 h		24 h		48 h	
Phenolic metabolites	RW	RW + SO2	RW	RW + SO2	RW	RW + SO2	RW	RW + SO2
4-Hydroxybenzoic acid	0.59 ± 0.02 ^{b}	0.91 ± 0.23 ^{a} (↑)	0.63 ± 0.01 ^{ab}	0.94 ± 0.05 ^{a}	0.80 ± 0.04 ^{a}	0.97 ± 0.07 ^{a}	0.92 ± 0.07 ^{a}	0.92 ± 0.02 ^{a}
Salicylic acid	nd	nd	0.17 ± 0.01 ^{b}	0.26 ± 0.09 ^{ab}	0.34 ± 0.05 ^{ab}	0.48 ± 0.03 ^{a}	0.22 ± 0.02 ^{b}	0.41 ± 0.06 ^{ab}
Protocatechuic acid	0.09 ± 0.02 ^{c}	0.14 ± 0.04 ^{ab} (↑)	0.10 ± 0.02 ^{c}	0.21 ± 0.01 ^{a} (↑)	0.11 ± 0.01 ^{c}	0.21 ± 0.01 ^{a} (↑)	0.09 ± 0.03 ^{c}	0.16 ± 0.01 ^{b} (↑)
Vanillic acid	1.93 ± 0.27 ^{a}	2.07 ± 0.04 ^{a}	2.23 ± 0.06 ^{a}	3.24 ± 0.12 ^{a}	2.37 ± 0.08 ^{a}	3.08 ± 0.35 ^{a}	2.22 ± 0.01 ^{a}	3.60 ± 0.43 ^{a}
4-O-Methylgallic acid	nd	nd	0.01 ± 0.01 ^{a}	0.04 ± 0.01 ^{a}	0.02 ± 0.01 ^{a}	0.02 ± 0.01 ^{a}	0.02 ± 0.01 ^{a}	0.02 ± 0.01 ^{a}
Syringic acid	0.91 ± 0.04 ^{c}	1.63 ± 0.17 ^{bc}	1.41 ± 0.09 ^{c}	3.34 ± 0.34 ^{a} (↑)	1.58 ± 0.02 ^{c}	2.4 ± 0.15 ^{a} (↑)	2.29 ± 0.05 ^{ab}	2.5 ± 0.28 ^{a}
p-Coumaric acid	0.54 ± 0.03 ^{a}	0.71 ± 0.08 ^{a}	nd	nd	nd	nd	nd	nd
3-Hydroxyphenylacetic acid	0.03 ± 0.01 ^{a}	0.05 ± 0.03 ^{a}	0.05 ± 0.02 ^{a}	nd	0.06 ± 0.04 ^{a}	0.12 ± 0.03 ^{a}	0.08 ± 0.02 ^{a}	0.22 ± 0.06 ^{a}
4-Hydroxyphenylacetic acid	nd	0.35 ± 0.13 ^{cd}	0.54 ± 0.02 ^{c}	0.51 ± 0.04 ^{c}	2.98 ± 0.49 ^{ab}	2.47 ± 0.25 ^{b}	4.43 ± 0.54 ^{ab}	4.65 ± 0.19 ^{a}
3,4-Dihydroxyphenylacetic acid	nd	nd	0.06 ± 0.02 ^{a}	0.13 ± 0.04 ^{a}	0.08 ± 0.02 ^{a}	0.17 ± 0.05 ^{a}	0.02 ± 0.01 ^{a}	0.08 ± 0.01 ^{a}
Phenylacetic acid	nd	nd	nd	nd	5.63 ± 0.62 ^{a}	4.75 ± 0.29 ^{a}	6.49 ± 0.44 ^{a}	6.7 ± 1.4 ^{a}
3-(3´-Hydroxyphenyl)propionic acid	1.29 ± 0.06 ^{b}	1.63 ± 0.25 ^{ab}	1.37 ± 0.15 ^{ab}	1.95 ± 0.07 ^{a}	0.61 ± 0.01 ^{b}	1.03 ± 0.11 ^{b}	0.37 ± 0.01 ^{b}	0.95 ± 0.07 ^{b}
Phenylpropionic acid	nd	nd	22.13 ± 0.17 ^{b}	23.31 ± 1.42 ^{b}	25.62 ± 0.4 ^{b}	27.84 ± 1.47 ^{ab}	25.86 ± 0.59 ^{b}	28.85 ± 0.24 ^{a} (↑)
4-hydroxy-5-(3´-hydroxyphenyl) valeric acid	nd	nd	nd	nd	nd	0.02 ± 0.01 ^{b} (↑)	nd	0.14 ± 0.03 ^{a} (↑)
4-hydroxy-5-(3´,4´-dihydroxyphenyl) valeric ac	nd	nd	nd	nd	nd	0.04 ± 0.00 ^{a} (↑)	nd	0.082 ± 0.04 ^{a} (↑)
5-(3′-hydroxyphenyl)-γ-valerolactone	nd	nd	nd	0.03 ± 0.01 ^{a}	0.02 ± 0.01 ^{b}	0.03 ± 0.01 ^{ab}	0.02 ± 0.01 ^{b}	0.02 ± 0.01 ^{ab}
5-(3′,4′-dihydroxyphenyl)-γ-valerolactone	nd	nd	nd	0.86 ± 0.08 ^{b} (↑)	0.48 ± 0.02 ^{b}	1.19 ± 0.07 ^{a} (↑)	nd	0.28 ± 0.01 ^{c} (↑)

VOLUNTEER # 3

	0 h		6 h		24 h		48 h	
Phenolic metabolites	RW	RW + SO2	RW	RW + SO2	RW	RW + SO2	RW	RW + SO2
4-Hydroxybenzoic acid	0.48 ± 0.02 ^{c}	0.5 ± 0.01 ^{c}	0.54 ± 0.05 ^{bc}	0.64 ± 0.04 ^{abc}	0.66 ± 0.03 ^{ab}	0.79 ± 0.06 ^{a}	0.77 ± 0.06 ^{a}	0.79 ± 0.09 ^{a}
Salicylic acid	nd	nd	0.31 ± 0.01 ^{a}	0.25 ± 0.01 ^{b} (↓)	nd	0.18 ± 0.02 ^{c} (↑)	nd	nd
Protocatechuic acid	0.08 ± 0.01 ^{c}	0.16 ± 0.01 ^{b} (↑)	0.11 ± 0.01 ^{bc}	0.17 ± 0.04 ^{b}	0.25 ± 0.01 ^{b}	0.27 ± 0.01 ^{b}	0.32 ± 0.05 ^{ab}	0.34 ± 0.01 ^{a}
Vanillic acid	2.05 ± 0.09 ^{a}	2.21 ± 0.05 ^{a}	2.29 ± 0.09 ^{a}	2.25 ± 0.32 ^{a}	2.15 ± 0.03 ^{a}	2.70 ± 0.20 ^{a}	2.14 ± 0.21 ^{a}	2.79 ± 0.35 ^{a}
4-O-Methylgallic acid	nd	0.02 ± 0.01 ^{a} (↑)	nd	nd	0.01 ± 0.01 ^{b}	nd (↓)	nd	nd
Syringic acid	1.03 ± 0.13 ^{b}	1.53 ± 0.10 ^{b}	1.90 ± 0.27 ^{ab}	2.90 ± 0.02 ^{a}	0.56 ± 0.01 ^{b}	0.87 ± 0.08 ^{b}	0.82 ± 0.10 ^{b}	0.59 ± 0.03 ^{b}
p-Coumaric acid	0.42 ± 0.01 ^{a}	0.39 ± 0.03 ^{a}	nd	nd	nd	nd	nd	0.01 ± 0.01 ^{b}
3-Hydroxyphenylacetic acid	nd	nd	nd	nd	nd	nd	nd	0.15 ± 0.03 ^{a}
4-Hydroxyphenylacetic acid	1.46 ± 0.25 ^{e}	1.87 ± 0.29 ^{cde}	1.74 ± 0.16 ^{de}	2.74 ± 0.27 ^{bcd}	3.46 ± 0.01 ^{abc}	2.95 ± 0.25 ^{bc}	5.14 ± 0.31 ^{a}	3.94 ± 0.50 ^{ab}
3,4-Dihydroxyphenylacetic acid	nd	nd	0.04 ± 0.01 ^{b}	0.07 ± 0.01 ^{a} (↑)	0.11 ± 0.02 ^{a}	0.19 ± 0.02 ^{a}	0.08 ± 0.04 ^{a}	0.16 ± 0.01 ^{a}
Phenylacetic acid	nd	nd	4.38 ± 0.33 ^{b}	5.58 ± 0.95 ^{b}	27.39 ± 1.77 ^{a}	23.81 ± 2.26 ^{a}	27.04 ± 1.87 ^{a}	24.46 ± 1.57 ^{a}
3-(3´-Hydroxyphenyl)propionic acid	0.94 ± 0.01 ^{b}	1.01 ± 0.09 ^{b}	1.94 ± 0.15 ^{a}	2.64 ± 0.04 ^{a}	0.18 ± 0.01 ^{d}	0.49 ± 0.04 ^{c} (↑)	0.13 ± 0.01 ^{e}	0.25 ± 0.02 ^{d} (↑)
Phenylpropionic acid	nd	nd	nd	nd	26.38 ± 0.09 ^{b}	30.4 ± 1.66 ^{ab}	26.92 ± 0.01 ^{b}	31.32 ± 0.43 ^{a} (↑)
4-hydroxy-5-(3´-hydroxyphenyl) valeric acid	nd	nd	nd	nd	nd	0.06 ± 0.01 ^{b} (↑)	nd	0.34 ± 0.01 ^{a} (↑)
4-hydroxy-5-(3´,4´-dihydroxyphenyl) valeric ac	nd	nd	nd	nd	nd	0.12 ± 0.04 ^{a} (↑)	nd	0.12 ± 0.03 ^{a} (↑)
5-(3′-hydroxyphenyl)-γ-valerolactone	nd	0.02 ± 0.01 ^{b} (↑)	0.02 ± 0.01 ^{ab}	0.03 ± 0.01 ^{a}	0.01 ± 0.01 ^{bc}	0.02 ± 0.01 ^{ab}	0.02 ± 0.01^{ab}	0.03 ± 0.01 ^{a}
5-(3′,4′-dihydroxyphenyl)-γ-valerolactone	nd	nd	nd	0.91 ± 0.02 ^{a} (↑)	0.37 ± 0.01 ^{b}	0.29 ± 0.02 ^{c} (↓)	0.26 ± 0.08 ^{cd}	0.21 ± 0.02 ^{d}

n.d.: not detected, SW: synthetic wine, SW+SO2: synthetic wine treated with SO_2, RW: red wine, RW+SO2: red wine treated with SO_2
